# Triple-synergistic biomimetic nanoplatform orchestrates photothermal immunotherapy through coordinated ICD and STING activation

**DOI:** 10.1016/j.mtbio.2025.102497

**Published:** 2025-11-03

**Authors:** Shuyao Cai, Zhenghui Chen, Boyu Yang, Jingpei Zhang, Xinhao Zhong, Dongdong Xu, Yun Li, Yang Li, Shouchun Yin

**Affiliations:** Key Laboratory of Organosilicon Chemistry and Materials Technology of the Ministry of Education, Zhejiang Key Laboratory of Organosilicon Material Technology, College of Materials, Chemistry and Chemical Engineering, Hangzhou Normal University, Zhejiang Province, Hangzhou, 311121, PR China

**Keywords:** Biomimetic nanoparticles, Photothermal immunotherapy, Immunogenic cell death, STING pathway activation, Nano-vaccine

## Abstract

The immunosuppressive tumor microenvironment (TME) impedes conventional cancer immunotherapies. To overcome this barrier, we engineer **DDT-HM** nanoparticles (NPs), a biomimetic nanoplatform cloaked in a hybrid membrane fused from cancer cells and macrophages. This dual-functional coating combines macrophage-mediated immune evasion for prolonged circulation with cancer cell-directed homologous targeting for precise tumor accumulation. **DDT-HM** NPs co-deliver a rationally designed NIR-II photothermal agent (**TPT-Se**) and a STING agonist (DMXAA). Under 808 nm irradiation, **TPT-Se** generates localized hyperthermia that directly ablates tumors and triggers immunogenic cell death (ICD), releasing damage-associated molecular patterns (DAMPs), including cytosolic DNA. Simultaneously, tumor-localized DMXAA potently activates the STING pathway. Crucially, ICD and STING signaling exhibit potent reciprocal reinforcement: ICD-derived DAMPs amplify dendritic cell (DC) maturation, which is further potentiated by STING-driven type I interferon responses, while STING activation amplifies ICD-initiated systemic antitumor immunity. In 4T1 tumor-bearing mice, this strategy achieves remarkable suppression of both primary and distant tumors, accompanied by a 4-fold increase CD8^+^ T cells and a pro-inflammatory TME reprogramming. Furthermore, **DDT-HM** NPs function as a potent nanovaccine, expanding central and effector memory T-cell pools and conferring durable protection against tumor rechallenge. This work establishes a “triple-threat” biomimetic platform that unifies precision photothermal ablation, synergistic dual-pathway immune activation, and nanovaccine functionality for durable cancer immunotherapy.

## Graphical abstract

**Figure:**
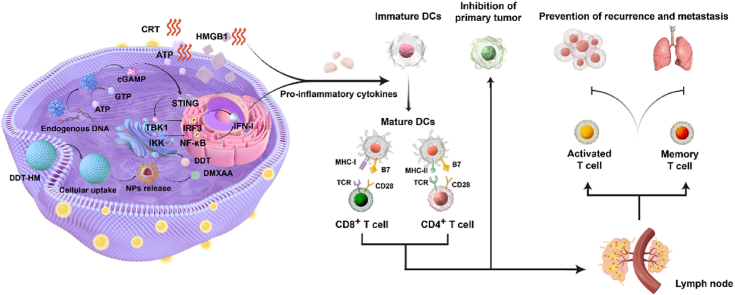

## 1 Introduction

Cancer immunotherapy, a paradigm-shifting approach harnessing the host's immune system, holds immense promise for durable tumor eradication. However, its clinical efficacy is often severely compromised by the profoundly immunosuppressive tumor microenvironment (TME), which fosters immune evasion, tolerance, and resistance [[1], [2], [3]]. Conventional cytotoxic modalities like chemotherapy and radiotherapy frequently exacerbate its immunosuppression while lacking specificity, leading to systemic toxicity and high recurrence rates [[4], [5], [6]]. Furthermore, the TME erects formidable physical and biochemical barriers that impede immune cell infiltration and function, subverting therapeutic efficacy [[7], [8], [9], [10]]. Nanomedicine has emerged as a revolutionary frontier, offering sophisticated strategies to overcome these hurdles through targeted delivery, multimodal integration, and active immune reprogramming [[11], [12], [13], [14]]. Nanoparticles (NPs) leverage the enhanced permeability and retention (EPR) effect for passive tumor accumulation while enabling stimulus-responsive localized therapies [[15], [16], [17]]. Among these, photothermal therapy (PTT) employs near-infrared (NIR)-absorbing agents to generate localized hyperthermia, directly ablating tumors and crucially, inducing immunogenic cell death (ICD) [[18], [19], [20], [21]]. ICD elicits the release of damage-associated molecular patterns (DAMPs), such as calreticulin (CRT), ATP, and high-mobility group box 1 (HMGB1), which act as potent “danger signals” to promote dendritic cell (DC) maturation and subsequent cytotoxic T lymphocyte (CTL) activation, potentially igniting systemic antitumor immunity [[22], [23], [24]]. Nevertheless, the immunosuppressive TME, dominated by regulatory T cells (Tregs), myeloid-derived suppressor cells (MDSCs), and inhibitory cytokines, frequently cripples the potency and durability of ICD-initiated immune responses, limiting efficacy against metastases and the establishment of long-term immune memory [25].

**Scheme 1:**
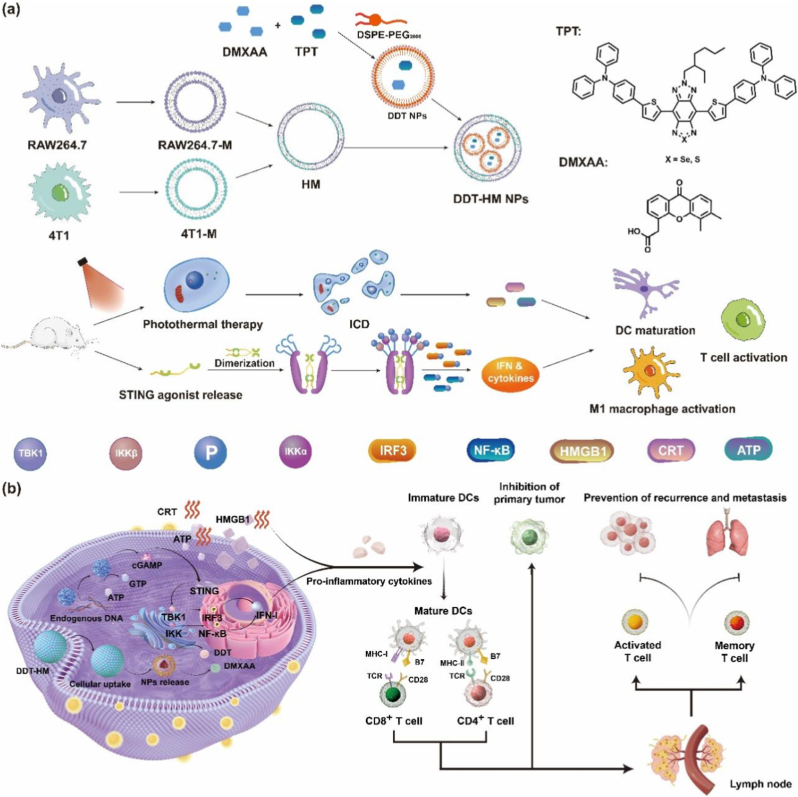
(a) Design and mechanism of **DDT-HM** NPs-mediated photothermal immunotherapy activation. (b) Schematic illustration of **DDT-HM** NPs for tumor immunotherapy created with Figdraw.

Concomitant strategies to reverse TME immunosuppression are thus paramount. Activation of the Stimulator of Interferon Genes (STING) pathway presents a compelling approach [26,27]. Cytosolic DNA sensing by cyclic GMP-AMP synthase (cGAS) activates STING, triggering TANK-binding kinase 1 (TBK1)-interferon regulatory factor 3 (IRF3) signaling and robust type I interferon (IFN) production. This cascade potently amplifies DC maturation, antigen cross-presentation, and T-cell priming, fostering a pro-inflammatory TME [[28], [29], [30]]. Despite promising preclinical results, the clinical translation of STING agonists (e.g., DMXAA, 5,6-dimethylxanthenone-4-acetic acid) faces significant hurdles: poor pharmacokinetics and bioavailability, tumor-agnostic distribution leading to off-target systemic toxicity (e.g., cytokine storms), and intrinsic TME-driven resistance mechanisms [31]. While intratumoral injection partially mitigates bioavailability issues, its impracticality for disseminated or inaccessible tumors underscores the urgent need for intelligent systemic delivery systems capable of tumor-specific targeting and activation [32].

Nanocarriers offer a promising avenue to enhance tumor accumulation of STING agonists and minimize systemic exposure [33,34]. However, conventional nanocarriers often suffer rapid clearance by the mononuclear phagocyte system (MPS) and can induce nonspecific inflammation, limiting their efficacy and safety [[35], [36], [37]]. Biomimetic nanotechnology, inspired by natural biological components, provides innovative solutions. Macrophage membrane coatings confer innate immune-evasive properties (e.g., via CD47 “don't eat me” signals), prolonging circulation and evading MPS clearance [[38], [39], [40]]. Conversely, cancer cell membranes enable homologous targeting via tumor-specific adhesion molecules, promoting preferential tumor accumulation [41,42]. Hybrid cell membrane (**HM**)-camouflaged NPs, integrating functionalities from distinct parental cells (e.g., macrophage evasion + cancer cell targeting), represent a sophisticated “dual-key” strategy to simultaneously overcome MPS clearance and poor tumor specificity [[43], [44], [45]]. While **HM** NPs have shown promise in contexts like photodynamic therapy and checkpoint blockade [[46], [47], [48], [49]], their application in orchestrating synergistic PTT-STING immunotherapy, specifically designed to capitalize on the potential reciprocal reinforcement between robust ICD induction and localized STING pathway activation, remains largely unexplored and represents a significant opportunity.

Herein, we engineer **DDT-HM** NPs, a transformative biomimetic nanoplatform designed to overcome the limitations of current monotherapies and unlock potent, systemic antitumor immunity (see Scheme 1). **DDT-HM** NPs features a core co-loaded with: (i) **TPT-Se**, a rationally engineered small-molecule NIR-II photothermal agent exhibiting exceptional photothermal conversion efficiency (PCE = 57.4 %) for deep-tissue penetration and potent ICD induction; and (ii) DMXAA, a model STING agonist. Crucially, this core is cloaked with a **HM** derived from the fusion of 4T1 breast cancer cell membranes (exploiting homologous targeting ligands like integrins) and RAW264.7 macrophage membranes (displaying immune-evasive markers like CD47). We posit that this **HM** coating bestows **DDT-HM** NPs with dual biofunctionality: macrophage membrane-mediated evasion of immune clearance for prolonged systemic circulation, coupled with cancer cell membrane-driven active homing for precise tumor accumulation. Upon localized 808 nm NIR irradiation, **TPT-Se** generates potent hyperthermia, ablating primary tumors and eliciting robust ICD, characterized by the release of DAMPs (including CRT, ATP, HMGB1, and crucially, cytosolic DNA fragments). Concurrently, tumor-accumulated DMXAA is released and activates the STING pathway within the TME. The central hypothesis driving this work is that ICD and STING activation engage in powerful crosstalk and mutual reinforcement: ICD-derived DAMPs (especially cytosolic DNA) act as natural STING agonists, amplifying DC maturation initiated by ICD signals, while STING-driven type I IFN responses dramatically potentiate the immunogenicity of ICD and enhance T-cell priming/effector functions. This synergistic dual-pathway immune activation is anticipated to effectively dismantle the immunosuppressive TME, leading to eradication of both primary and distant tumors, and establishing durable, antigen-specific immune memory. By seamlessly integrating precision photothermal ablation, synergistic dual-pathway immune activation (ICD + STING), and intrinsic nanovaccine functionality within a single, biomimetic vector, **DDT-HM** NPs addresses pivotal limitations in current cancer immunotherapy and offers a promising strategy for potent and durable tumor control.

## 2 Results and discussion

### 2.1 Rational molecular design of high-performance NIR-II photothermal agents

Achieving deep-tissue penetration for effective photothermal therapy necessitates photothermal agents (PTAs) with strong absorption within the NIR-II biological window (1000–1700 nm) [50]. To meet this critical requirement, we embarked on a rational molecular design strategy focus on small organic PTAs with intense NIR-II absorption and high PCE. The core design principle centered on a donor-acceptor-donor (D-A-D) molecular architecture, chosen for its capacity to facilitate strong intramolecular charge transfer (ICT) and enable significant bathochromic shifts into the NIR-II region [51,52]. Specifically, triphenylamine (TPA), renowned for its excellent electron-donating capability and propeller-like non-planar structure conducive to suppressing aggregation-caused quenching (ACQ), was selected as the terminal donor (D) unit. Benzobisthiadiazole (BBTD), a potent electron-deficient heterocycle, served as the central acceptor (A) core. Crucially, to maximize the ICT effect and achieve the desired deep penetration wavelengths, we implemented a strategic atomic substitution: replacing a sulfur (S) atom within the BBTD core with selenium (Se). This substitution leverages the heavier selenium atom's enhanced polarizability and stronger electron-withdrawing character, which synergistically narrows the HOMO-LUMO energy gap and promotes a pronounced redshift in absorption and emission profiles.

The synthesis commenced with the preparation of the key intermediate, **TBT-S**. This compound was synthesized via a Suzuki-Miyaura cross-coupling reaction between the core BBT-S derivative and phenylboronic acid (1:2.5 M ratio) in a toluene/H_2_O (3:1, *v*/*v*) at 90 °C for 6 h under N_2_. Purification by silica gel chromatography yielded **TPT-S** as a green solid, and ^1^H NMR spectroscopy and HR-MS unequivocally confirmed its structure (see Figs. S1–S2, Supporting Information). The target NIR-II photothermal agent, **TPT-Se**, was synthesized by first generating the selenophene-containing acceptor core, **TBT-Se**. Subsequently, **TBT-Se** underwent a Stille coupling reaction with triphenyltin (1:2.5 M ratio) in anhydrous toluene at 110 °C for 8 h under N_2_. Purification via column chromatography afforded **TPT-Se** as a dark green solid, and comprehensive ^1^H NMR and HR-MS analysis (Figs. S3–S4, Supporting Information) verified the molecular integrity: *δ* 8.83, 7.42 (thiazole-H), 4.84 (-CH_2_-), alongside multiplet signals between *δ* 7.67–7.06 corresponding to the TPA donor protons. This rational molecular engineering, combining the D-A-D scaffold with the strategic heavy-atom substitution (S → Se), was predicated on achieving two synergistic effects: (1) maximizing the ICT efficiency to drive absorption deep into the NIR-II region, enabling superior tissue penetration for imaging and therapy; and (2) optimizing non-radiative decay pathways to favor heat generation over fluorescence emission, thereby boosting PCE. As detailed in subsequent sections, this design successfully achieved an exceptional PCE of 57.4 % for **TPT-Se**, establishing it as a potent candidate for precise cancer theranostics.

### 2.2 Characterization of optical properties and nanoparticle fabrication

**Fig. 1:**
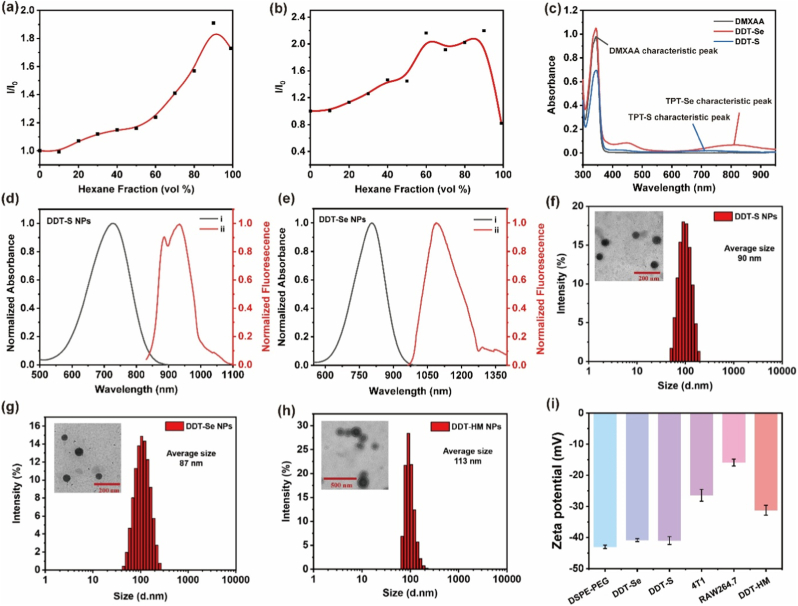
Physicochemical and optical characterization of NPs. Relative photoluminescence (PL) intensity (*I*/*I*_0_) versus *n*-hexane fraction in THF/hexane mixtures for (a) **TPT-S** and (b) **TPT-Se**, where *I*_0_ and *I* denote PL intensity maxima in pure THF and mixed solvents, respectively. (c) UV–vis absorption spectra of free DMXAA, **DDT-S** NPs and **DDT-Se** NPs. Normalized UV–vis absorption and fluorescence emission spectra of (d) **DDT-S** NPs and (e) **DDT-Se** NPs in aqueous solution. Representative TEM images and hydrodynamic size distributions of (f) **DDT-S** NPs, (g) **DDT-Se** NPs and (h) **DDT-HM** NPs. Scale bars: 100 nm. (i) Zeta potential values of DSPE-PEG2000, **DDT-Se** NPs, **DDT-S** NPs, isolated 4T1 cell membranes, isolated RAW264.7 macrophage membrane vesicles, and **DDT-HM** NPs. Data: mean ± SD (*n* = 3).

To elucidate the fundamental photophysical behavior of the synthesized **TPT** molecules (**TPT-S** and **TPT-Se**), we first investigated their absorption profiles in solution. UV–vis absorption spectroscopy revealed a profound bathochromic shift induced by the strategic selenium substitution (Fig. S5, Supporting Information). The absorption maximum (*λ*_max_) of **TPT-Se** (805 nm) exhibited a significant 78 nm redshift compared to its sulfur analogue **TPT-S** (727 nm). This substantial shift is mechanistically attributed to the heavy atom effect of selenium, which enhances ICT efficiency and effectively narrows the HOMO-LUMO energy gap, thereby extending absorption into the biologically advantageous NIR region. Furthermore, we explored the aggregation-induced emission (AIE) characteristics of both molecules. Upon increasing the fraction of the poor solvent (*n*-hexane) in THF/*n*-hexane mixtures, **TPT-S** and **TPT-Se** displayed a dramatic enhancement in relative fluorescence intensity (Fig. 1a and b). This pronounced AIE phenomenon arises from restricted intramolecular motion (RIM) in the aggregated state, which suppresses non-radiative decay pathways and promotes radiative relaxation, a crucial attribute for maintaining fluorescence efficiency in the NP formulation.

To render the hydrophobic photothermal agents (**TPT-S**, **TPT-Se**) biocompatible and enable co-delivery with the hydrophilic STING agonist DMXAA, we encapsulated them using the amphiphilic polymer 1,2-distearoyl-*sn*-glycero-3-phosphoethanolamine-*N*- [methoxy(polyethylene glycol)-2000] (DSPE-PEG2000), forming nanodelivery systems **DDT-S** NPs and **DDT-Se** NPs, respectively. Successful co-encapsulation was confirmed by UV–vis spectroscopy (Fig. 1c; Fig. S6, Supporting Information). Both **DDT-S** NPs (*λ*_max_ = 736 nm) and **DDT-Se** NPs (*λ*_max_ = 814 nm) exhibited distinct absorption peaks corresponding to their respective **TPT** molecules, alongside characteristic peaks attributable to DMXAA, confirming the integrity of the photothermal agents within the nanocarriers. Fluorescence spectroscopy provided critical insights into the emission profiles of the NPs (Fig. 1d and e). **DDT-S** NPs displayed a maximum emission wavelength (*λ*_em_) at 934 nm, residing within the NIR-I window. Strikingly, **DDT-Se** NPs exhibited a significantly redshifted *λ*_em_ at 1082 nm, representing a substantial 148 nm bathochromic shift that positions the emission firmly within the deep-tissue penetrating NIR-II biological window (1000–1700 nm). This remarkable redshift is mechanistically driven by two synergistic factors arising from the selenium substitution: (i) the enhanced electron-withdrawing capability of selenium strengthens the ICT effect within the D-A-D core, and (ii) the extended π-conjugation and reduced HOMO-LUMO energy gap collectively shift the emission to longer wavelengths. This NIR-II emission profile endows **DDT-Se** NPs with superior tissue penetration depth, making them ideal candidates for deep-tumor fluorescence imaging and precise photothermal intervention guided by high-contrast imaging.

To further enhance biocompatibility, prolong systemic circulation, and confer active tumor-targeting capabilities, the surface of the optimized **DDT-Se** NPs was cloaked with endogenous cell membranes. Specifically, a **HM** was engineered via the fusion of plasma membranes derived from 4T1 breast cancer cells (exploiting surface markers like VCAM-1α for homologous targeting) and RAW264.7 macrophages (utilizing “self” markers such as CD49d and CD47 for immune evasion). The **HM** vesicles were coated onto the pre-formed **DDT-Se** NPs using a controlled liposome extrusion technique, yielding the final biomimetic nanoplatform **DDT-HM** NPs. The successful **HM** coating was rigorously validated by dynamic light scattering (DLS) and transmission electron microscopy (TEM). DLS measurements (Fig. 1g and h) indicated a consistent increase in the average hydrodynamic diameter, from approximately 87 nm for the core **DDT-Se** NPs to about 113 nm for **DDT-HM**, consistent with the expected thickness of the biomimetic membrane envelope. TEM imaging (Fig. 1f–h) confirmed that all NPs (**DDT-S** NPs, **DDT-Se** NPs, **DDT-HM** NPs) possessed uniform, spherical morphologies with narrow size distributions, a morphology highly favorable for exploiting the EPR effect and achieving efficient tumor accumulation. Similarly, the significant height difference between **DDT-HM** and **DDT** NPs can be observed by AFM, which proves the increased particle size and obvious core-shell structure of **DDT-HM** in TEM and DLS due to the coating of cell membrane (Figs. S7–8, Supporting Information). Surface charge analysis via zeta potential measurements provided compelling evidence for the hybrid membrane encapsulation (Fig. 1i). The core DSPE-PEG2000 polymer (−43.03 mV) and the uncoated NPs (**DDT-S** NPs: −41.00 mV; **DDT-Se** NPs: −40.83 mV) exhibited strongly negative surface charges, characteristic of the PEGylated surface. In contrast, **DDT-HM** NPs displayed a significantly less negative zeta potential of −31.27 mV. This notable shift towards neutrality is attributed to the effective masking of the PEG charge by the surface components of the fused membrane vesicles, whose individual zeta potentials were measured as −26.43 mV (isolated 4T1 membrane vesicles) and −15.9 mV (isolated RAW264.7 membrane vesicles). This substantial alteration in surface charge profile serves as strong physicochemical evidence supporting the successful formation of the **HM** cloak around the **DDT-Se** NP core, essential for its dual intended biofunctionality of immune evasion and active targeting.

### 2.3 In vitro photothermal conversion performance

**Fig. 2:**
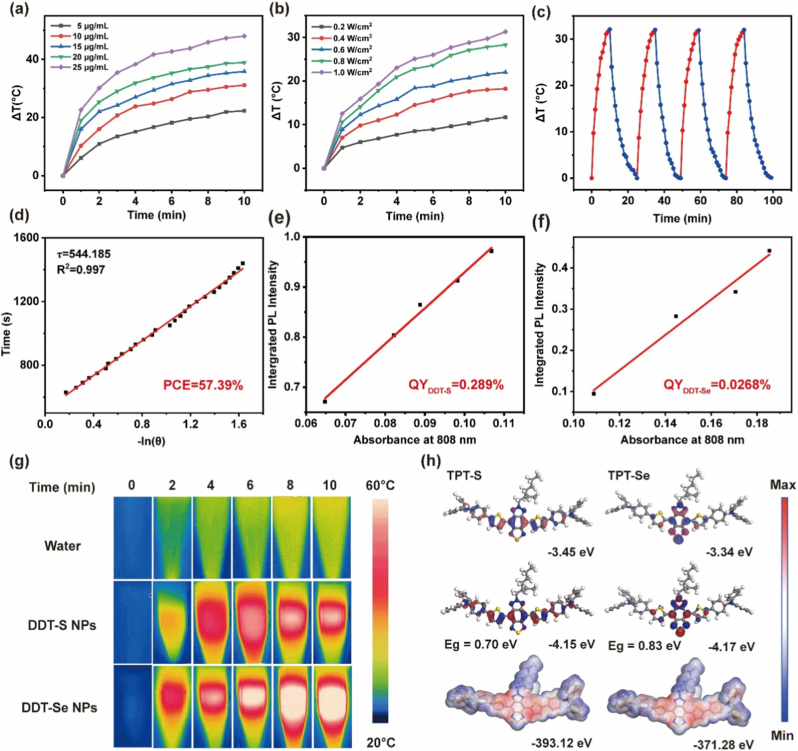
In vitro photothermal conversion performance. (a) Temperature evolution profiles of **DDT-Se** NP aqueous solutions at varied concentrations (5–25 μg/mL) under continuous 808 nm laser irradiation (1.0 W/cm^2^, 10 min). (b) Laser power density-dependent temperature profiles (0.2–1.0 W/cm^2^) for **DDT-Se** NPs (10 μg/mL; 808 nm, 10 min). (c) Temperature trajectories of **DDT-Se** NPs (10 μg/mL) over four consecutive laser ON/OFF cycles (808 nm, 0.8 W/cm^2^, 10 min/cycle). (d) Linear correlation between cooling time and -ln(θ) for **DDT-Se** NPs. Absorbance versus integrated area under the curve for (e) **DDT-S** NPs and (f) **DDT-Se** NPs under 808 nm illumination. (g) Infrared thermal images of water, **DDT-S** NPs and **DDT-Se** NPs (10 μg/mL) irradiated at 808 nm (0.8 W/cm^2^) at indicated timepoints (0–10 min). (h) Computed frontier molecular orbitals (HOMO/LUMO) and electrostatic potential (ESP) surfaces of **TPT-Se** and **TPT-S** at the DFT/B3LYP level.

Quantitative evaluation of the photothermal conversion capabilities of **DDT-S** NPs and **DDT-Se** NPs was performed by monitoring temperature profiles of aqueous solutions under continuous 808 nm laser irradiation using real-time infrared thermography. As anticipated, solutions containing both NPs exhibited significant, concentration-dependent temperature increases (ΔT) over time (Fig. 2a; Fig. S9a–b, Supporting Information), confirming efficient NIR light-to-heat conversion. Power density dependence studies further substantiated this photothermal response: irradiation of **DDT-Se** and **DDT-S** NP solutions (10 μg/mL) at varying laser intensities (0.2–1.0 W/cm^2^) revealed a pronounced power-dependent ΔT for both formulations (Fig. 2b; Fig. S9c–d, Supporting Information). Crucially, under identical conditions (808 nm, 1.0 W/cm^2^, 10 min), **DDT-Se** NPs achieved a significantly higher ΔT (32.6 °C) compared to **DDT-S** NPs (ΔT = 30.7 °C), highlighting the superior photothermal performance enabled by selenium substitution.

Photothermal stability, a critical parameter for therapeutic applications, was assessed through four consecutive heating (laser on: 0.8 W/cm^2^, 10 min) and cooling (laser off) cycles. Both **DDT-Se** and **DDT-S** NPs (10 μg/mL) demonstrated excellent stability, with maximum temperature elevations remaining remarkably consistent across all cycles (Fig. 2c; Fig. S9e, f Supporting Information). Quantitatively, the maximum temperature variation between cycles was less than 1.5 %, indicated minimal photobleaching or degradation under repeated NIR exposure. The photothermal conversion efficiency (*η*), a key metric for evaluating PTT agent performance, was rigorously quantified using the established cooling period analysis method (Supporting Information). Linear regression of the cooling time versus -lnθ relationship (Fig. 2d; Fig. S9f, Supporting Information) yielded *η* values of 57.4 % for **DDT-Se** NPs and 28.0 % for **DDT-S** NPs. This dramatic >2-fold enhancement in *η* underscores the pivotal role of the heavy atom effect (S → Se substitution) in optimizing non-radiative relaxation pathways for highly efficient heat generation.

Complementary fluorescence quantum yield (*Φ*_F_) measurements, using IR-26 as a reference standard under steady-state conditions (Fig. 2e and f; Fig. S10, Supporting Information), revealed an inverse correlation with *η*: **DDT-S** NPs exhibited a *Φ*_F_ of 0.021, nearly an order of magnitude higher than **DDT-Se** NPs (*Φ*_F_ = 0.0024). This inverse relationship is mechanistically explained by the distinct relaxation pathways favored by each molecule. While the rigid planar BBTD core minimizes vibrational loss and the D-A-D π-system promotes radiative transitions in **TPT-S**, selenium substitution in **TPT-Se** significantly enhances ICT and optimizes energy levels (Fig. 2h), favoring non-radiative relaxation and maximizing heat generation (Fig. 2g). Consequently, **TPT-Se** achieves superior PCE at the expense of fluorescence efficiency. This exceptional photothermal performance profile, characterized by high efficiency (57.4 %) and stability, established **DDT-Se** NPs as the optimal candidate for efficient in vivo photothermal ablation, and they were therefore selected for all subsequent in vitro and in vivo investigations.

### 2.4 Fabrication and functional validation of hybrid cell membranes (HMs)

**Fig. 3:**
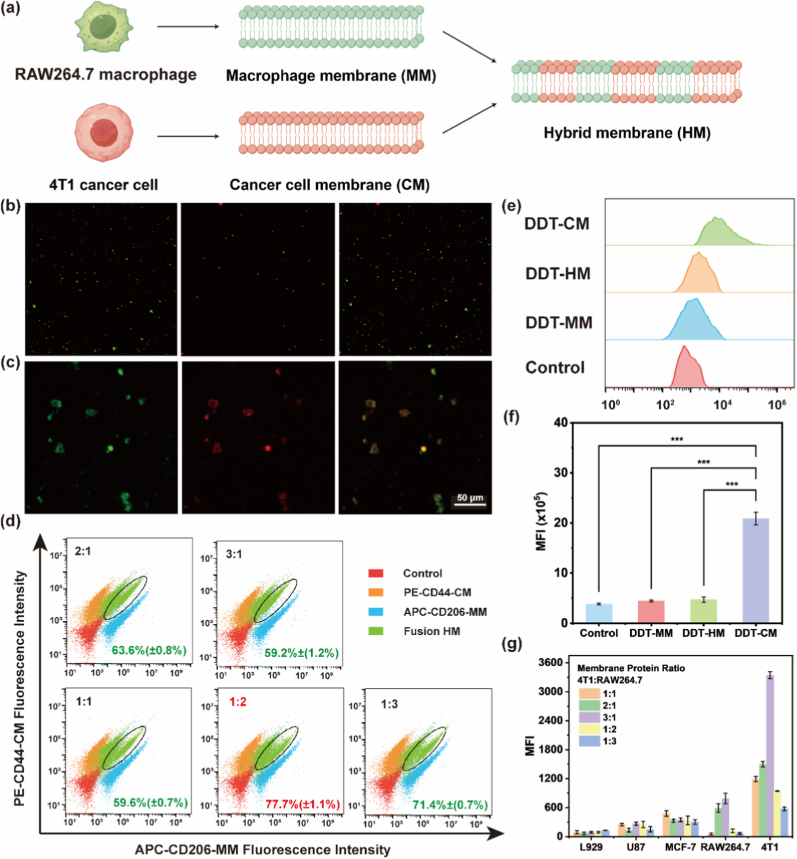
Fabrication and functional characterization of **HM**s. (a) Schematic of **HM** fabrication through fusion of plasma membranes isolated from RAW264.7 macrophages and 4T1 cancer cells (created with Figdraw). Confocal microscopy validation of membrane fusion: (b) pre-fusion state showing DiD-labeled 4T1 membranes (red) and DiO-labeled RAW264.7 membranes (green); (c) post-fusion vesicles with colocalized fluorescence (yellow). Scale bars: 50 μm. (d) Flow cytometric quantification of parental membrane protein retention on **HM**s using lineage-specific markers (CD44: 4T1; CD206: macrophages). (e) Flow cytometry histograms of RAW264.7 macrophage uptake after 4 h incubation with **DDT-CM**, **DDT-HM**, or **DDT-MM** NPs. (f) Mean fluorescence intensity (MFI) analysis of (e), demonstrating reduced uptake of **DDT-HM**/**DDT-MM** vs. **DDT-CM** (∗∗∗*p* < 0.001). (g) Cellular uptake of **DDT-HM** NPs with varying 4T1:RAW264.7 membrane protein ratios (1:3 to 3:1) across cell lines.

Cell membrane-camouflaged nanomedicines represent a promising biomimetic strategy, leveraging natural biological components to enhance biocompatibility and functionality for applications in drug delivery, immunotherapy, and diagnostics [53]. However, nanoplatforms cloaked with a single membrane type often exhibit limited functionality. To overcome this constraint and integrate complementary properties, specifically, we engineered **HM**-coated NPs (**DDT-HM**) that synergistically integrate macrophage-mediated immune evasion and cancer cell-driven homologous targeting (Fig. 3a).

**HM** fabrication commenced with isolating plasma membranes from 4T1 breast cancer cells (**CM**) and RAW264.7 macrophages (**MM**) via differential centrifugation (500×*g*, 10 min; 2000×*g*, 20 min; 100,000×*g*, 1 h at 4 °C), followed by hypotonic lysis, membrane protein quantification (BCA assay), and resuspension in PBS. To optimize fusion and confirm **HM** formation, **CM** and **MM** were differentially labeled with lipophilic fluorescent dyes DiO (green, 5 μM) and DiD (red, 5 μM), respectively. Labeled membranes were co-extruded through a 400-nm polycarbonate membrane using a lipid extruder (11 passes) at a 1:1 membrane protein ratio to yield **HM**. Confocal laser scanning microscopy (CLSM) analysis revealed extensive colocalization (yellow signal) of DiO and DiD fluorescence in fused vesicles (Fig. 3b and c), providing direct visual evidence of successful membrane fusion. Flow cytometry quantitatively assessed the retention of key membrane proteins from both parental cell types on the fused **HM**. Using specific markers, CD44 for **CM** and CD206 for **MM**, we confirmed successful protein incorporation: 84.8 % ± 1.1 % of **HM** were CD44^+^ while 86.9 % ± 2.7 % were CD206^+^ (Fig. 3d). By systematically varying the **CM**:**MM** membrane protein ratio (1:3, 1:2, 1:1, 2:1, 3:1), an optimal fusion ratio of 1:2 (**CM**:**MM**) was identified, achieving a fusion efficiency of 77.7 % ± 1.1 % based on dual-positive (CD44^+^CD206^+^) vesicle populations.

We next evaluated the critical immune evasion capability conferred by the macrophage membrane component via macrophage uptake. Flow cytometric analysis of RAW264.7 macrophage uptake after 4 h incubation revealed significantly higher fluorescence intensity for NPs coated with **CM** alone (**DDT-CM** NPs) compared to those coated with **MM** alone (**DDT-MM** NPs) or **HM** (**DDT-HM** NPs) (Fig. 3e and f). Quantitatively, the mean fluorescence intensity (MFI) of **DDT-CM** NPs were 4.7-fold higher than **DDT-MM** NPs and 4.43-fold higher than **DDT-HM** NPs (*p* < 0.001). This demonstrates that both **DDT-MM** NPs and **DDT-HM** NPs effectively evade macrophage recognition and phagocytosis, a property attributable to the presence of macrophage-derived “self” markers (e.g., CD47) on their surface. **DDT-HM** retains this essential immune evasion capability, which is crucial for prolonged systemic circulation in vivo.

Cancer cell membranes facilitate homologous targeting via tumor-specific adhesion molecules (e.g., integrins). To identify the **HM** composition optimizing both immune evasion and homologous targeting, we quantified the uptake of **DDT-HM** NPs with varying **CM**:**MM** ratios (1:3 to 3:1) by various cell lines (L929 fibroblasts, U87 glioblastoma, MCF-7 breast cancer, RAW264.7 macrophages, 4T1 breast cancer) after 4 h co-culture (Fig. 3g). Key findings emerged: (1) increasing the proportion of **MM** proteins consistently reduced NP uptake across all cell lines, including 4T1, confirming the dominant role of macrophage membrane in minimizing nonspecific internalization. (2) conversely, increasing the proportion of **CM** proteins enhanced NP uptake, with the most pronounced effect observed in homologous 4T1 cells (source of **CM**), validating the inheritance of cancer cell targeting capability. (3) A **CM**:**MM** membrane protein ratio of 3:1 provided the optimal functional balance. At this ratio, **DDT-HM** NPs demonstrated (i) efficient internalization by homologous 4T1 tumor cells (essential for targeted delivery), (ii) significantly reduced uptake by macrophages (RAW264.7) compared to **DDT-CM** NPs (promoting longer circulation), and (iii) lower uptake by non-target cell lines (L929, U87, MCF-7) compared to high **CM**-content formulations. Consequently, **DDT-HM** NPs fabricated with **CM**:**MM** = 3:1 were selected for all subsequent studies, ensuring synergistic tumor-specific accumulation and systemic stealth properties.

### 2.5 Photothermal cytotoxicity and coordinated immune pathway activation

**Fig. 4:**
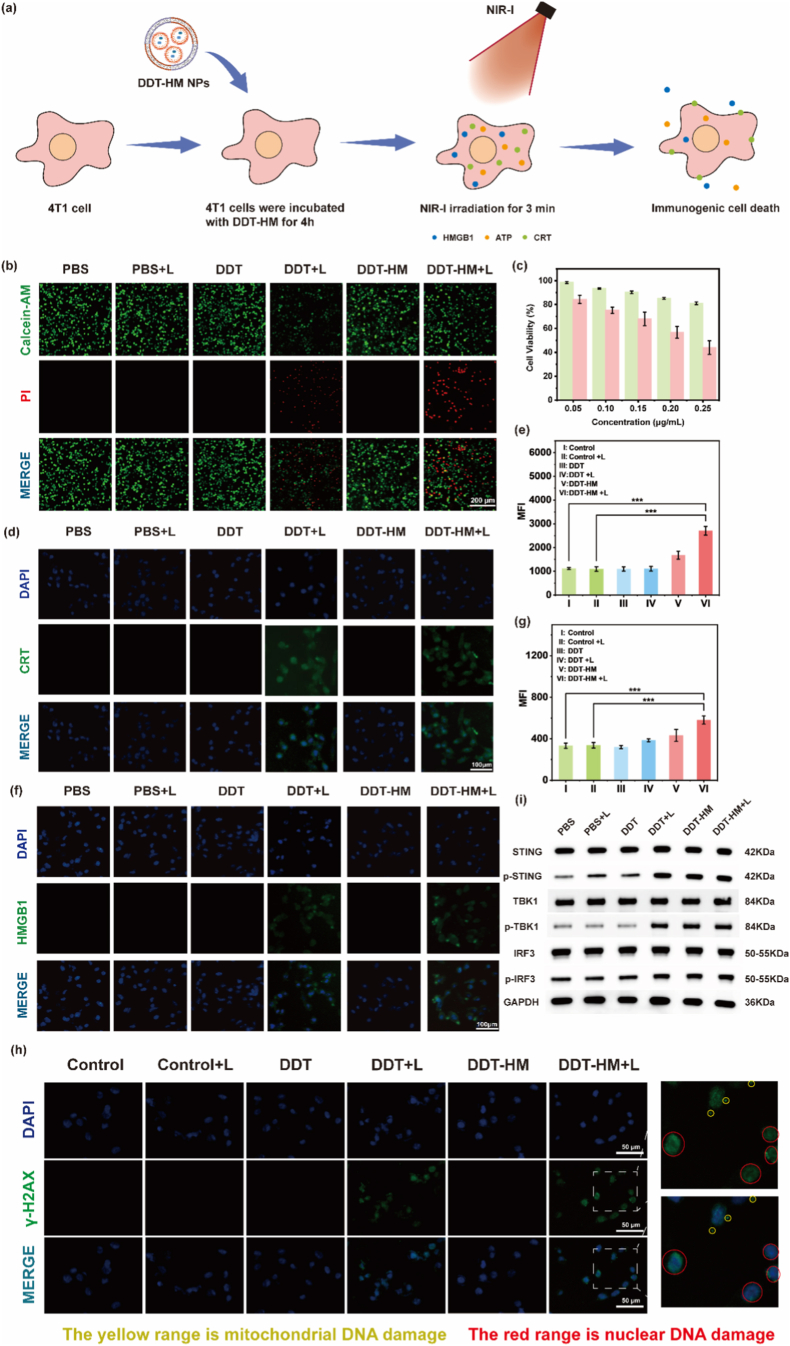
Photothermal cytotoxicity and coordinated STING pathway activation by **DDT-HM**. (a) Schematic of PTT-induced ICD mechanisms. (b) Fluorescence images of 4T1 cells treated with DMEM, **DDT** NPs or **DDT-HM** NPs ± 808 nm laser irradiation (0.8 W/cm^2^, 3 min). Viable cells (green: Calcein-AM) and dead cells (red: PI). Scale bars: 100 μm. (c) Viability of 4T1 cells treated with **DDT-HM** NPs (0.05–0.25 μg/mL) ± laser irradiation (808 nm, 0.8 W/cm^2^, 3 min). Data: mean ± SD (*n* = 5). Immunofluorescence images of (d) CRT exposure (green) and (f) HMGB1 release (green) in 4T1 cells. Nuclei: DAPI (blue). Flow cytometric quantification of (e) CRT surface expression and (g) extracellular HMGB1 efflux (∗∗∗*p* < 0.001). (h) Fluorescence microscopy of γ-H2AX (DNA damage; green) and DAPI in 4T1 cells. Scale bars: 50 μm. (i) Western blot analysis of STING pathway proteins (STING, p-STING, TBK1, p-TBK1, IRF3, p-IRF3) 24 h post-treatment. GAPDH as control.

To evaluate the biocompatibility and therapeutic efficacy of **DDT-HM** NPs (Fig. 4a), we first assessed their inherent cytotoxicity using the MTT assay. After 24 h of incubation in the absence of laser irradiation, 4T1 cell viability remained >80 % across all tested concentrations (0.05–0.25 μg/mL) (Fig. 4c), confirming negligible “dark” toxicity. In stark contrast, upon exposure to 808 nm laser irradiation (0.8 W/cm^2^, 3 min), **DDT-HM** NPs induced potent concentration-dependent photothermal cytotoxicity, reducing viability to ∼50 % at 0.20 μg/mL (Fig. 4c). In order to verify whether the coating of various cell membranes has an effect on the cytotoxicity of **DDT** NPs, we also designed a parallel test of **DDT** NPs, **DDT-MM**, **DDT-CM**, and each group was repeated multiple times to ensure the reliability of the results (Fig. S11, Supporting Information). Live/dead staining corroborated this: intense red fluorescence (propidium iodide, PI) in the **DDT-HM** + L group indicated extensive cell death, while control groups (PBS, **DDT**, or **DDT-HM** without laser; PBS + L) showed minimal cytotoxicity, with predominantly green calcein-AM staining (live cells) (Fig. 4b). These results underscore the precision of **DDT-HM** NPs-mediated photothermal ablation.

Beyond direct ablation, localized hyperthermia is a potent inducer of ICD, characterized by the spatiotemporally coordinated release of DAMPs that orchestrate antitumor immunity [[54], [55], [56], [57], [58], [59]]. Firstly, We made a single line comparison between only TPT-Se (no DMXAA) and only DMXAA (no TPT-Se) with or without laser irradiation, to dissect the individual contributions of PTT/ICD vs. STING activation (Figs. S13–14, Supporting Information).We thus systematically investigated key ICD markers triggered by **DDT-HM** NP phototherapy. Surface exposure of CRT, an endoplasmic reticulum protein translocated to the plasma membrane during ICD initiation as an “eat-me” signal, was quantified via immunofluorescence and flow cytometry (Fig. 4d and e). Robust CRT expression (green fluorescence) was observed exclusively on the surface of cells treated with **DDT-HM** + L, exhibiting a 2.44-fold increase in fluorescence intensity compared to PBS controls (*p* < 0.001). Negligible CRT exposure occurred in control groups, highlighting the specificity of **DDT-HM** NPs-driven ICD induction. We next examined the extracellular release of HMGB1, a critical DAMP that promotes DC maturation and antigen presentation via TLR4/RAGE signaling [60]. Immunofluorescence analysis revealed significant HMGB1 efflux (green) into the extracellular space specifically in the **DDT-HM** + L group, corresponding to a 1.74-fold increase relative to PBS, while other treatments showed minimal HMGB1 release (Fig. 4f and g). Furthermore, luminometric quantification of extracellular ATP secretion, a potent “find-me” signal for phagocyte recruitment and DC activation, confirmed enhanced release (1.32-fold vs. PBS, Fig. S12, Supporting Information) upon **DDT-HM** + L treatment. Collectively, these data unequivocally demonstrate robust ICD induction by the photothermal action of **DDT-HM** NPs.

To elucidate the activation of the co-delivered STING pathway, we assessed mitochondrial integrity, a potential source of cytosolic DNA triggers. JC-1 staining revealed a pronounced shift from red fluorescence (*J*-aggregates, indicating intact mitochondrial membrane potential) to green fluorescence (monomers, damaged mitochondrial membrane potential) in cells treated with the **DDT** + L and **DDT-HM** + L (Fig. S15, Supporting Information), indicating significant PTT-induced mitochondrial damage. Double-stranded DNA breaks (DSBs) in the nucleus, potent activators of the cGAS-STING axis, were detected via immunofluorescence for γ-H2AX (phosphorylated histone H2AX at Ser139), a specific marker formed upon DNA damage response activation (ATM/ATR/DNA-PK). Striking γ-H2AX fluorescence signals (green) were evident within the nuclei (DAPI, blue) of cells treated with **DDT** + L and **DDT-HM** + L, while largely absent in control groups (Fig. 4h), confirming PTT-induced nuclear DNA damage and the generation of cytosolic DNA fragments. Critically, western blot analysis provided direct evidence for **DDT-HM** + L-mediated coordinated activation of both ICD and the STING pathway (Fig. 4i, Fig. S16-17, Supporting Information). Significant upregulation of phosphorylated STING (p-STING) and its downstream effector, phosphorylated interferon regulatory factor 3 (p-IRF3), was observed. Importantly, total levels of STING, TBK1 (TANK-binding kinase 1), and IRF3 remained unchanged, confirming specific pathway activation rather than altered protein expression. After that, we verified the WB experimental results by downstream verification of the STING pathway, confirming that **DDT-HM** has an astonishing STING pathway activation effect (Fig. S18, Supporting Information). This phosphorylation cascade functionally links the photothermal damage (releasing cytosolic DNA DAMPs) and the tumor-targeted release of DMXAA to potent downstream type I interferon responses. This data strongly supports the core hypothesis that **DDT-HM** NPs orchestrate synergistic ICD induction and STING pathway activation, creating a self-reinforcing loop to amplify antitumor immunity.

### 2.6 In vivo imaging and biodistribution of DDT-HM NPs

**Fig. 5:**
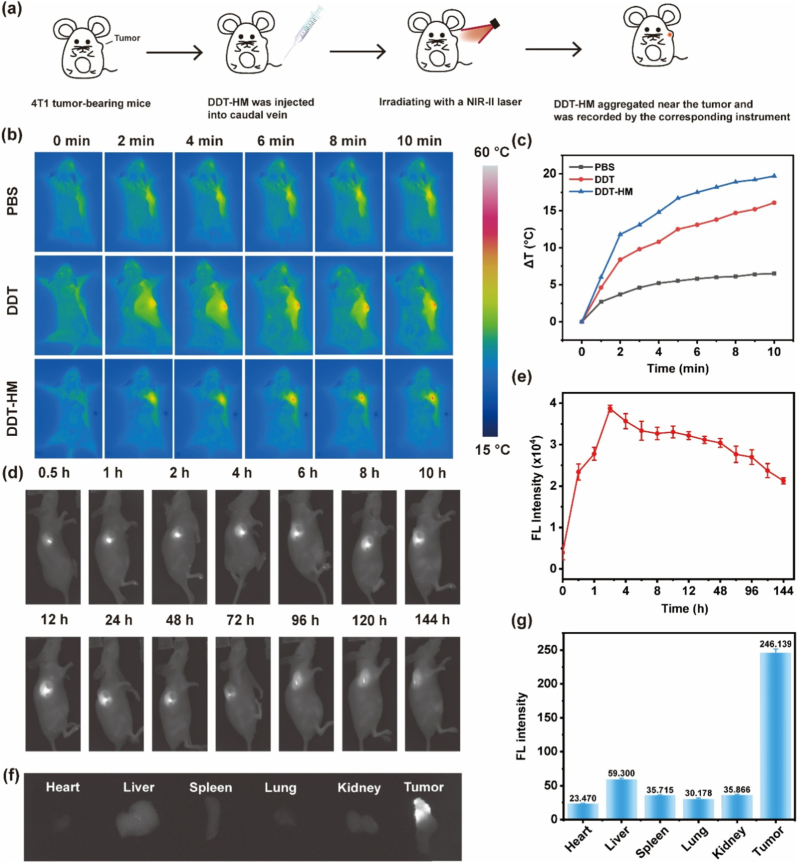
In vivo imaging and biodistribution of **DDT-HM** in 4T1 tumor-bearing mice. (a) Experimental design for in vivo imaging. (b) Infrared thermal images of tumors after intravenous injection of PBS, **DDT** NPs, or **DDT-HM** NPs with 808 nm laser irradiation (0.8 W/cm^2^, 10 min). (c) Tumor temperature kinetics during irradiation. (d) Time-dependent NIR fluorescence imaging post-intratumoral injection of **DDT-HM**. (e) Quantitative tumor fluorescence intensity from (d). (f) Ex vivo fluorescence imaging of primary tumors and major organs (heart, liver, spleen, lung, kidney) 144 h post-injection. (g) Quantified fluorescence intensities of tumors and organs from (f).

To monitor the in vivo behavior and validate the photothermal capability of **DDT-HM** NPs, we performed comprehensive real-time infrared thermal imaging and NIR fluorescence imaging in 4T1 tumor-bearing mice. First, the in vivo photothermal conversion efficacy of **DDT-HM** NPs was assessed. Mice received intravenous injections of PBS (control), unmembrane-coated **DDT** NPs, or **DDT-HM** NPs. At 4 h post-injection, the tumor region was subjected to localized irradiation (808 nm laser, 0.8 W/cm^2^). Real-time thermal imaging revealed distinct temperature profiles (Fig. 5a and b). Tumors in the **DDT-HM** group exhibited a rapid and substantial temperature increase, surging from a baseline of 33.2 °C–52.9 °C within 10 min (ΔT = 19.7 °C, Fig. 5c). Crucially, this pronounced hyperthermia generation was highly localized to the tumor site. In stark contrast, mice injected with PBS or **DDT** NPs showed only marginal temperature elevations (<5 °C under identical irradiation conditions. These results unequivocally demonstrate the superior and tumor-specific in vivo photothermal conversion capability of **DDT-HM** NPs, a critical attribute for achieving localized photothermal ablation and subsequent ICD induction.

Subsequently, we leveraged the intrinsic NIR-II fluorescence of **TPT-Se** to evaluated the tumor-targeting ability and biodistribution profile of the nanoplatform. Following intratumoral injection (to assess local retention dynamics), **DDT-HM** NPs exhibited remarkable persistence within the tumor tissue. Fluorescence intensity peaked at 2 h post-injection and remained readily detectable even at 144 h (Fig. 5d and e), indicating prolonged local retention highly favorable for sustained therapeutic action. The subsequent tail vein injection experiments also verified that **DDT-HM** NPs had homologous targeting ability and can present imaging ability at the tumor site (Fig. S19, Supporting Information). To quantify systemic distribution and preferential accumulation following intravenous administration, ex vivo fluorescence imaging of major organs (heart, liver, spleen, lung, kidney) and the primary tumor was performed at 144 h post-intratumoral injection. Strikingly, the tumor exhibited the most intense fluorescence signal, with an average intensity approximately 7-fold higher than that observed in any major organ (Fig. 5f and g). This exceptional tumor-specific accumulation and prolonged retention are directly attributed to the unique biointerface engineered by the **HM** coating. The **HM** synergistically integrates: (1) macrophage membrane-derived “self” markers (e.g., CD47), facilitating immune evasion and prolonged systemic circulation, and (2) cancer cell membrane-mediated homologous targeting ligands, promoting active tumor homing. This dual functionality enables **DDT-HM** NPs to effectively evade systemic clearance while selectively accumulating and persisting within the TME, leveraging both passive (EPR effect) and active targeting mechanisms. Collectively, these in vivo imaging studies provide compelling evidence that the biomimetic **HM** cloak of **DDT-HM** confers potent tumor-localized photothermal conversion essential for precise ablation/ICD induction and superior tumor-targeting specificity with prolonged retention, which are fundamental prerequisites for maximizing therapeutic delivery and enabling the effective photothermal-immunotherapy regimen demonstrated subsequently.

### 2.7 In vivo antitumor efficacy of DDT-HM NPs

**Fig. 6:**
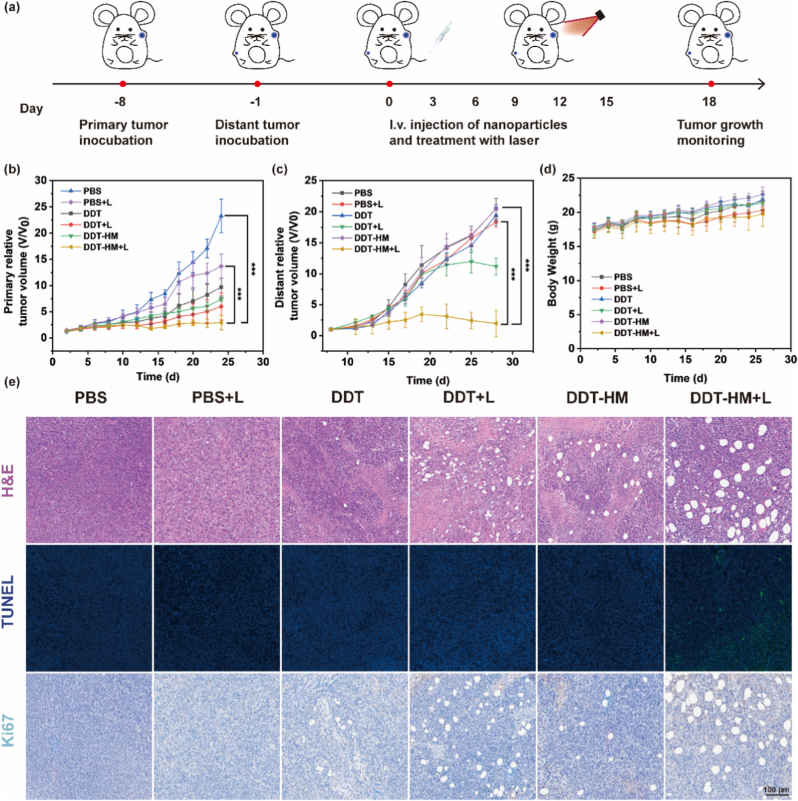
In vivo antitumor efficacy and immune activation by **DDT-HM** NPs-based photothermal-immunotherapy. (a) Bilateral 4T1 tumor model and therapeutic protocol. Primary tumors received intravenous PBS, **DDT** NPs, or **DDT-HM** NPs +808 nm laser irradiation (0.8 W/cm^2^, 3 min); distant tumors were non-irradiated. Tumor volume progression of (b) irradiated primary and (c) non-irradiated distant tumors (∗∗∗*p* < 0.001). (d) Body weight profiles during therapy. (e) H&E staining (top), TUNEL assay (apoptosis; middle), and Ki67 staining (proliferation; bottom) of primary tumors. Scale bar: 100 μm.

Capitalizing on the confirmed tumor-specific accumulation of **DDT-HM** NPs (Section 2.6), we rigorously evaluated their in vivo therapeutic efficacy against established bilateral 4T1 breast tumors in female BALB/c mice. Mice were randomly assigned to six treatment groups (*n* = 8/group): (I) PBS; (II) PBS + laser (L); (III) **DDT** NPs; (IV) **DDT** NPs + L; (V) **DDT-HM** NPs; (VI) **DDT-HM** NPs + L. Treatments were administered intravenously every three days. Four hours post-injection, primary tumors received localized 808 nm laser irradiation (0.8 W/cm^2^, 3 min), while distant tumors remained untreated to assess systemic immune effects (abscopal effect). Tumor volumes were monitored every two days for 18 d (Fig. 6a). **DDT-HM** NPs alone (Group V) induced a modest but significant delay in primary tumor growth compared to PBS controls (Group I, Fig. 6b), attributable to passive EPR-mediated accumulation and inherent immune-modulatory properties of the hybrid membrane components. **DDT** NPs + L (Group IV) demonstrated substantial growth suppression, validating the intrinsic photothermal ablation capability of the core nanoplatform. Strikingly, **DDT-HM** NPs + L (Group VI) achieved near-complete regression of primary tumors, significantly outperforming all other groups (*p* < 0.001 vs. PBS on day 18, Fig. 6b). This exceptional efficacy stems from a synergistic triad: (1) precision photothermal ablation enabled by **HM**-enhanced tumor accumulation of **TPT-Se** (*η* = 57.4 %); (2) robust ICD induction amplifying tumor antigen release; and (3) STING pathway activation via tumor-localized DMXAA release, collectively reprogramming the TME. The therapeutic impact extended robustly to non-irradiated distant tumors (Fig. 6c). While Groups I, III, and V exhibited aggressive metastatic growth, **DDT** NPs + L (IV) induced a moderate distant tumor suppression, consistent with ICD-initiated systemic immunity. Remarkably, **DDT-HM** NPs + L (VI) elicited the profound regression of distant tumors (*p* < 0.001 vs. **DDT** NPs + L on day 18, Fig. 6c), unequivocally demonstrating significantly enhanced systemic antitumor immunity driven by the synergistic coordination of photothermal ablation, amplified ICD, and localized STING pathway activation. This potent abscopal effect underscores the successful **DDT-HM** NPs-mediated reprogramming of the immunosuppressive TME into an immunogenic state.

Histopathological assessment via hematoxylin and eosin (H&E) staining of primary tumors corroborated the caliper measurements (Fig. 6e). Tumors from PBS (I), **DDT** NPs (III), and **DDT-HM** NPs (V) groups displayed densely packed, viable tumor cells with minimal architectural disruption. In contrast, tumors treated with **DDT** NPs + L (IV) exhibited substantial areas of cellular damage, including nuclear pyknosis and karyorrhexis. The most extensive destruction, characterized by widespread coagulative necrosis and loss of cellular integrity, was observed in the **DDT-HM** NPs + L group (VI), aligning precisely with its superior therapeutic outcome. Complementary terminal deoxynucleotidyl transferase dUTP nick end labeling (TUNEL) staining confirmed the highest rate of apoptosis in this group, while immunohistochemical staining for the proliferation marker Ki67 revealed the lowest proliferative activity (Fig. 6e), collectively underscoring the critical role of combined photothermal ablation and **DDT-HM** NPs-driven immune potentiation. High biocompatibility is paramount for clinical translation. Throughout the 18-day treatment period, no significant body weight loss was observed in any group (Fig. 6d), indicating negligible systemic toxicity. Comprehensive hematological analysis, serum biochemical profiling (Fig. S21, Supporting Information), and histological examination of major organs (heart, liver, spleen, lung, kidney) via H&E staining (Fig. S20, Supporting Information) revealed no significant abnormalities or signs of organ damage compared to PBS controls. These findings collectively affirm the excellent in vivo biosafety profile of the **DDT-HM** nanoplatform.

### 2.8 In vivo immune activation and memory response mediated by DDT-HM NPs

**Fig. 7:**
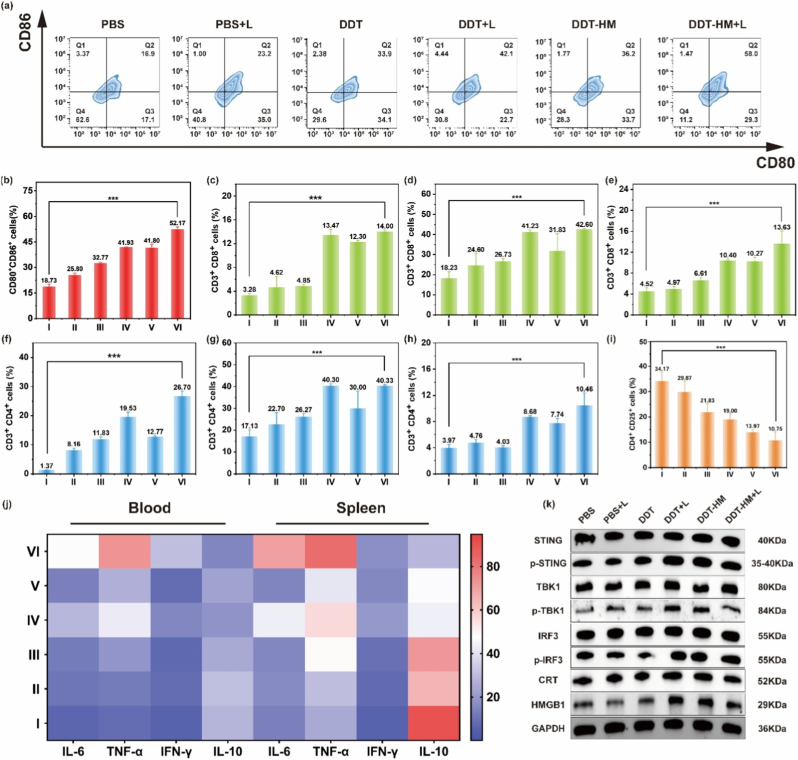
Systemic immune activation and STING pathway engagement by **DDT-HM** NPs. Analysis 16 d post-treatment. (a) Flow cytometry and (b) quantification of DC maturation (CD11c^+^CD80^+^CD86^+^) in primary tumors. CD3^+^CD8^+^ T-cell infiltration in (c) blood, (d) spleen, and (e) primary tumors. CD3^+^CD4^+^ T-cell infiltration in (f) blood, (g) spleen, and (h) primary tumors. (Groups: I: PBS; II: PBS + L; III: **DDT** NPs; IV: **DDT** NPs + L; V: **DDT-HM** NPs; VI: **DDT-HM** NPs + L). (i) Flow cytometric quantification of splenic CD4^+^CD25^+^ T_regs_. (j) Heatmap of serum and splenic cytokine expression (IL-6, TNF-α, IFN-γ, IL-10). (k) Western blot of STING pathway components (STING, p-STING, TBK1, p-TBK1, IRF3, p-IRF3) and ICD markers (CRT, HMGB1) in tumor lysates harvested 15 d post-treatment.

To elucidate the systemic immunomodulatory mechanisms underlying the potent antitumor efficacy of **DDT-HM** NPs, we comprehensively characterized DC maturation, T-cell infiltration, cytokine profiles and STING pathway engagement in tumor-bearing mice. Flow cytometry revealed robust DC maturation (CD11c^+^CD80^+^CD86^+^) within primary tumors of the **DDT-HM** NPs + L group, exhibiting a 3.43-fold increase compared to PBS controls (Fig. 7a and b; Fig. S22, Supporting Information). This maturation synergized with a 4-fold elevation in tumor-infiltrating CD8^+^ CTLs (Fig. 7c–e) and expand CD4^+^ T-cell populations (11.9 % vs. <5 % in controls; Fig. 7f–h). Systemic immune activation was further evidenced by increased CD8^+^/CD4^+^ T-cell frequencies in the spleen and peripheral blood (Fig. S23–S30, Supporting Information). Critically, **DDT-HM** NPs + L treatment markedly reduced immunosuppressive regulatory T cells (T_regs_; CD4^+^CD25^+^FoxP3^+^) in tumors and spleen by >50 % (Fig. 7i; Fig. S30, Supporting Information), effectively mitigating T_reg_-mediated suppression of effector immunity.

To assess the immunogenic reprogramming of the TME, we quantified key cytokines. **DDT-HM** NPs + L treatment significantly upregulated pro-inflammatory cytokines (IL-6, TNF-α, and IFN-γ) in serum and splenic homogenates while concurrently suppressing the anti-inflammatory cytokine IL-10 (Fig. 7j), indicating a potent Th1-skewed response aligned with the STING-driven type I interferon production. Western blot analysis of tumor lysates confirmed **DDT-HM** NPs-mediated STING pathway activation in vivo, with significant phosphorylation of STING (p-STING) and IRF3 (p-IRF3) ( Figs. 7k; Figs. S31-32, Supporting Information). Total STING, TBK1, and IRF3 levels remained unchanged, confirming pathway-specific activation rather than transcriptional upregulation. Concurrent upregulation of ICD markers (CRT, HMGB1; Fig. 7k) further underscored the synergistic loop wherein photothermal damage releases cytosolic DNA DAMPs that potentiate STING signaling, while STING activation amplifies ICD-initiated DC cross-priming.

**Fig. 8:**
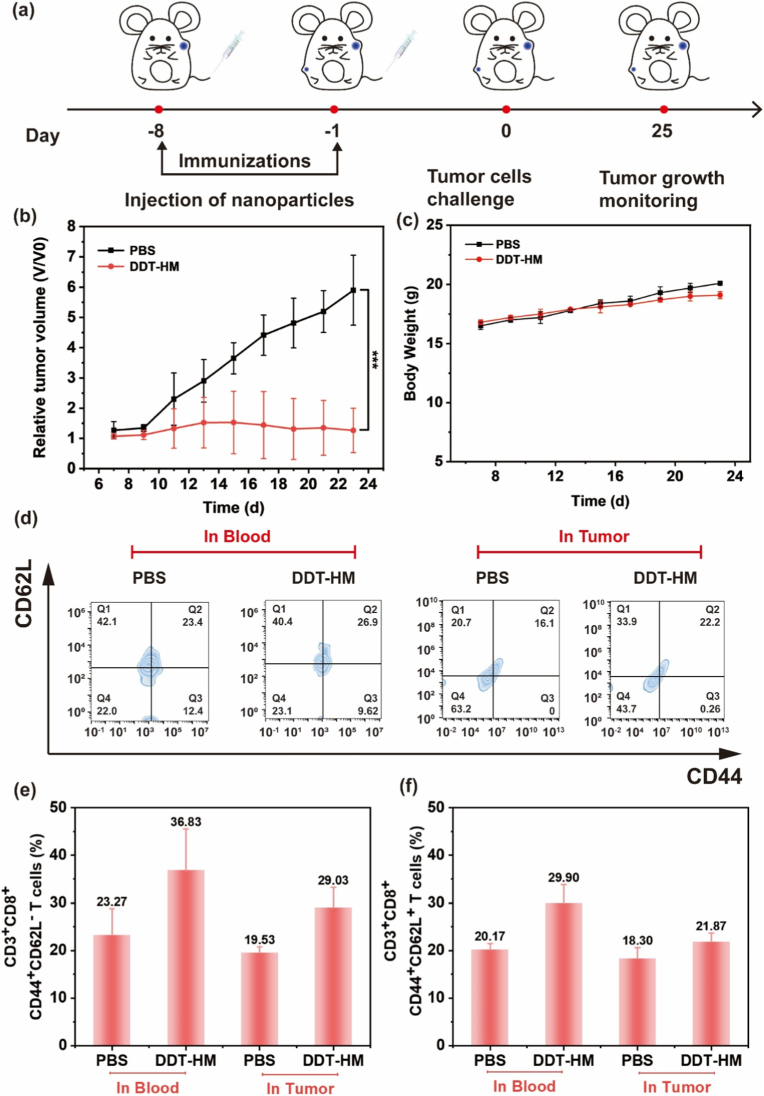
Prophylactic antitumor immunity and memory T-cell response induced by **DDT-HM** NPs nanovaccination. (a) Vaccination protocol prior to tumor challenge. (b) Tumor growth kinetics in mice pre-immunized with PBS (Group I) or **DDT-HM** NPs (Group II; *n* = 8; mean ± SD), (∗∗∗*p* < 0.001). (c) Body weight trajectories of vaccinated mice. (d) Flow cytometric analysis of CD3^+^CD8^+^ T-cell memory subsets in blood and tumors 18 d post-tumor inoculation. Quantitative analysis of (e) effector memory (T_EM_: CD44^+^CD62L^−^) and (f) central memory (T_CM_: CD44^+^CD62L^+^) T cells within CD3^+^CD8^+^ populations.

To evaluate the nanovaccine functionality of **DDT-HM** NPs, mice were immunized intravenously prior to 4T1 tumor inoculation (Fig. 8a; Fig. S33, Supporting Information). Vaccinated mice exhibited significantly delayed tumor growth (21.3 % reduction in volume vs. PBS controls at Day 25; *p* < 0.001; Fig. 8b) and expanded pools of antigen-specific memory T cells. Flow cytometry revealed substantial increases in both central memory (T_CM_: CD3^+^CD8^+^CD44^+^CD62L^+^) and effector memory (T_EM_: CD3^+^CD8^+^CD44^+^CD62L^−^) subsets in the blood and tumors (Fig. 8d–f; Fig. S34, Supporting Information). This durable memory response, coupled with negligible body weight changes (Fig. 8c), unequivocally confirms **DDT-HM** NPs as a prophylactic nanoplatform conferring long-term antitumor immunity.

## 3 Conclusion

In summary, we engineered **DDT-HM** NPs, a biomimetic nanoplatform that orchestrates potent synergistic photothermal immunotherapy through coordinated ICD and STING pathway activation. The core innovation resides in integrating a rationally designed NIR-II photothermal agent (**TPT-Se**, *η* = 57.4 %) and a STING agonist (DMXAA) within a NP cloaked by a **HM** derived from fused cancer cells and macrophages. This **HM** coating confers dual biofunctionality: macrophage membrane-mediated immune evasion for prolonged systemic circulation, coupled with cancer cell membrane-driven homologous targeting for precise tumor accumulation. Upon localized NIR irradiation, **DDT-HM** NPs achieve efficient tumor ablation and concurrently triggers robust ICD, characterized by the release of DAMPs, including cytosolic DNA. Crucially, the tumor-localized DMXAA delivery potently activates the STING pathway. This study elucidates a self-reinforcing immunologic loop: ICD-derived DAMPs (e.g., cytosolic DNA) amplify dendritic cell maturation, while STING-driven type I interferon responses potentiate ICD immunogenicity and enhance T-cell priming. This dual-pathway synergy dismantles the TME, enabling profound systemic antitumor immunity. In vivo validation using a bilateral tumor model demonstrates near-complete regression of primary tumors and significant suppression of distant tumors (abscopal effect), underpinned by a 4-fold increase in tumor-infiltrating CD8^+^ T cells and pro-inflammatory TME reprogramming. Furthermore, **DDT-HM** NPs function as a potent nano-vaccine, expands central and effector memory T-cells and confers durable protection against tumor rechallenge. Collectively, **DDT-HM** NPs exemplifies a “triple-synergistic” strategy unify: (1) precision tumor targeting and ablation via biomimetic delivery and deep-tissue-penetrating phototherapy; (2) synergistic dual-pathway immune activation (ICD + STING) to reprogram the immunosuppressive TME; and (3) intrinsic nano-vaccine functionality for sustained immune surveillance. This work establishes a compelling and highly translatable paradigm for combinatorial cancer immunotherapy, offering a promising avenue towards potent and durable tumor control.

## CRediT authorship contribution statement

**Shuyao Cai:** Writing – original draft, Software, Resources, Methodology, Investigation, Formal analysis, Data curation. **Zhenghui Chen:** Software, Methodology, Investigation, Formal analysis, Data curation. **Boyu Yang:** Methodology, Investigation, Formal analysis, Data curation. **Jingpei Zhang:** Methodology, Formal analysis, Data curation. **Xinhao Zhong:** Software, Investigation, Formal analysis. **Dongdong Xu:** Validation, Methodology. **Yun Li:** Resources, Methodology. **Yang Li:** Writing – review & editing, Software, Resources, Investigation, Funding acquisition, Formal analysis. **Shouchun Yin:** Writing – review & editing, Supervision, Resources, Project administration, Funding acquisition, Conceptualization.

## Declaration of competing interest

The authors declare that they have no known competing financial interests or personal relationships that could have appeared to influence the work reported in this paper.

## Data Availability

Data will be made available on request.
