# Supplementary material for: Triple-synergistic biomimetic nanoplatform orchestrates photothermal immunotherapy through coordinated ICD and STING activation
Source: Mater Today Bio. 2025 Nov 3;35:102497. doi: 10.1016/j.mtbio.2025.102497 (PMC12859673; doi:10.1016/j.mtbio.2025.102497)
Supplement: Multimedia component 1 [file mmc1.docx]

Supporting Information

Triple-Synergistic Biomimetic Nanoplatform Orchestrates Photothermal Immunotherapy through Coordinated ICD and STING Activation

Shuyao Cai, Zhenghui Chen, Boyu Yang, Jingpei Zhang, Xinhao Zhong, Dongdong Xu, Yun Li, Yang Li,* Shouchun Yin*

*Key Laboratory of Organosilicon Chemistry and Materials Technology of the Ministry of Education, Zhejiang Key Laboratory of Organosilicon Material Technology, College of Materials, Chemistry and Chemical Engineering, Hangzhou Normal University, Zhejiang Province, Hangzhou 311121, P. R. China*

*E-mail: liyang@hznu.edu.cn; yinsc@hznu.edu.cn*

1. Materials and methods

All reagents used in this study were of analytical grade and used without further purification. 4-(Diphenylamino)phenylboronic acid, *N*, *N*-diphenyl-4-(tributeylstannyl)benzenamine, tetrakis(triphenylphosphine)palladium and IR-26 were purchased from Energy Chemical. DMXAA was obtained from bidepharm.com. 3-(4,5-Dimethylthiazol-2-yl)-2,5-diphenyltetrazolium bromide (MTT) and trypsine-EDTA were obtained from Biomics Biotechnologies Co. Ltd. (Nantong, China). The Live/Dead Cell Staining Kit and Annexin V-FITC/PI Apoptosis Detection Kit were procured from KeyGEN Bio TECH Co. Ltd. (Jiangsu, China). Fetal bovine serum (FBS) and Dulbecco’s Modified Eagle Medium (DMEM) were sourced from Sijiqing (Hangzhou, China). 3,3’-Dioctadecyloxacarbocyanine perchlorate (DiO), 1,1’-dioctadecyl-3,3,3’,3’-tetramethylindodicarbocyanine perchlorate (DiD), the BCA Protein Assay Kit, ATP Assay Kit, Membrane and Cytosol Protein Extraction Kit, and ELISA kits for TNF-α, IFN-γ, IL-10, IL-6 detection were sourced from Beyotime Biotechnology (Shanghai, China). Antibodies against CALR, HMGB1, CD206-APC, CD8a-PE, CD3-FITC, CD4-PerCP, CD44-PE/CY7, CD80-APC, CD86-PE, and CD11c-FITC were acquired from Dakewei Biotechnology (Shenzhen, China).

^1^H NMR spectra were acquired on a Bruker Advance 500 MHz spectrometer (^1^H: 500 MHz) at 298 K. UV-vis absorption spectra were recorded on a Hitachi U-5300 spectrophotometer. Fluorescent emission spectra were obtained using an Edinburgh Instruments FLS 980 fluorescence spectrophotometer. Transmission electron microscope (TEM) images were captured on a Hitachi S-4800 microscope. Dynamic light scattering (DLS) and zeta potential measurements were performed using a Malvern Zeta size Nano ZS90. Flow cytometry was conducted on a Beckman Coulter CytoFLEXS instrument. Cellular fluorescence imaging was performed using a Nikon Eclipse Ti2 inverted fluorescence microscope and a Zeiss LSM 800 confocal laser scanning microscope (CLSM). Thermal IR images were acquired using an FLIR ONEPro IR thermal camera. Photodynamic and photothermal experiments were conducted using an 808 nm fiber-coupled laser (Ningbo Fingco Optoelectronic Co. Ltd., Ningbo, China).

2. Synthetic procedures

2.1. Synthesis


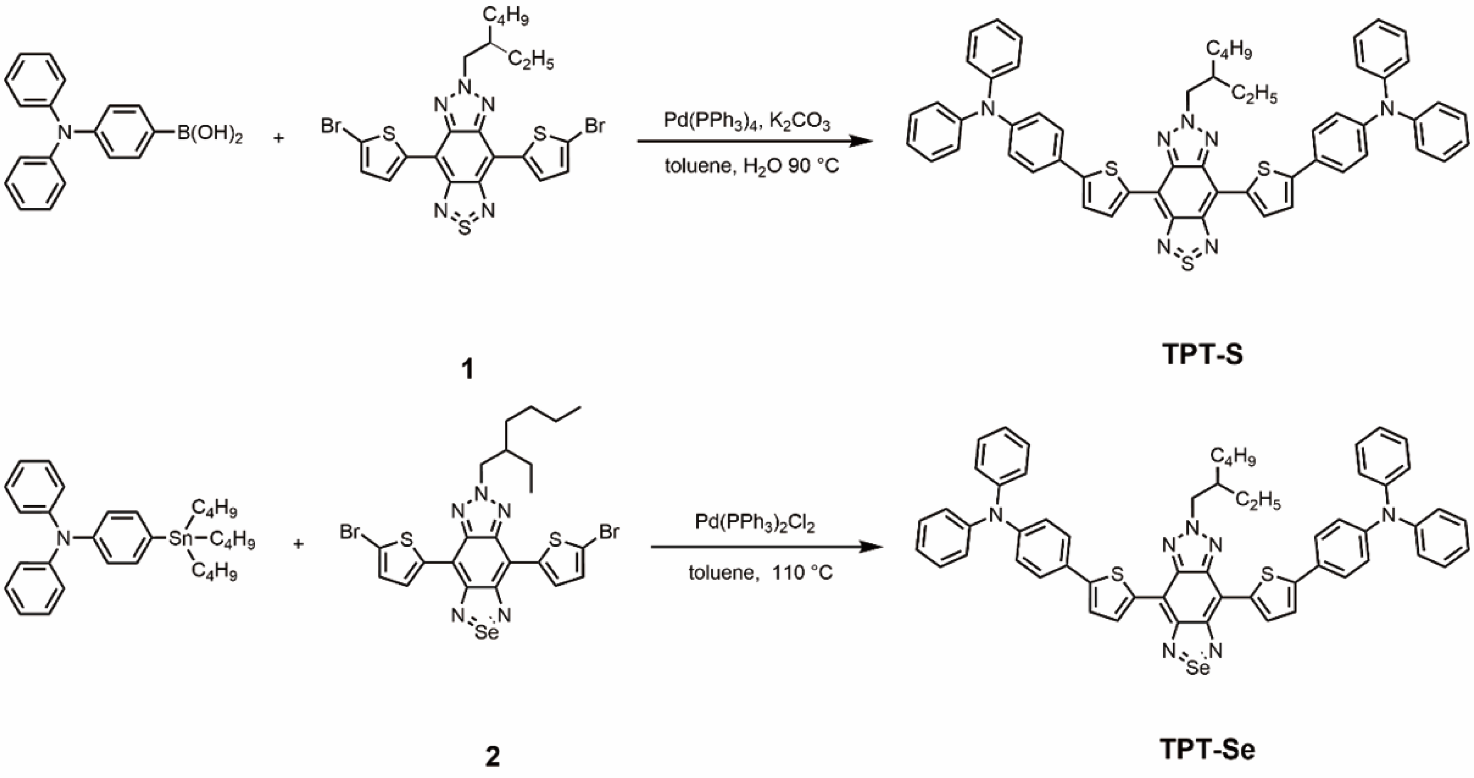


***Scheme S1*** Synthetic routes of **TPT-Se** and **TPT-Se**.

2.1.1. Synthesis of TPT-S


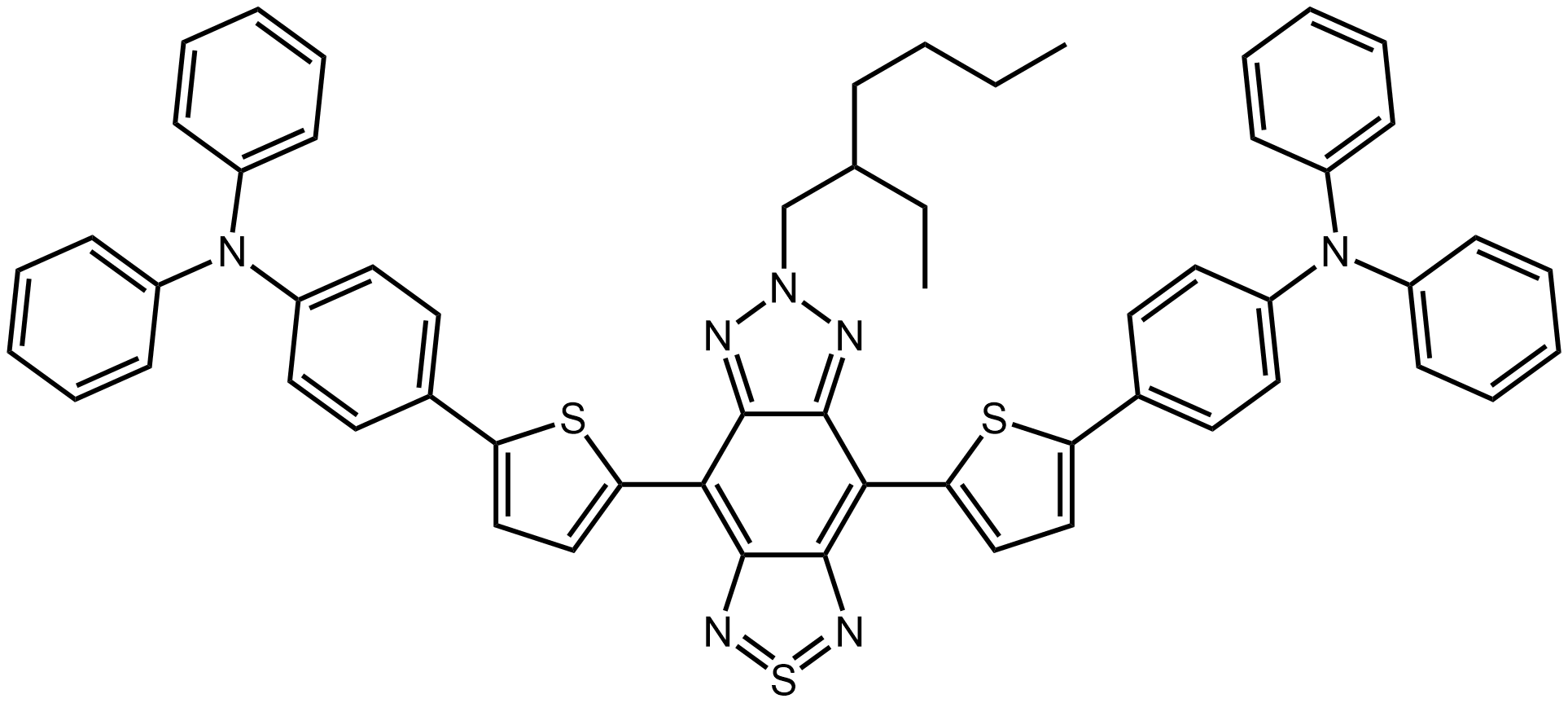
Compound **1** (200 mg, 0.33 mol) and 4-(diphenylamino) phenylboronic acid (130 mg, 0.45 mol) were dissolved in a degassed mixture of toluene and deionized water (40 mL, 3:1 *v*/*v*). The reaction mixture was transferred to a Schlenk flask and stirred at 90 °C for 6 h under N_2_. The organic layer was separated, dried over anhydrous MgSO_4_, filtered, and concentrated under reduced pressure to afford the crude product. Purification by column chromatography on silica gel using dichloromethane/petroleum ether (1:3 *v*/*v*) as the eluent yielded compound **TPT-S** as a green solid (Yield: 23.46 mg, 78.05%). ^1^H NMR (500 MHz, CDCl_3_, 298 K) *δ* (ppm): 8.84 (d, *J* = 3.6 Hz, 2H), 7.66 (d, *J* = 8.4 Hz, 4H), 7.44 (d, *J* = 3.6 Hz, 2H), 7.30 (m, 8H), 7.16 (m, 8H), 7.12 (d, *J* = 8.8 Hz, 4H), 7.06 (d, 4H), 4.85 (d, *J* = 5.2 Hz, 2H), 2.41 (s, 1H), 2.22(d, 2H), 2.01(d,2H), 1.07 (t, 3H), 0.93 (m, 7H). ESI-HRMS [Compound **S**]^+^: calculated for [C58H49N7S3]^+^ 939.3212, found 939.3265.


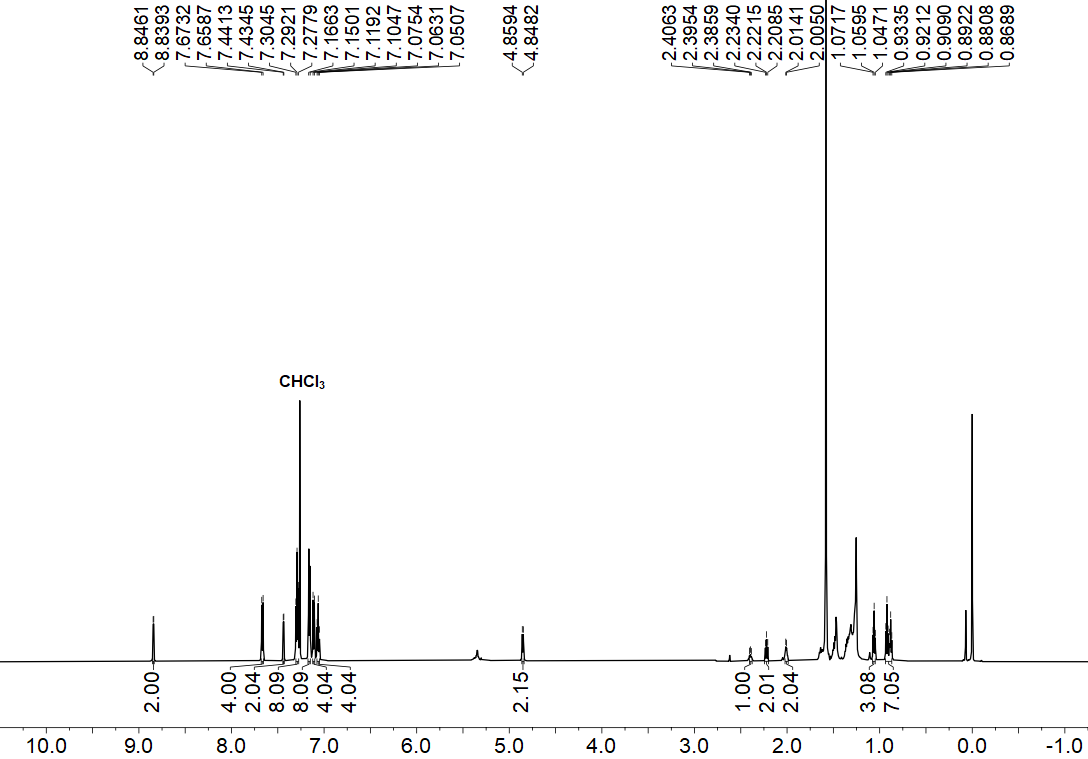


***Figure S1*** ^1^H NMR spectrum (500 MHz, CDCl_3_, 298 K) recorded for **TPT-S**.


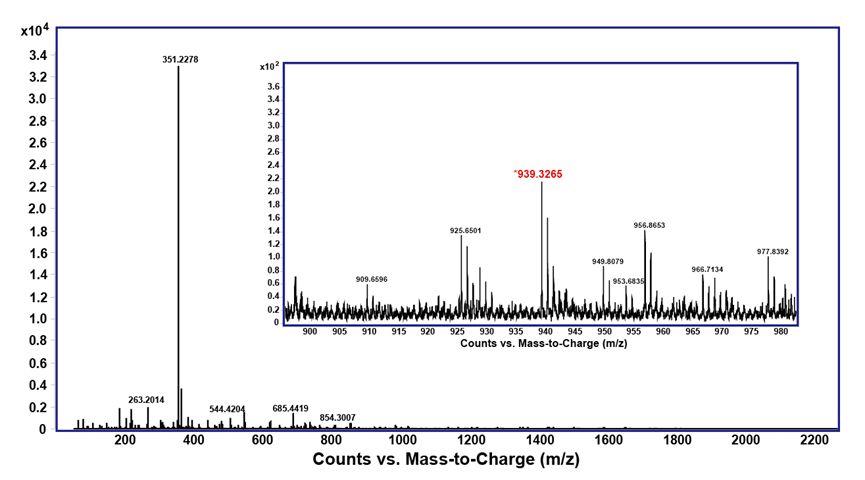


***Figure S2*** ESI-HRMS spectrum of **TPT-S**.

***2.1.2 Synthesis of TPT-Se***


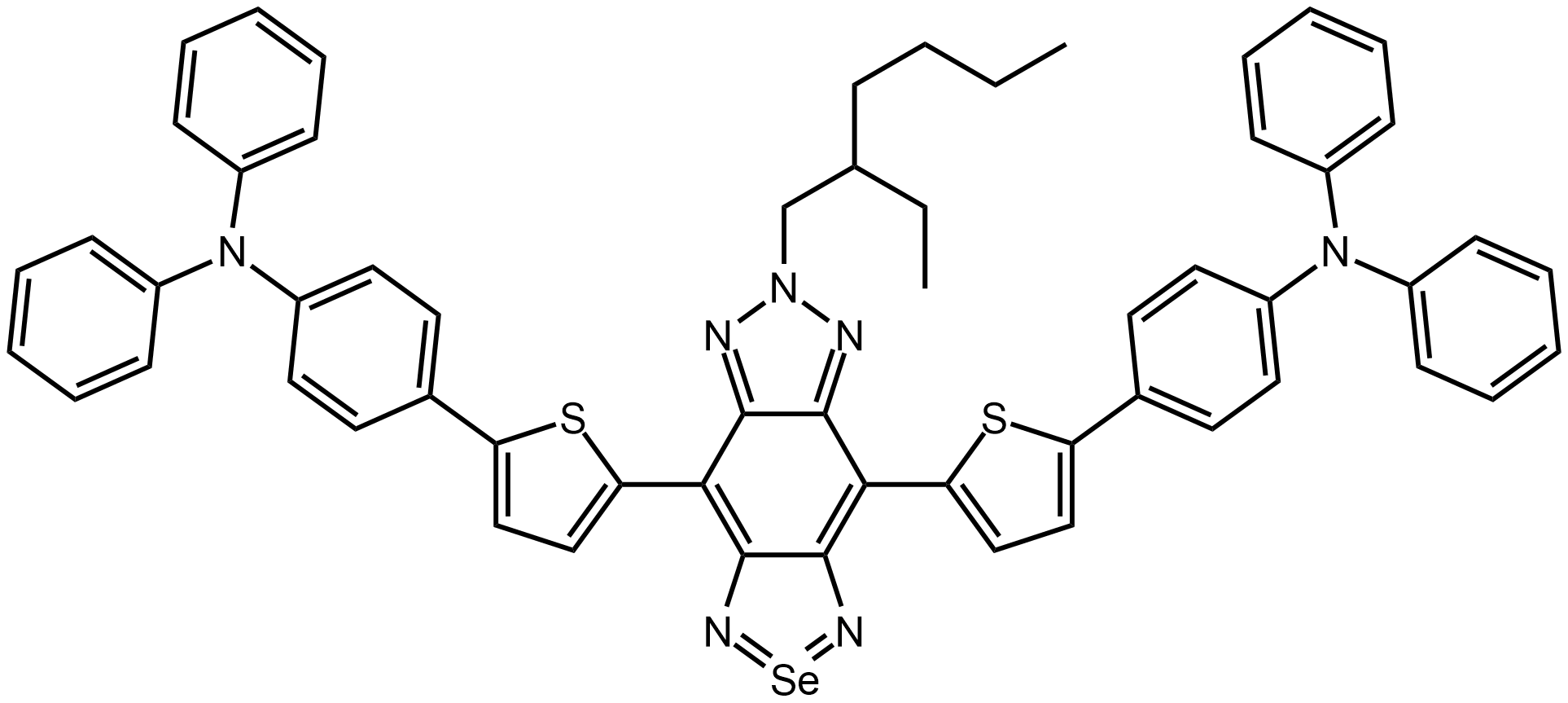
Compound **2** (200 mg, 0.3 mol) and (4-(diphenylamino)phenyl)stannane (220 mg, 0.41 mol) were dissolved in degassed toluene (30 mL). The reaction mixture was transferred to a Schlenk flask and stirred at 110 °C for 8 h under N_2_. After cooled to room temperature, the mixture was extracted with dichloromethane/water. The organic phase was separated, dried over anhydrous MgSO_4_, and concentrated under reduced pressure to afford the crude product. Purification by column chromatography on silica gel using dichloromethane/petroleum ether (1:3 *v*/*v*) as the eluent yielded compound **TPT-Se** as a green solid (Yield: 22.29 mg, 72.26%). ^1^H NMR (500 MHz, CDCl_3_, 298 K) *δ* (ppm): 8.83 (d, *J* = 3.6 Hz, 2H), 7.67 (d, *J* = 8.4 Hz, 4H), 7.42 (d, *J* = 3.6 Hz, 2H), 7.28 (m, 8H), 7.16 (m, 8H), 7.11 (d, *J* = 8.8 Hz, 4H), 7.06 (d, 4H), 4.84 (d, *J* = 5.2 Hz, 2H), 2.38 (d, 2H), 2.21(s, 1H), 2.01(d,2H), 1.07 (m, 5H), 0.93 (m, 5H). ESI-HRMS [Compound-**Se**]^+^: calculated for [C58H49N7S2Se]^+^ 981.2716, found 981.2652.


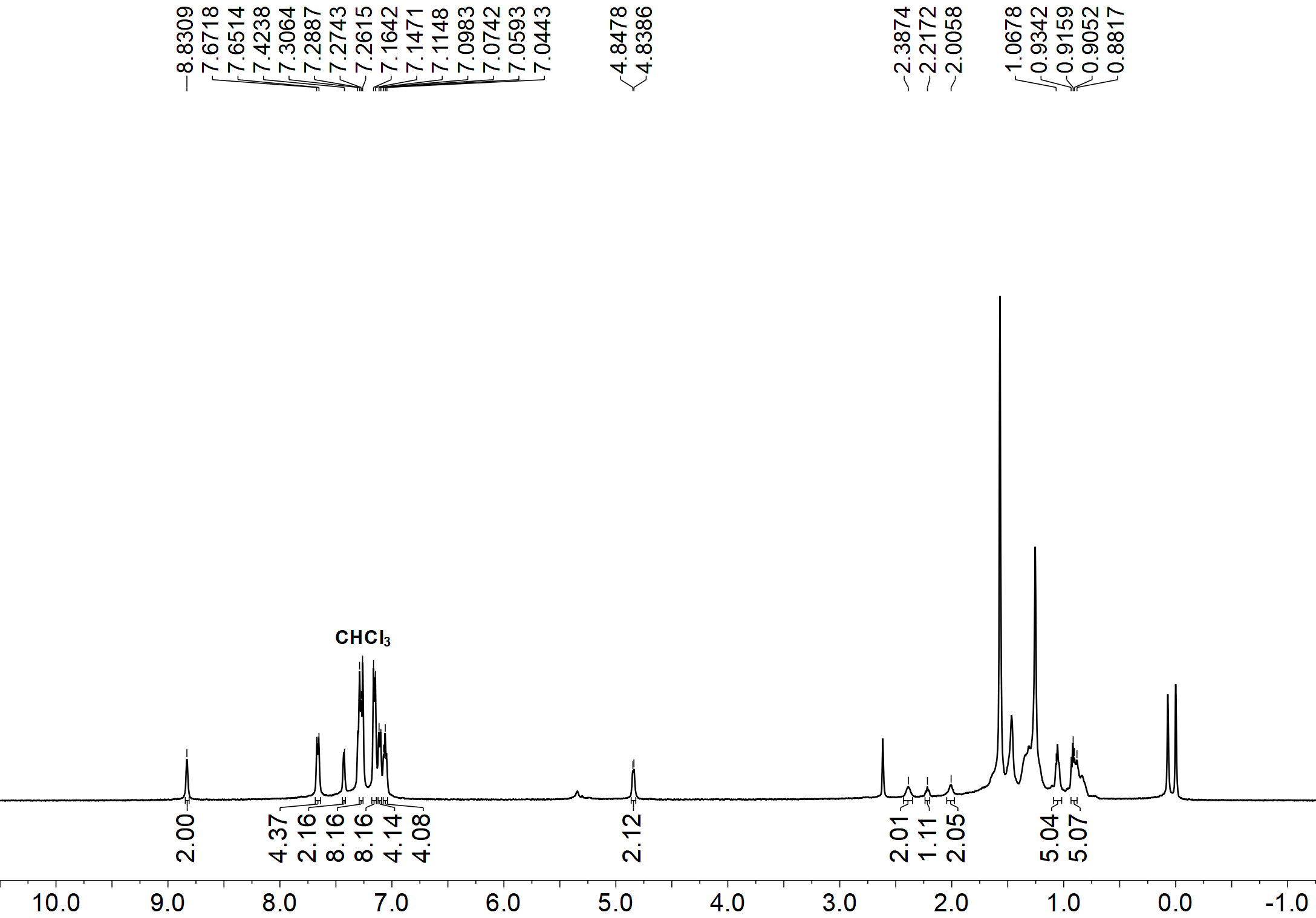


***Figure S3*** ^1^H NMR spectrum (500 MHz, CDCl_3_, 298 K) recorded for **TPT-Se**.


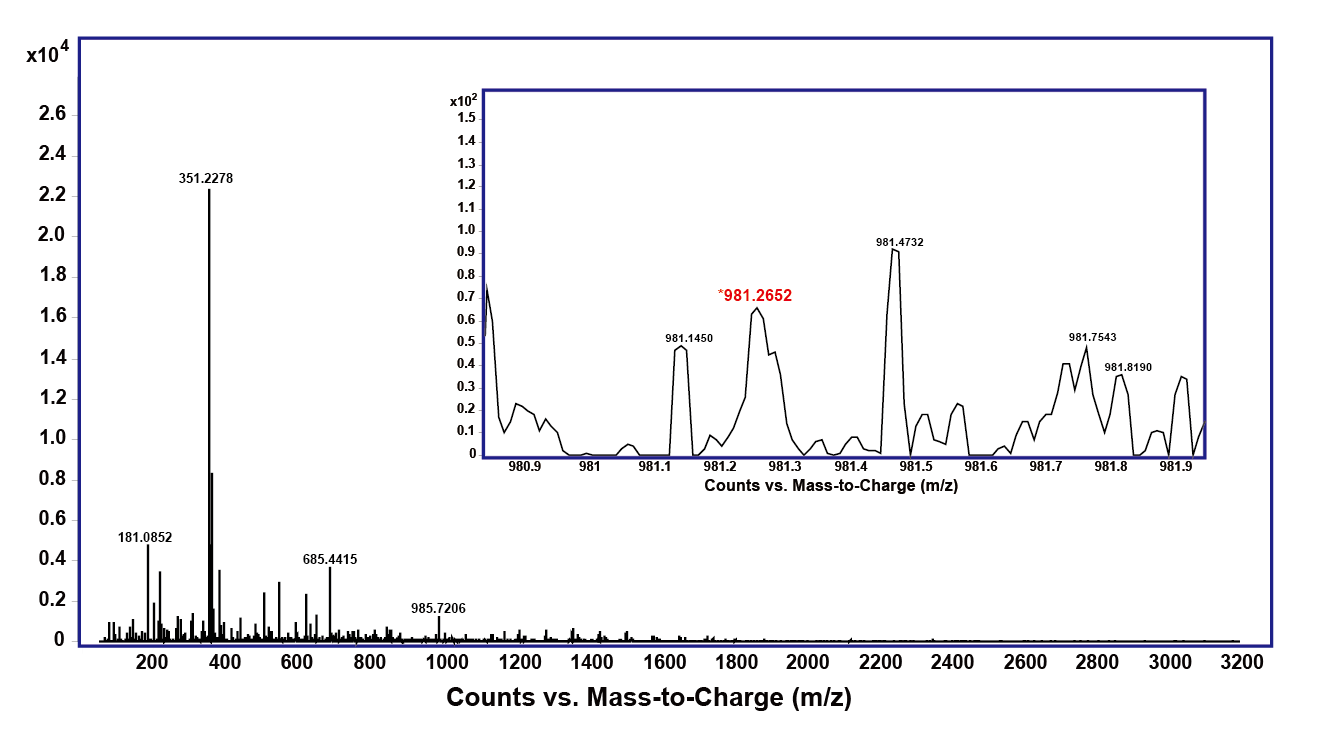


***Figure S4*** ESI-HRMS spectrum of **TPT-Se**.

***3. Preparation of DPT NPs, DDT NPs, HM and DDT-HM NPs***

***3. 1 Preparation of DPT NPs***

**TPT** (**TPT-S** or **TPT-Se**, 1 mg) was dissolved in THF solution (1 mL). Separately, DSPE-PEG_2000_ (10 mg) was dissolved in deionized water (10 mL). The **TPT**/THF solution was added dropwise to the vigorously stirred DSPE-PEG_2000_ aqueous solution under ultrasonication (36 W) to facilitate self-assembly. The resulting micellar dispersion was left undisturbed in a fume hood to evaporate THF. After complete evaporation of THF, the **DPT** nanoparticle (NPs) suspension was concentrated under reduced pressure at 4°C for subsequent use.

***3.2 Preparation of DDT NPs***

**TPT** (**TPT-S** or **TPT-Se**, 1 mg) and **DMXAA** (10 mg) was co-dissolved in THF (2 mL). DSPE-PEG_2000_ (15 mg) was dissolved in deionized water (10 mL). The combined **TPT**/**DMXAA**/THF solution were added dropwise to the vigorously stirred DSPE-PEG_2000_ aqueous solution under ultrasonication (36 W) to form co-assembled micelles. The resulting micellar dispersion was left undisturbed in a fume hood to evaporate THF. After complete evaporation of THF, the resulting **DDT** NP suspension was concentrated under reduced pressure at 4°C for subsequent use.

***3.3*** ***Preparation of HM***

4T1 and RAW264.7 cells were harvested separately by centrifugation and resuspended in ice-cold hypotonic lysis buffer containing protease inhibitors. After 12-min incubation on ice, cellular membranes were isolated through four rapid freeze-thaw cycles, alternating between liquid nitrogen immersion (2 min) and a 37°C water bath (2 min). The lysate was centrifuged at 700 × g for 10 min (4°C) to remove nuclei and cellular debris. The supernatant was then transferred to a new tube and ultracentrifuged at 14,000 × g for 30 min (4°C). The resulting membrane pellet was resuspended in PBS containing 1% phenylmethylsulfonyl fluoride (PMSF) to obtain the hybrid membrane (**HM**) protein suspension.

***3.4 Preparation of DDT-HM NPs***

The **HM** suspension was combined with **DDT** NPs and co-extruded through a 200 nm polycarbonate membrane using a mini-extruder at room temperature for a minimum of 10 passes. This process yielded the hybrid membrane-camouflaged NPs (**DDT-HM** NPs). The resulting **DDT-HM** NPs were purified by centrifugation (14,000 × g, 15 min, 4°C) to remove uncoated membrane fragments and stored in PBS at 4°C for further characterization and use.

**4. Characterization of DDT NPs**

***4.1 Absorption spectra***

UV-Vis absorption spectra of free **TPT-S** and **PT-Se** (dissolved in THF) and their corresponding encapsulated forms (**DPT-S** NPs, **DPT-Se** NPs, **DDT-S** NPs, **DDT-Se** NPs; dispersed in deionized water) were recorded using a Hitachi U-5300 spectrophotometer. Baseline corrections were performed using pure THF and pure water as reference solvents, respectively. Spectral shifts and changes in molar absorptivity upon NP formation were analyzed to confirm successful encapsulation and assess potential aggregation states.


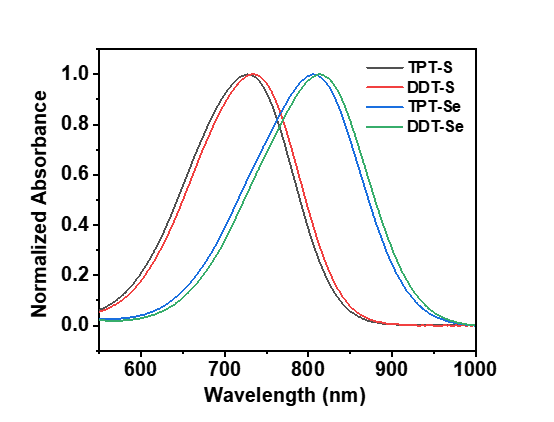


***Figure S5*** UV-vis absorption spectra of **TPT-S** and **TPT-Se** in THF versus **DDT-S** NPs and **DDT-Se** NPs in water.

***4.2 Drug loading efficiency***

The drug loading efficiency (LE%) of DMXAA within **DDT** NPs was determined spectrophotometrically. Calibration curves were established by measuring the absorbance at 808 nm (*λ*_max_ for **TPT-S** and **TPT-Se**) across a series of known concentrations of **TPT-S** or **TPT-Se** in THF. **DDT** NP suspensions were lysed using THF to release the encapsulated components, and the absorbance at 808 nm was measured. The mass of **TPT** in the NPs was calculated using the corresponding calibration curve. LE% was calculated using the formula:

LE% = (Mass of drug in **DDT NPs** / Total mass of **DDT NPs**) × 100%

The measured LE% was 3.12 ± 0.8% for **TPT-Se**, 2.18 ± 0.3% for **TPT-S**, and 12.50 ± 1.2% for DMXAA. The total mass of **DDT** NPs was determined by lyophilizing a known volume of the purified NP suspension and measuring the dry weight.


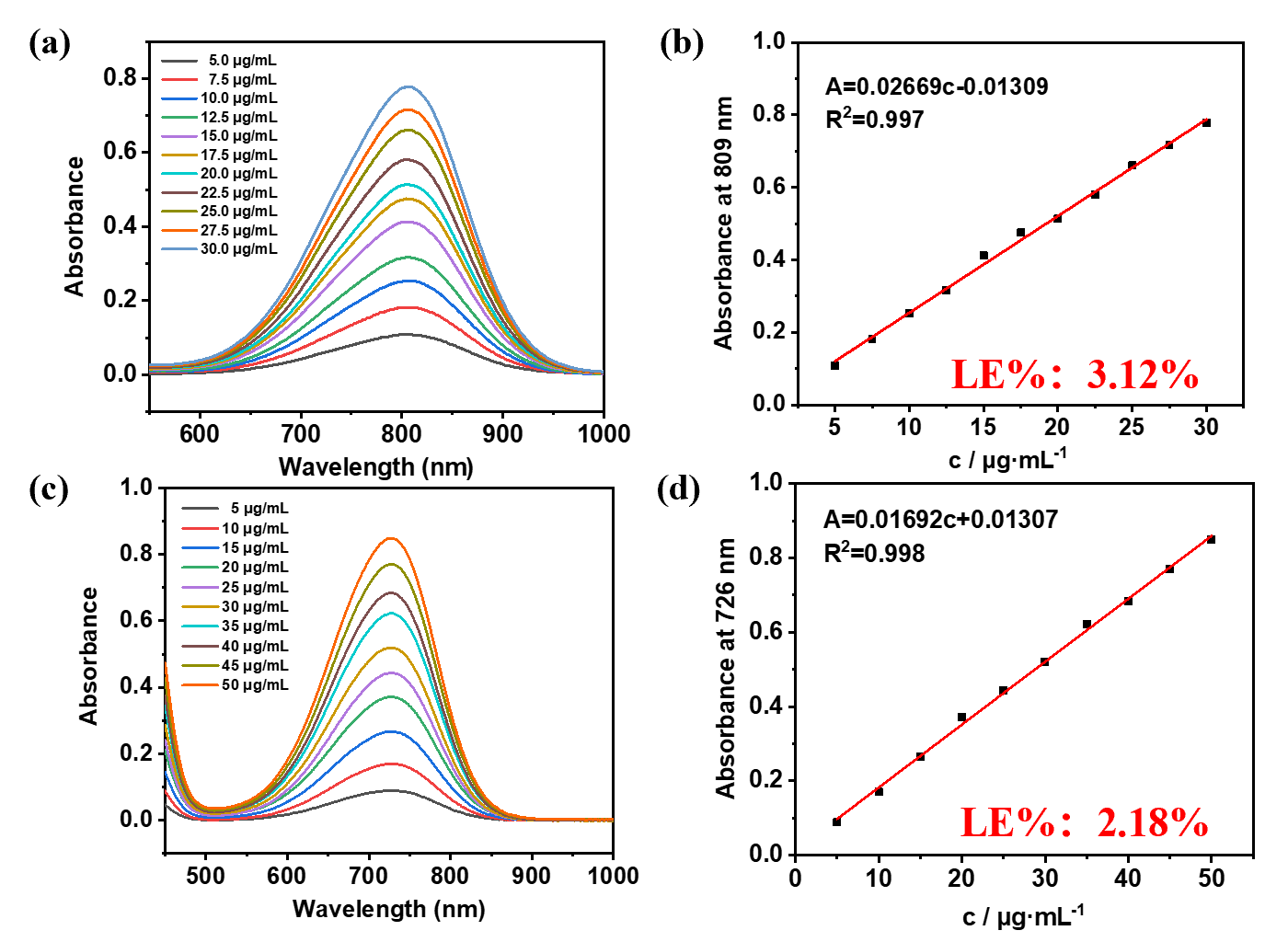


***Figure S6*** (a) UV-vis absorption spectra and (b) standard calibration curve of **TPT-Se** at different concentrations in THF; (c) UV-vis absorption spectra and (d) standard calibration curve of **TPT-S** at different concentrations in THF.

4.3 Morphology and size characterization

The morphology of **DDT** NPs was examined by TEM and AFM. Aqueous suspensions of **DDT** NPs were deposited onto carbon-coated copper grids and allowed to air-dry at room temperature prior to imaging on a Hitachi S-4800 microscope operating at 80 kV. Hydrodynamic diameter and size distribution were determined by DLS using a Malvern Zetasizer Nano ZS90. Measurements were performed at 25 °C using NP suspensions diluted in deionized water and filtered through 0.45 μm syringe filters immediately before analysis. Size distributions are reported as the average of three independent measurements.


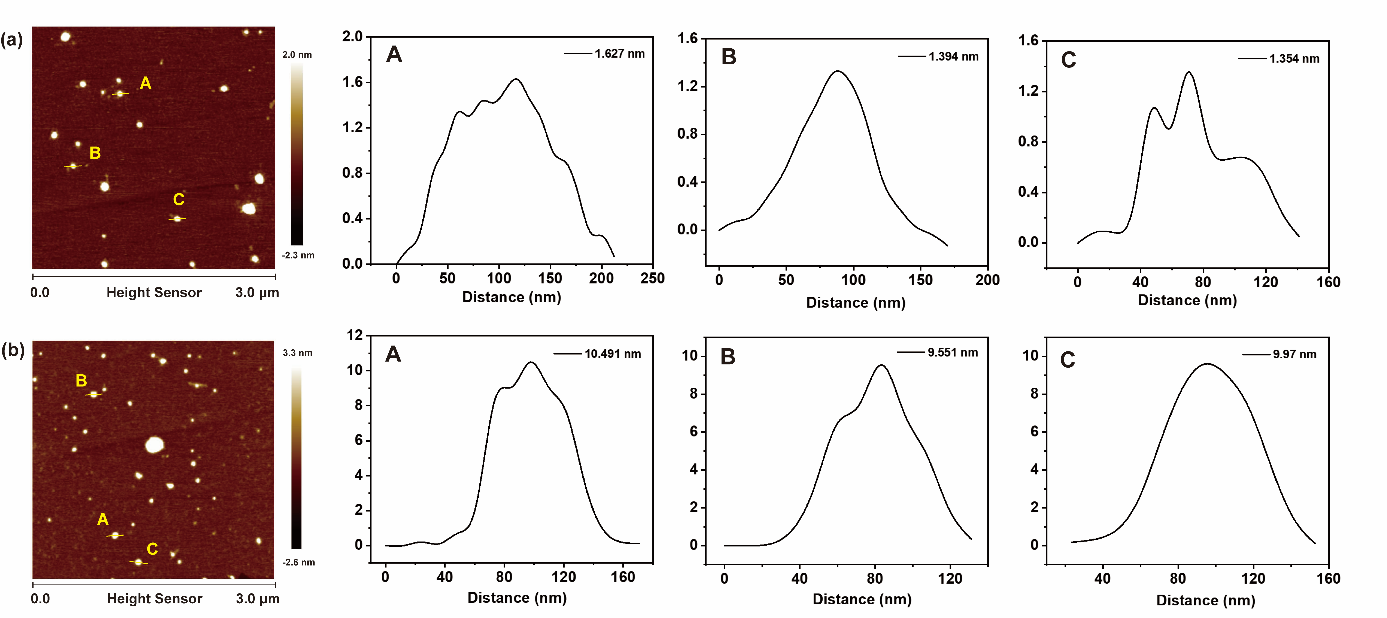


***Figure S7*** AFM images in air for (a) **DDT** NPs and (b) **DDT-HM** NPs.

***Figure S8*** Corresponding histograms for particle heights and diameters. In red for diameter and blue for height, showing significant statistical differences between the two nanoparticles.

4.4 Photophysical properties

4.4.1 Concentration-dependent photothermal performance

Aqueous suspensions of **DDT-S** NPs (20, 40, 60, 80 μg/mL) and **DDT-Se** NPs (5, 10, 15, 20, 25 μg/mL) in 1.5 mL tubes were irradiated with an 808 nm NIR laser (0.8 W/cm^2^) for 10 min. Temperature profiles were monitored at 1-min intervals using an FLIR ONEPro IR thermal camera. Deionized water served as a negative control.

4.4.2 Power density-dependent performance

Aqueous suspensions of **DDT-S** NPs (60 μg/mL) and **DDT-Se** NPs (10 μg/mL) were irradiated with an 808 nm laser at varying power densities (0.2, 0.4, 0.6, 0.8 and 1.0 W/cm^2^) for 10 min. Temperature evolution was performed in real-time using an FLIR thermal camera.

4.4.3 Photothermal stability

The photothermal stability of **DDT-S** NPs (60 μg/mL) and **DDT-Se** NPs (10 μg/mL) was evaluated by subjecting them to four consecutive laser ON/OFF cycles. Each cycle consisted of 10 min of irradiation (808 nm, 0.8 W/cm^2^) followed by 10 min of natural cooling to ambient temperature. Temperature evolution was recorded throughout the cycling process.

4.4.4 Photothermal conversion efficiency

The photothermal conversion efficiency (*η*) was calculated according to established methods. Briefly, aqueous suspensions of **DDT-S NPs** (60 μg/mL, 1.0 mL) and **DDT-Se NPs** (10 μg/mL, 1.0 mL) in sealed quartz cuvettes were irradiated with an 808 nm laser (0.8 W/cm^2^) until a steady-state maximum temperature (T_max_) was reached (typically within 10 min). The laser was then turned off, and the temperature decrease was recorded during natural cooling. The same procedure was performed with deionized water as a control. Temperature was recorded at 1 min intervals using an FLIR camera. Photothermal conversion efficiency was calculated as:

$$\eta=\frac{hS\left( T_{max}-T_{0} \right)-Q_{s}}{I(1-{10}^{-A})}\times100\%$$

Where *T*_max_ denotes the maximum temperature of the sample at the jarless state; *T*_0_ represents the initial temperature of the sample; *Q*_dis_ expresses the dissipation of heat; *I* is the laser power; A is the absorbance of the sample at 808 nm.

*hS* is obtained from the following equation:

ℎ𝑆 =$\frac{mDcD}{\tau s}$

m_D_ is the mass of the solution (1.0 g); c_D_ is the particular heat capacity of the solution; τ_s_ is a time constant of the solution which can be calculated by the following equation:

𝜏_𝑠_ = ‒ $\frac{t}{ln(\frac{T\mathrm{surr} ‒ T}{T\mathrm{surr} ‒ T\max})}$

*T*_surr_ is surrounding temperature; T is real-time temperature of the sample.

*Q*_dis_ is determined by the following equation:

𝑄_𝑑𝑖𝑠_ =$\frac{mDcD(T\max, water ‒ T\mathrm{surr})}{\tau\mathrm{water}}$

*T*_max, water_ is the maximum temperature of the water at the jarless state; τ_water_ is the time constant of the control group.


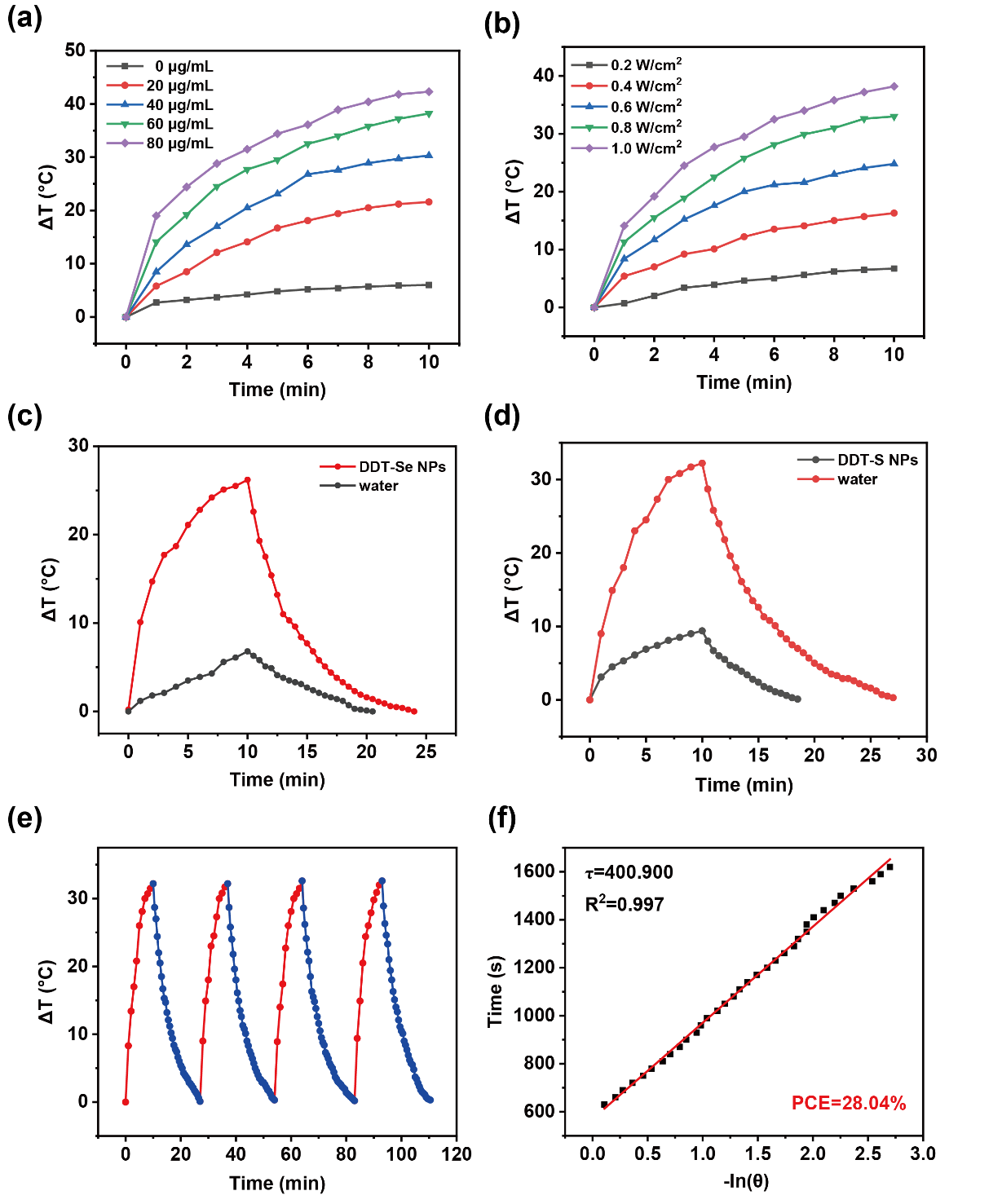


***Figure S9*** Photothermal characterization of **DDT** NPs. (a) Concentration-dependent temperature profiles of **DDT-S** NPs under 808 nm laser irradiation (0.8 W/cm^2^, 10 min). (b) Power density-dependent temperature evolution of **DDT-S** NPs (60 μg/mL); (c) Temperature evolution of **DDT-Se** NPs (10 μg/mL) during irradiation (808 nm, 0.8 W/cm^2^, 10 min) and subsequent cooling; (d) Temperature evolution of **DDT-S** NPs (60 μg/mL) during irradiation (808 nm, 0.8 W/cm^2^, 10 min) and subsequent cooling; (e) Photothermal heating curves of **DDT-S** NPs (60 μg/mL) under four laser ON/OFF cycles (808 nm, 0.8 W/cm^2^, 10 min ON/ cooling OFF); (f) Linear time data versus -ln(θ) derived from the cooling curve of **DDT-S** NPs (60 μg/mL) after laser irradiation, where θ = (T - T_surr_) / (T_max_ - T_surr_). The slope yields the time constant τ_s_.

4.4.5 Fluorescence quantum yield

The fluorescence quantum yield (Φ_F_) of **DDT NPs** was determined relative to the reference dye **IR-26** (Φ_F_ = 0.05% in 1,2-dichloroethane). Solutions of **DDT** NPs, **DDT-Se** NPs and **IR-26** were prepared at matched optical densities (OD) at the excitation wavelength (808 nm) within the linear absorbance range (OD < 0.1). Specifically, concentrations used were: **DDT-S** NPs: 8, 9, 10, 11, 12 μg/mL (OD_808nm_: 0.0648, 0.0822, 0.0888, 0.0982, 0.1068); **DDT-Se** NPs: 5, 6, 7, 8, 9 μg/mL (OD_808nm_: 0.1088, 0.1446, 0.1706, 0.1791, 0.1854); **IR-26**: 5, 6, 7, 8, 9 μg/mL in 1,2-dichloroethane. The integrated fluorescence intensity (Int) over the range 900–1500 nm was calculated for each sample. Integrated fluorescence intensity (Int) was plotted against absorbance (A) at 808 nm for both sample and reference. Φ_F_ was calculated using the formula:

$$\Phi_{\text{F, sample}}\text{=}\Phi_{\text{F, ref}}\text{×}\frac{\text{slope}_{\text{sample}}}{\text{slope}_{\text{ref}}}\text{×}\left( \frac{\text{η}_{\text{sample}}}{\text{η}_{\text{ref}}} \right)^{\text{2}}$$

Where slope is the linear fitting slope of the absorbance of the material with different concentrations and the corresponding area, *η* denotes the refractive index of the solvent, and Φ_F, ref_ is the quantum yield of **IR-26** in 1, 2-dichloroethane.


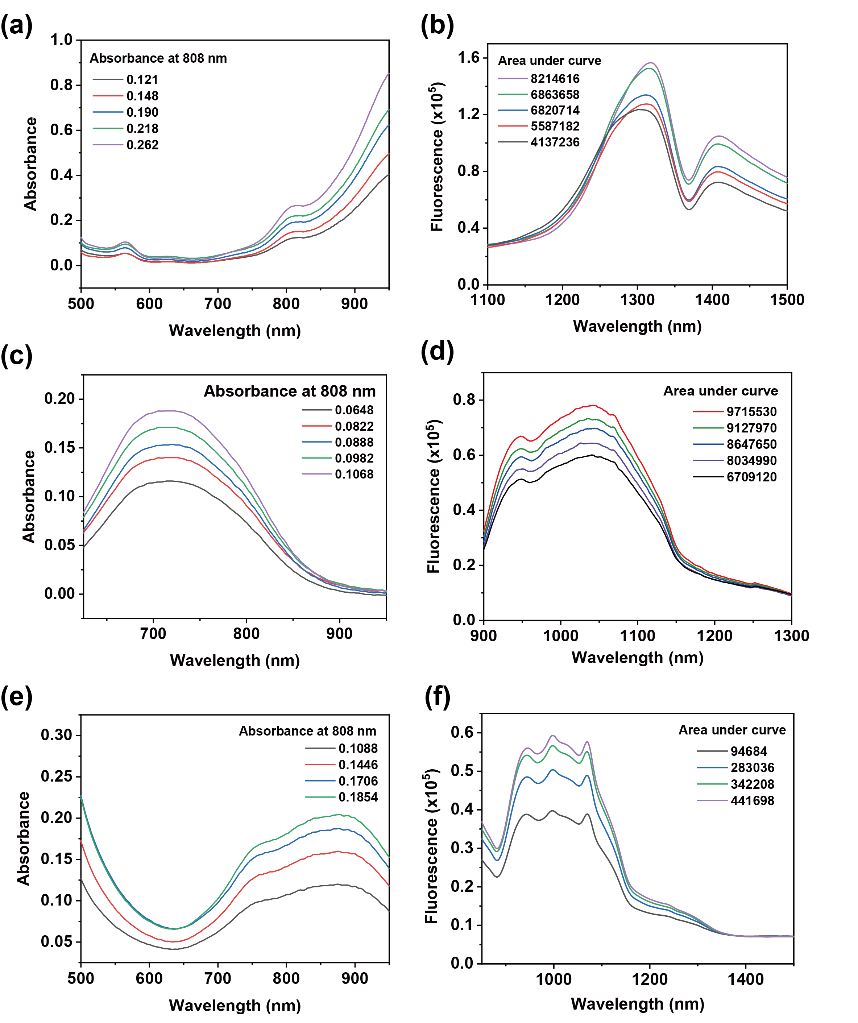


***Figure S10*** Spectroscopic data for quantum yield calculation. (a) UV-vis absorption spectra of **IR-26** at different concentrations in 1,2-dichloroethane; (b) Fluorescence emission spectra of **IR-26** at different concentrations in 1,2-dichloroethane (*λ*_ex_ = 808 nm); (c) UV-vis absorption spectra of **DDT-S** NPs at different concentrations in water; (d) Fluorescence emission spectra of **DDT-S** NPs at different concentrations in water (*λ*_ex_ = 808 nm); (e) UV-vis absorption spectra of **DDT-Se** NPs at different concentrations in water; (f) Fluorescence emission spectra of **DDT-Se** NPs at different concentrations in water (*λ*_ex_ = 808 nm).

**5. BCA protein assay**

Membrane protein solutions derived from 4T1 and RAW264.7 cells were aliquoted into 1.5 mL centrifuge tubes. Protein concentrations were quantified using a BCA assay kit according to the manufacturer's protocol. Briefly, a standard curve was generated in a 96-well plate by preparing bovine serum albumin (BSA) standards at concentrations of 0, 0.025, 0.05, 0.1, 0.2, 0.5, and 1.0 μg/μL. Membrane protein solutions were diluted 5-, 10-, and 20-fold in PBS. Standards and diluted samples (25 μL/well) were loaded into a 96-well plate, followed by addition of 200 μL BCA working reagent. and loaded into the plate. After gently agitation, the plate was incubated at 37 °C for 30 min. Absorbance was measured at 562 nm using a microplate reader, with blank wells (PBS + BCA reagent) serving as reference. Protein concentrations of the membrane solutions were interpolated from the standard curve, and results are expressed as mean ± SD (*n* = 3).

**6. Cell culture**

4T1, L929, U87, MCF-7 and RAW264.7 cells were cultured in DMEM supplemented with 10% FBS, 1% antibiotic/antimycotic solution (containing 50.0 IU mL^-1^ penicillin, 50.0 IU mL^-1^ streptomycin and 50.0 IU mL^-1^ amphotericin B). Cells were maintained at 37 ℃ in a humidified incubator with 5% CO_2_.

**7. *In vitro* characterization of HM**

7.1 Fusion verification of HM

To confirm successful **HM** formation, cancer cell membranes isolated from 4T1 cells were designated as the cancer cell membrane (**CM**), while those from RAW264.7 cells served as the macrophage membrane (**MM**). The thawed membrane protein solutions (37 °C water bath) were partially labeled with lipophilic fluorescent dyes: **MM** with DiO (green) and **CM** with DiD (red), incubated at 4°C in the dark. Hybrid membrane vesicles (**HMVs**) were then prepared by extruding **CM** and **MM** at a 1:1 membrane protein ratio using a liposome extruder. Successful membrane fusion was confirmed by laser scanning microscopy through the simultaneous detection of both DiO and DiD fluorescence signals in individual **HMVs**. Co-localization of DiD (*λ*_ex_/*λ*_em_ = 644/665 nm) and DiO (*λ*_ex_/*λ*_em_ = 484/501 nm) signals in individual vesicles was quantified using ImageJ (Pearson’s coefficient > 0.83).

7.2 Determination of optimal fusion ratio

The optimal **CM**:**MM** protein ratio for **HM** formation was determined using lineage-specific markers: **CD44** (cancer cell marker) and **CD206** (macrophage marker). Membrane proteins were incubated with anti-CD44-FITC (1:100) or anti-CD206-APC (1:100) antibodies at 4°C for 45 min. Labeled membranes were mixed at ratios of 1:0.5, 1:1, and 1:2 (**CM**:**MM**), extruded as in Section 7.1, and analyzed by flow cytometry. Marker retention was quantified as the percentage of dual-positive vesicles (CD44^+^/CD206^+^). The ratio yielding maximal dual-marker retention was identified as optimal for **HM** formation.

**8. *In vitro* characterization of DDT-HM *NPs***

8.1 Immune escape assessment

The immune evasion capability of hybrid membrane-coated NPs was evaluated using RAW264.7 macrophages. Cells were seeded in 12-well plates (2 × 10^5^ cells/well) and cultured for 18 h. The medium was replaced with serum-free DMEM containing PBS (control), **DDT-MM** NPs, **DDT-CM** NPs, or **DDT-HM N**Ps (0.2 μg/mL; **HM** protein ratio 4T1: RAW = 1: 1). After 4-h incubation, cells were washed three times with PBS, detached with 0.25% trypsin-EDTA, and resuspended in PBS containing 2% FBS. NP uptake was quantified by flow cytometry using the intrinsic NIR fluorescence of **TPT** (*λ*_ex_/*λ*_em_ = 808/980 nm). Data were analyzed using FlowJo v10.8, with results expressed as mean fluorescence intensity (MFI) ± SD (*n* = 3).

8.2 Homologous targeting evaluation

The cancer cell-specific targeting of **DDT-HM** NPs was assessed using L929, U87, MCF-7, RAW264.7, and 4T1 cell lines. Cells were seeded in 12-well plates (2 × 10^5^ cells/well) for 18 h. Medium was replaced with serum-free DMEM containing **DDT-HM** NPs (0.2 μg/mL) prepared at different membrane protein ratios (4T1:RAW264.7 = 1:1, 1:2, 1:3, 2:1, 3:1). Following 4 h incubation, cells were washed three times with PBS, detached, and analyzed by flow cytometry as in Section 8.1. Targeting specificity was quantified as the fold-change in MFI relative to L929 cells (non-cancer control).

***8.3 In vitro Cytotoxicity (MTT Assay)***

Cytotoxicity was evaluated using 4T1 cells seeded in 96-well plates (10^4^ cells/well). After 18 h, medium was replaced with fresh medium containing **DDT** NPs or **DDT-HM** NPs (0.05, 0.1, 0.15, 0.2, and 0.25 μg/mL). Post 4-h incubation, cells were irradiated (808 nm laser, 0.8 W/cm^2^, 3 min) or kept in darkness. Following 18-h further incubation, MTT solution (0.5 mg/mL) was added per well. After 4 h, medium was replaced with 150 μL DMSO to dissolve formazan crystals. Absorbance at 570 nm was measured using a microplate reader. Cell viability was calculated as:

$$\text{Cell viability }(\%)=\frac{\text{OD value of experimental group -OD value of blank control group }}{\text{OD value of negative control group -OD value of blank control group}}\times100\%$$

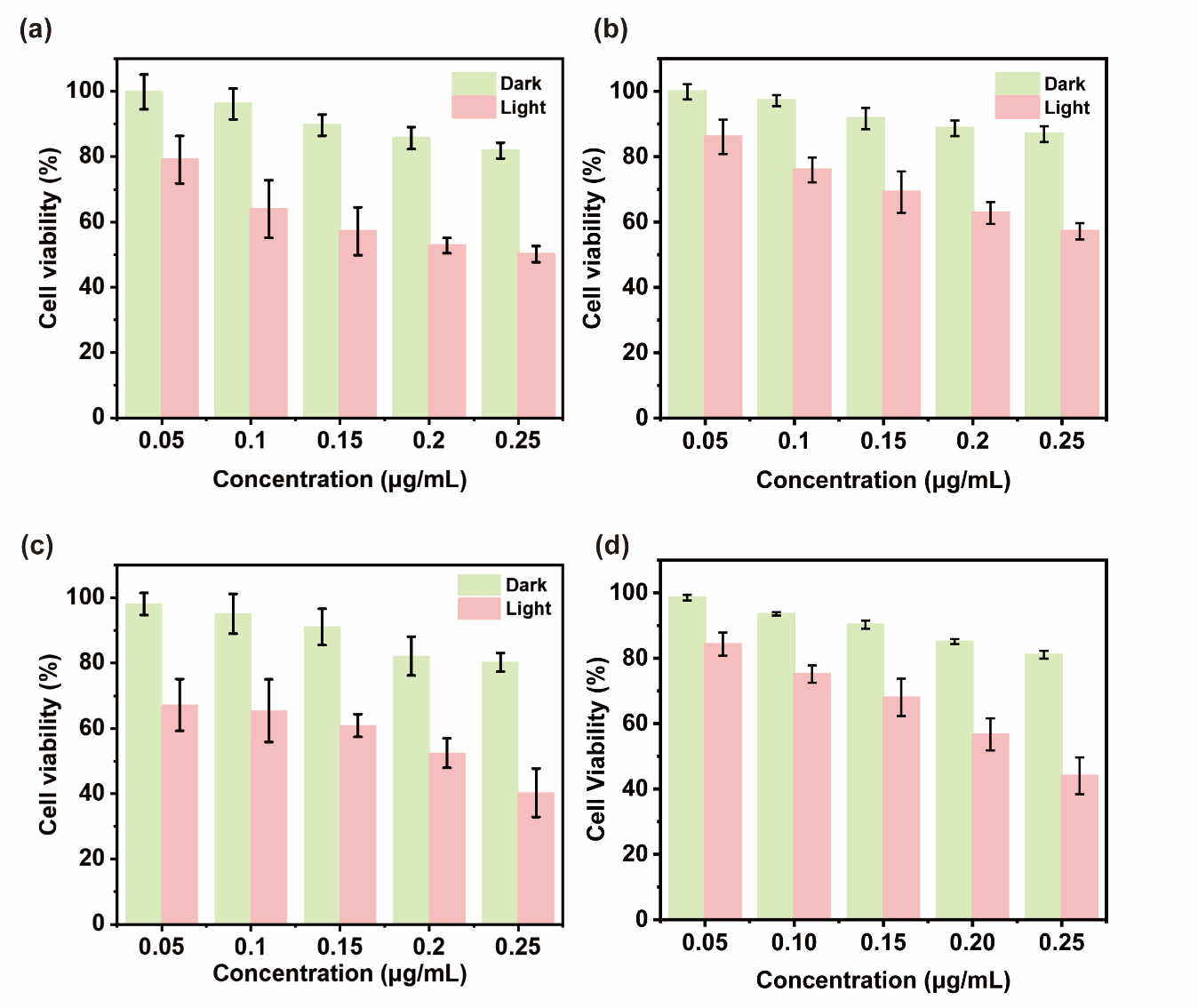


***Figure S11*** Viability of 4T1 cells treated with (a) **DDT**, (b) **DDT-MM**, (c) **DDT-CM** and (d) **DDT-HM** NPs (0.05–0.25 μg/mL) ± laser irradiation (808 nm, 0.8 W/cm^2^, 3 min). Data: mean ± SD (*n* = 5).

***8.4 Live/dead cell staining***

Membrane integrity post-treatment was visualized using calcein-AM/PI co-staining. 4T1 cells were seeded in 24-well plates (4 × 10^4^ cells/well) for 18 h. Medium was replaced with fresh DMEM containing PBS, **DDT** NPs (0.2 μg/mL), or **DDT-HM** NPs (0.2 μg/mL). After 4 h, designated wells received NIR irradiation (808 nm laser, 0.8 W/cm^2^, 3 min); controls remained unirradiated. Post 24 h incubation, cells were washed with PBS and co-stained with calcein-AM (2 μM) and propidium iodide (PI, 4.5 μM) for 15 min. After PBS washes, live (green, calcein) and dead (red, PI) cells were visualized by CLSM.

8.5 Cellular uptake analysis

To quantitatively compare the cellular uptake efficiency of the membrane-camouflaged NPs by macrophages, RAW264.7 cells were seeded in 8-well chamber slides (1.5 ×1 0^4^ cells/well) for 24 h. Medium was replaced with fresh DMEM containing PBS, **DDT** NPs (0.2 μg/mL), or **DDT-HM** NPs (0.2 μg/mL) after 18 h incubation. After another 4-h incubation, cells were washed twice with PBS, detached using enzyme-free dissociation buffer, washed once more with PBS, and resuspended in PBS containing 2% FBS for flow cytometric analysis.

**9. *In vitro* assessment of Immunogenic cell death (ICD)**

***9.1 Surface expression of CRT***

4T1 cells were seeded in 24-well plates (5 × 10^4^ cells/well) for 18 h. Cells were treated with free DMEM, **DDT** NPs, or **DDT-HM** NPs (0.2 μg/mL) for 4 h, followed by irradiation with an 808 nm laser (0.8 W/cm^2^, 3 min) or no irradiation. After 18 h incubation, cells were fixed with 4% paraformaldehyde (15 min), permeabilized with 0.1% Triton X-100 (10 min), blocked with 5% BSA (30 min), and incubated with anti-CRT primary antibody (1:200, 1 h) followed by Alexa Fluor 488-conjugated secondary antibody (1:500, 45 min). Nuclei were counterstained with DAPI (1 μg/mL, 5 min). Imaging was performed using an epifluorescence microscope. Non-fixed cells were incubated with anti-CRT antibody (30 min, 4°C), washed, stained with Alexa Fluor 488-conjugated secondary antibody (30 min, dark), and analyzed by flow cytometry.

***9.2 Extracellular Translocation of HMGB1***

4T1 cells were seeded in a 24-well plates (5 × 10^4^ cells/well) for 18 h and treated with DMEM (control), **DDT** NPs, or **DDT-HM** NPs (0.2 μg/mL). After 4 h incubation. cells were either irradiated (808 nm laser, 0.8 W/cm^2^, 3 min) or kept in darkness, followed by 18 h incubation. Cells were fixed (4 % paraformaldehyde, 20 min), permeabilized (0.1 % Triton X-100, 10 min), and blocked (5% FBS in PBS, 30 min). Samples were incubated with anti-HMGB1 antibody (1:200, 1 h, RT) and Alexa Fluor 488-conjugated secondary antibody (1:500, 30 min, dark). Nuclei were counterstained with DAPI (1 μg/mL, 5 min). Imaging was performed using an epifluorescence microscope. Nuclear-to-cytoplasmic HMGB1 translocation was quantified using ImageJ (*n* = 50 cells).

Cell supernatants were collected for extracellular HMGB1 analysis. Adherent cells were detached using enzyme-free dissociation buffer, washed with PBS, and blocked (5% FBS, 30 min). After incubation with anti-HMGB1 antibody (1 h, 4°C) and Alexa Fluor 488-conjugated secondary antibody (30 min, 4°C, dark), cells were resuspended in PBS for FACS analysis.

***9.3 ATP secretion quantification***

Extracellular ATP release, a hallmark of (ICD, was quantified using a bioluminescence assay. 4T1 cells were seeded in a 24-well plates (5 × 10^4^ cells/well) for 18 h and treated with DMEM (control), **DDT** NPs, or **DDT-HM** NPs (0.2 μg/mL). After 4 h incubation. cells were either irradiated (808 nm laser, 0.8 W/cm^2^, 3 min) or kept in darkness. After 18-h incubation, cell culture supernatants were collected and centrifuged (300 × g, 5 min). Extracellular ATP levels were quantified using an ATP Bioluminescence Assay Kit according to manufacturer protocols. Luminescence was measured with a microplate reader and normalized to total cellular protein content.

***Figure S12*** ATP release by 4T1 cells under different treatment conditions.

***10.*** ***Parallel study of ICD and STING pathway in vitro***

***10 .1 Surface expression of CRT***

4T1 cells were seeded in 24-well plates (5 × 10^4^ cells/well) for 18 h. Cells were treated with free DMEM, **DPT** NPs, or **DMXAA** NPs (0.2 μg/mL) for 4 h, followed by irradiation with an 808 nm laser (0.8 W/cm^2^, 3 min) or no irradiation. After 18 h incubation, cells were incubated with anti-CRT antibody (30 min, 4°C), washed, stained with Alexa Fluor 488-conjugated secondary antibody (30 min, dark), and analyzed by flow cytometry.

***10.2 Extracellular Translocation of HMGB1***

4T1 cells were seeded in a 24-well plates (5 × 10^4^ cells/well) for 18 h and treated with DMEM (control), **DPT** NPs, or **DMXAA** NPs (0.2 μg/mL). After 4 h incubation, cells were either irradiated (808 nm laser, 0.8 W/cm^2^, 3 min) or kept in darkness, followed by 18 h incubation. Adherent cells were detached using enzyme-free dissociation buffer, washed with PBS, and blocked (5% FBS, 30 min). After incubation with anti-HMGB1 antibody (1 h, 4°C) and Alexa Fluor 488-conjugated secondary antibody (30 min, 4°C, dark), cells were resuspended in PBS for FACS analysis.


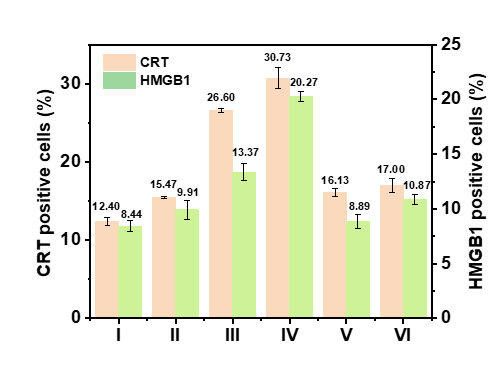


***Figure S13*** The positive cell rate of CRT and HMGB1 under different treatments. I: PBS, II: PBS + L, III: **DPT**, IV: **DPT** + L, V: DMXAA, VI: DMXAA + L.

***10.3 In vitro STING pathway activation analysis***

Extracellular IFN-β release was treated with DMEM (control), DPT NPs or DMXAA NPs (0.2 μg/mL) and quantified by STING using a bioluminescence assay. 4T1 cells were seeded in 24-well plates (5×10^4^ cells/well) for 18 h. After 4 hours of incubation, the cells were irradiated (808 nm laser, 0.8 W/cm^2^, 3 min) or kept in the dark. After incubation for 18 hours, the cell culture supernatant was collected and centrifuged (300×g, 5 min). Extracellular IFN-β levels were quantified using a human IFN-β ELISA kit according to the manufacturer 's protocol, and the luminescence values were measured using a microplate reader.


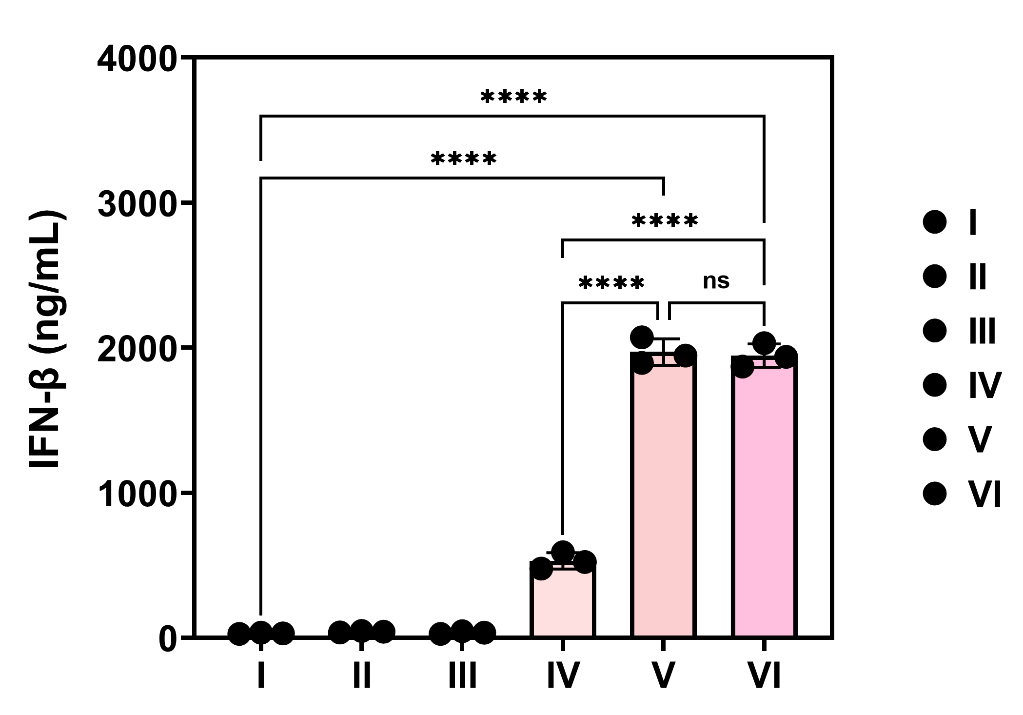


***Figure S14*** The expression of cytokine IFN-β in cell culture supernatant. I: PBS, II: PBS + L, III: **DPT**, IV: **DPT** + L, V: DMXAA, VI: DMXAA + L.

***11. DNA damage detection***

To evaluate the induction of DSBs, a hallmark of genotoxic stress and ICD, γ-H2AX foci formation was assessed in 4T1 cells. 4T1 cells were maintained in DMEM supplemented with 10 % FBS and 1% penicillin (50.0 IU mL^-1^), streptomycin (50.0 IU mL^-1^) and amphotericin B (50.0 IU mL^-1^) at 37 °C, 5% CO_2_. Cells were seeded in 12-well plates (3 × 10^5^ cells/well) for 18 h. Treatments were administered in serum-free DMEM containing: PBS (control), **DDT** NPs and **DDT-HM** NPs (0.2 μg/mL). After 4-h incubation, duplicate plates were either irradiated (808 nm NIR laser, 0.8 W/cm^2^, 3 min) or kept in darkness. Following 18-h incubation, all wells were replaced with fresh serum-free DMEM for an additional 18 h. Cells were then fixed and stained for γ-H2AX foci using a commercial DNA Damage Detection Kit according to manufacturer protocols. DNA double-strand breaks were visualized by epifluorescence microscopy, with foci quantification performed using ImageJ software.

***12. Mitochondrial membrane potential assessment***

Mitochondrial depolarization, an early indicator of apoptosis and cellular stress, was assessed using the potentiometric dye JC-1. 4T1 cells were cultured in DMEM supplemented with 10 % FBS and 1% penicillin (50.0 IU mL^-1^), streptomycin (50.0 IU mL^-1^) and amphotericin B (50.0 IU mL^-1^) at 37°C, 5% CO_2_. After seeding in 12-well plates at 3 × 10^5^ cells/well for 18 h, cells were treated with: PBS (control), **DDT** NPs and **DDT-HM** NPs (0.2 μg/mL) in serum-free DMEM. Post 4-h incubation, duplicate plates underwent either NIR irradiation (808 nm, 0.8 W/cm², 3 min) or no treatment. After 18 h, medium was replaced with fresh serum-free DMEM for a further 18 h. Cells were then washed gently with PBS and incubated with JC-1 staining solution for 30 min at 37 °C in the dark. After incubation, cells were washed twice with warm JC-1 staining buffer (or PBS) to remove excess dye. Cells were immediately visualized and imaged using a fluorescence microscope. JC-1 exhibits potential-dependent accumulation in mitochondria. In healthy cells with high Δψ_m_, JC-1 forms aggregates (*J*-aggregates) emitting red fluorescence (~590 nm emission when excited at ~550 nm). In cells with depolarized mitochondria (low Δψ_m_), JC-1 remains in the monomeric form, emitting green fluorescence (~530 nm emission when excited at ~485 nm).

**
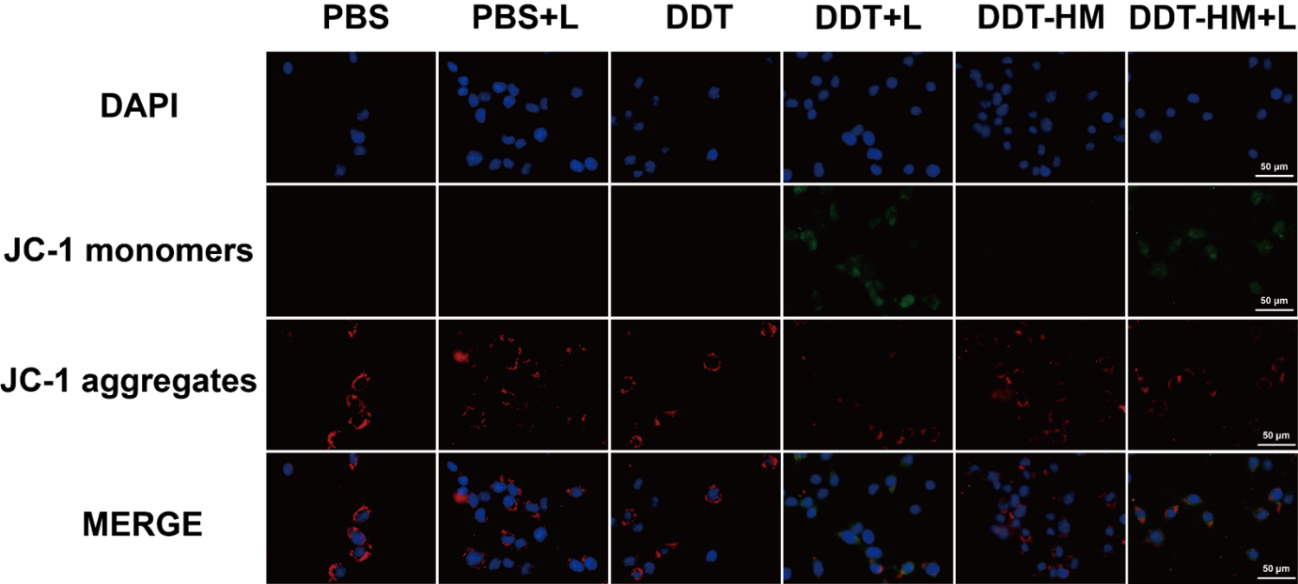
**

***Figure S15*** JC-1 fluorescence in 4T1 cells showing mitochondrial depolarization. Red fluorescence indicates *J*-aggregates (polarized mitochondria, Δψ_m_ high) while green fluorescence indicates JC-1 monomers (depolarized mitochondria, Δψ_m_ low). Treatments: PBS control, PBS + L, **DDT** NPs, **DDT** NPs + L, **DDT-HM** NPs, **DDT-HM** NPs + L (808 nm laser, 0.8 W/cm², 3 min).

***13.*** ***In vitro STING pathway activation analysis***

4T1 cells were maintained in DMEM supplemented with 10 % FBS and 1% penicillin (50.0 IU mL^-1^), streptomycin (50.0 IU mL^-1^) and amphotericin B (50.0 IU mL^-1^) at 37 °C, 5% CO_2_. Cells were seeded in 6-well plates (3 × 10^5^ cells/well) for 18 h and treated in serum-free DMEM containing PBS (control), **DDT** NPs and **DDT-HM** NPs. After 4-h incubation, duplicate plates were either irradiated (808 nm NIR laser, 0.8 W/cm^2^, 3 min) or kept in darkness. Following 18-h incubation, medium was replaced with fresh serum-free DMEM for 48 h to enable cytokine production. Cells were then washed three times with ice-cold PBS, scraped, and pelleted by centrifugation (16,000 × g, 20 min, 4 °C). Protein extracts were prepared using RIPA buffer supplemented with protease/phosphatase inhibitors. STING pathway activation was assessed by Western blotting for phospho-STING, TBK1, and IRF3.


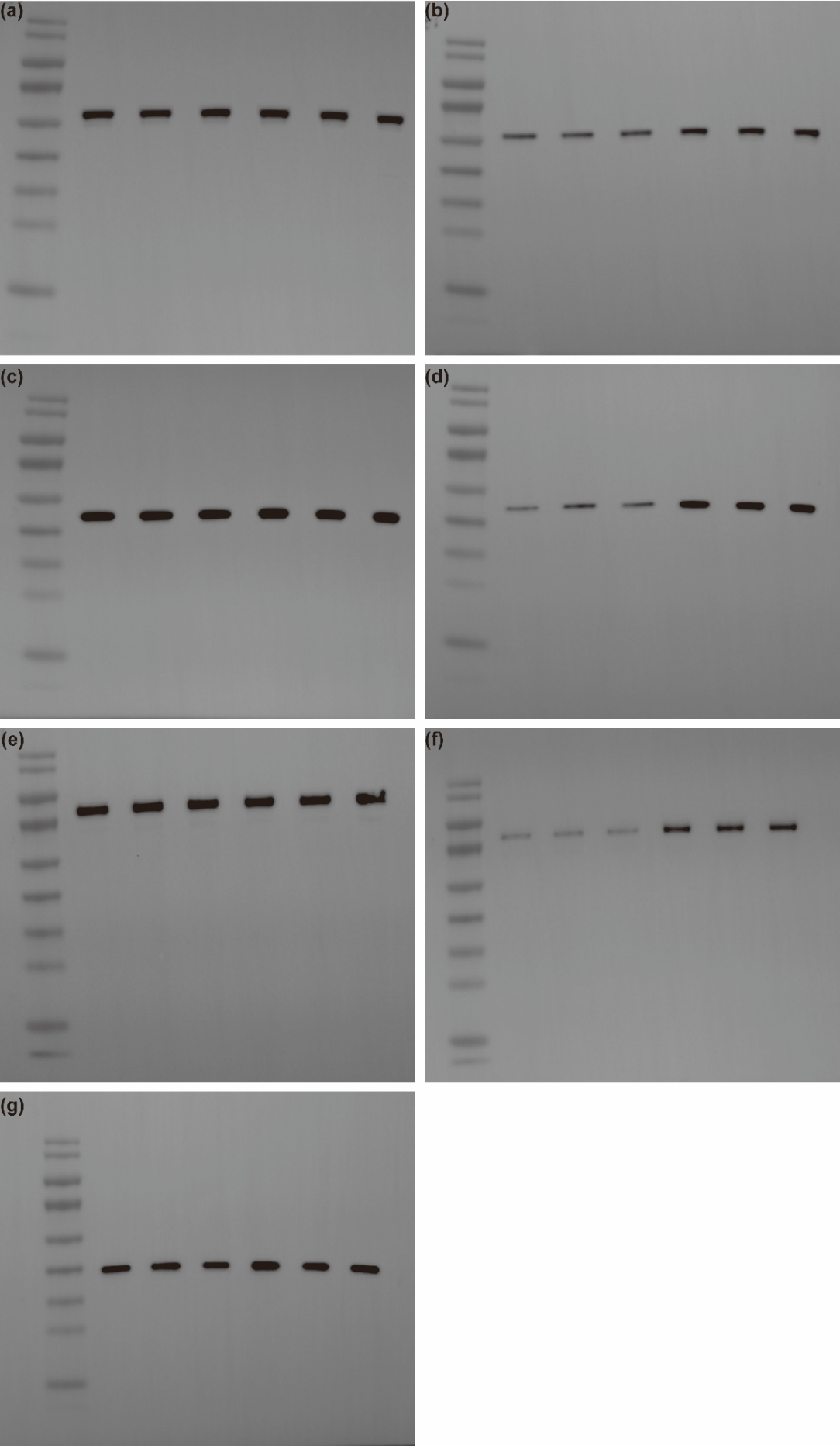


***Figure S16*** Western blot analysis of STING pathway proteins (a: IRF3, b: p-IRF3, c: STING, d: p-STING, e: TBK1, f: p-TBK1) 24 h post-treatment. g: GAPDH as control. The order from left to right is PBS, PBS + L, DDT, DDT + L, DDT-HM, DDT-HM + L.


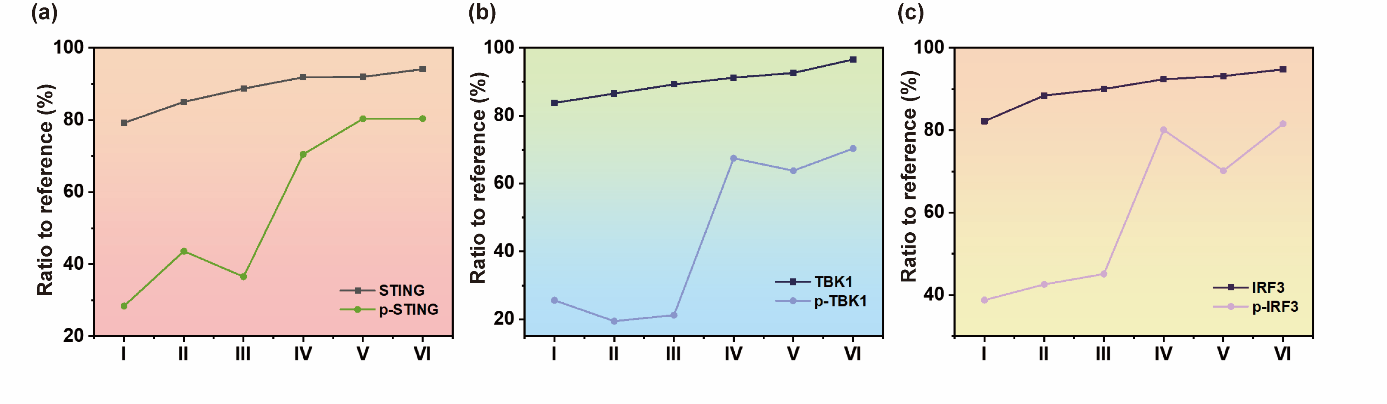


***Figure S17*** Relative protein expression analysis. Grayscale intensities normalized to GAPDH. I: PBS, II: PBS + L, III: DDT, IV: DDT + L, V: DDT-HM, VI: DDT-HM + L.

Extracellular IFN-β release was treated with DMEM (control), DDT NPs or DDT-HM NPs (0.2 μg/mL) and quantified by STING using a bioluminescence assay. 4T1 cells were seeded in 24-well plates (5×10^4^ cells/well) for 18 h. After 4 hours of incubation, the cells were irradiated (808 nm laser, 0.8 W/cm^2^, 3 min) or kept in the dark. After incubation for 18 hours, the cell culture supernatant was collected and centrifuged (300×g, 5 min). Extracellular IFN-β levels were quantified using a human IFN-β ELISA kit according to the manufacturer 's protocol, and the luminescence values were measured using a microplate reader.


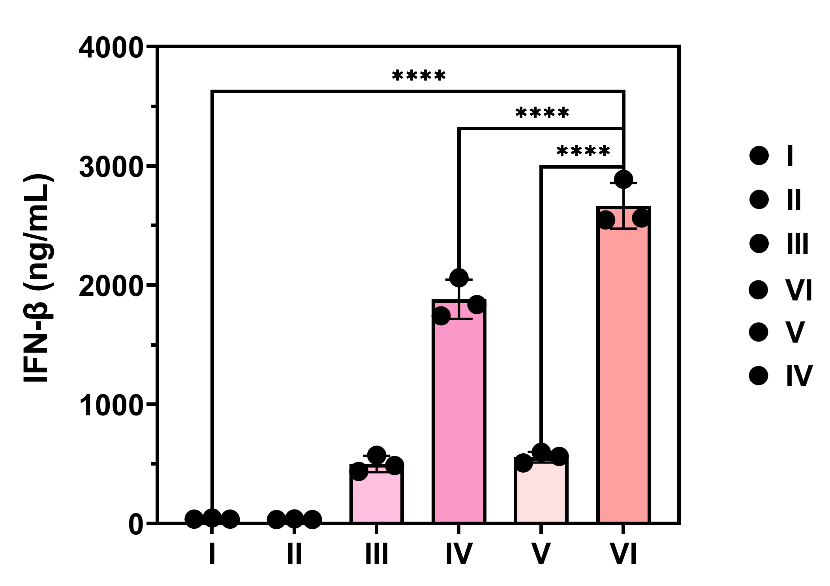


***Figure S18*** The expression of cytokine IFN-β in cell culture supernatant. I: PBS, II: PBS + L, III: DDT, IV: DDT + L, V: DDT-HM, VI: DDT-HM + L.

***14. Animals models and tumor establishment***

Female BALB/c nude mice (4-6 weeks old, 16-18 g) were purchased from Charles River Laboratory China Branch (Zhejiang, China; production license: SCXK [Zhejiang] 2019-0001). Mice were housed in a pathogen-free environment (controlled temperature and humidity) at the Laboratory Animal Center of Hangzhou Normal University (use license: SYXK [Zhejiang] 2020-0026). All animal procedures were complied with the guidelines of the Institutional Animal Care and Use Committee of Hangzhou Normal University. A bilateral 4T1 tumor-bearing model was stablished by subcutaneously injecting 100 μL of cell suspension (1 × 10^6^ 4T1 cells) into the right dorsal flank to generate the primary tumor. Six days later, an identical number of cells were injected into the left abdominal flank to simulate a distant metastatic tumor. Primary tumors were allowed to grow for 8 days before biodistribution and therapy studies commenced.

**15. *In vivo* fluorescence imaging**

To non-invasively evaluate the biodistribution and tumor-targeting efficacy of the hybrid membrane-camouflaged NPs, in vivo NIR fluorescence imaging was performed on bilateral 4T1 tumor-bearing BALB/c nude mice. Each mouse received an intravenous injection of 100 µL of PBS containing **DDT-HM NPs** (5 µg/mL). At designated time points (2, 4, 8, 12, and 24 h) post-injection, fluorescence imaging was performed using an IVIS system. Fluorescence intensity in the primary tumor was quantified per mouse using Living Image software. At 72 h post-injection, mice were euthanized. Primary tumors, hearts, livers, spleens, lungs, and kidneys were harvested for *ex vivo* fluorescence imaging via IVIS, with subsequent quantification using Living Image software.


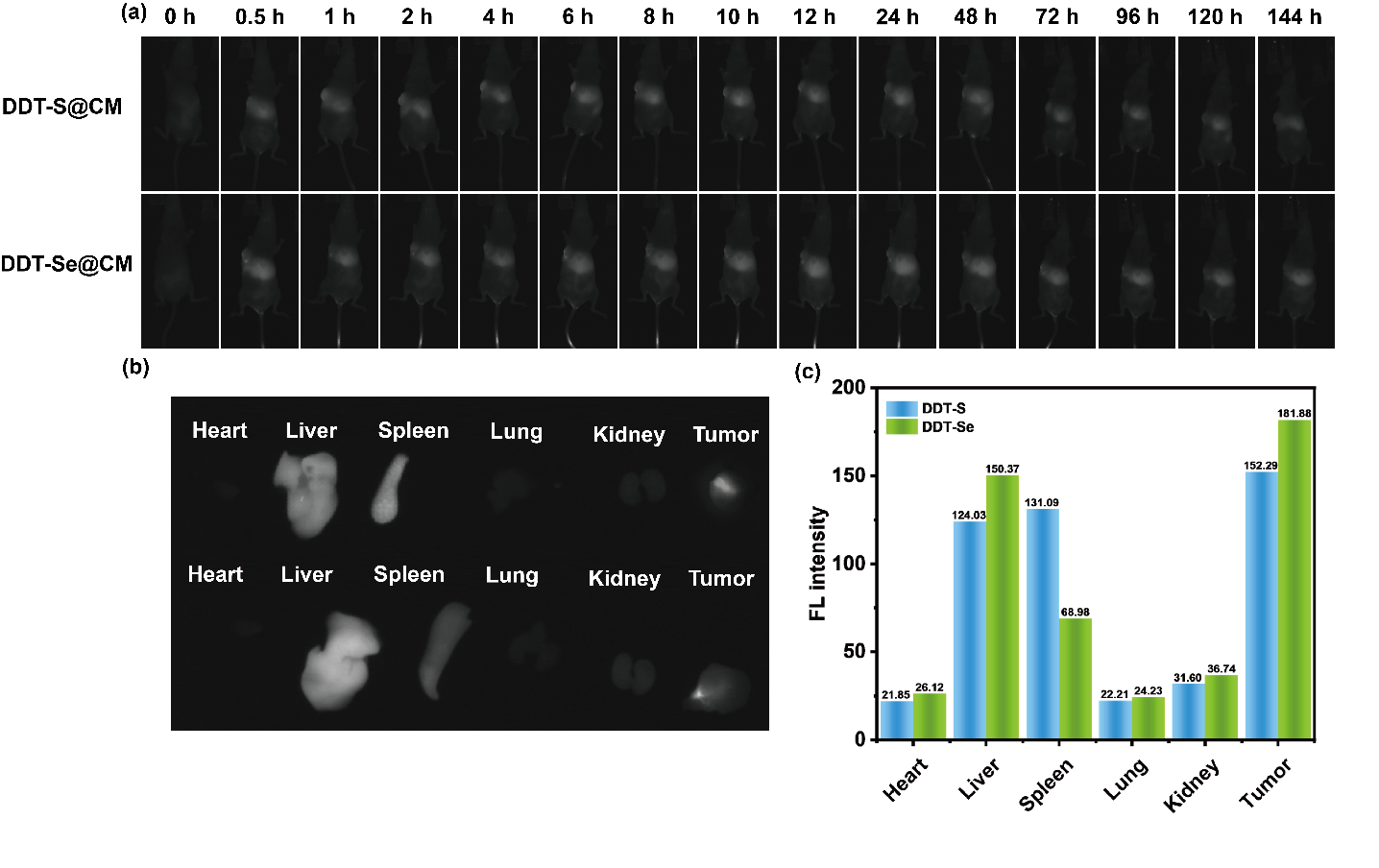


***Figure S19*** (a) NIR fluorescence imaging of subcutaneous 4T1 tumor-bearing mice at different time points after intravenous injection of **DDT-S@CM** and **DDT-Se@CM**. (b) Ex vivo fluorescence imaging of primary tumors and major organs (heart, liver, spleen, lung, kidney) 144 h post-injection. (c) Quantified fluorescence intensities of tumors and organs from (b).

***16. In vivo tumor thermal imaging and temperature monitoring***

The in vivo photothermal conversion capability of the NPs under NIR laser irradiation was evaluated in the 4T1 primary tumor model. 4T1 tumor-bearing mice were randomly assigned into three treatment groups (*n* = 6/group). Each group received subcutaneous injections of PBS (100 µL) containing PBS (control), **DDT** NPs, or **DDT-HM** NPs (5 µg/mL). Immediately following injection, primary tumors were irradiated with an 808 nm NIR laser (0.8 W/cm^2^) for 10 min. The temperature evolution within the tumor region was monitored in real-time throughout the irradiation period using a calibrated FLIR ONE Pro infrared thermal camera positioned perpendicularly to the tumor surface. Thermal images were captured at 1-minute intervals. The maximum surface temperature reached within the tumor ROI and the temperature change (ΔT) were recorded for each mouse.

***17. In vivo antitumor efficacy evaluation***

To comprehensively assess the in vivo antitumor efficacy and systemic immune activation potential of the nanoplatforms, bilateral 4T1 tumor-bearing BALB/c nude mice were randomly assigned to six treatment groups (*n* = 8/group). Eight days after primary tumor implantation, groups received intravenous injections of: PBS (100 µL), PBS (100 µL) + L, **DDT** NPs (5 µg/mL in 100 µL PBS), **DDT** NPs (5 µg/mL in 100 µL PBS) + L, **DDT-HM** NPs (5 µg/mL in 100 µL PBS), and **DDT-HM** NPs (5 µg/mL in 100 µL PBS) + L. At 4-h post-injection, tumors in laser-treated groups (2, 4, 6) were irradiated with an 808 nm laser (0.8 W/cm^2^, 5 min). Primary and distant tumor volumes were monitored every 2 d for 24 d, calculated as: Volume = (length) × (width)^2^/2. Body weights were recorded concurrently as an indicator of systemic toxicity.

***17.1 Immunohistochemical analysis***

Primary tumors from each treatment group were harvested for histopathology. Tissues were immediately fixed in 4 % paraformaldehyde to preserve cellular architecture. Following fixation, samples were: 1) embedded in paraffin blocks; 2) sectioned (4-5 μm thickness); 3) deparaffinized in xylene (3 × 10 min); 4) rehydrated through graded ethanol series (100% → 95% → 85% → 70%; 5 min per step); 5) washed in PBS (3 × 5 min). Sections were then stained with hematoxylin and eosin (H&E), or processed for Ki67 immunohistochemistry and TUNEL apoptosis assays.


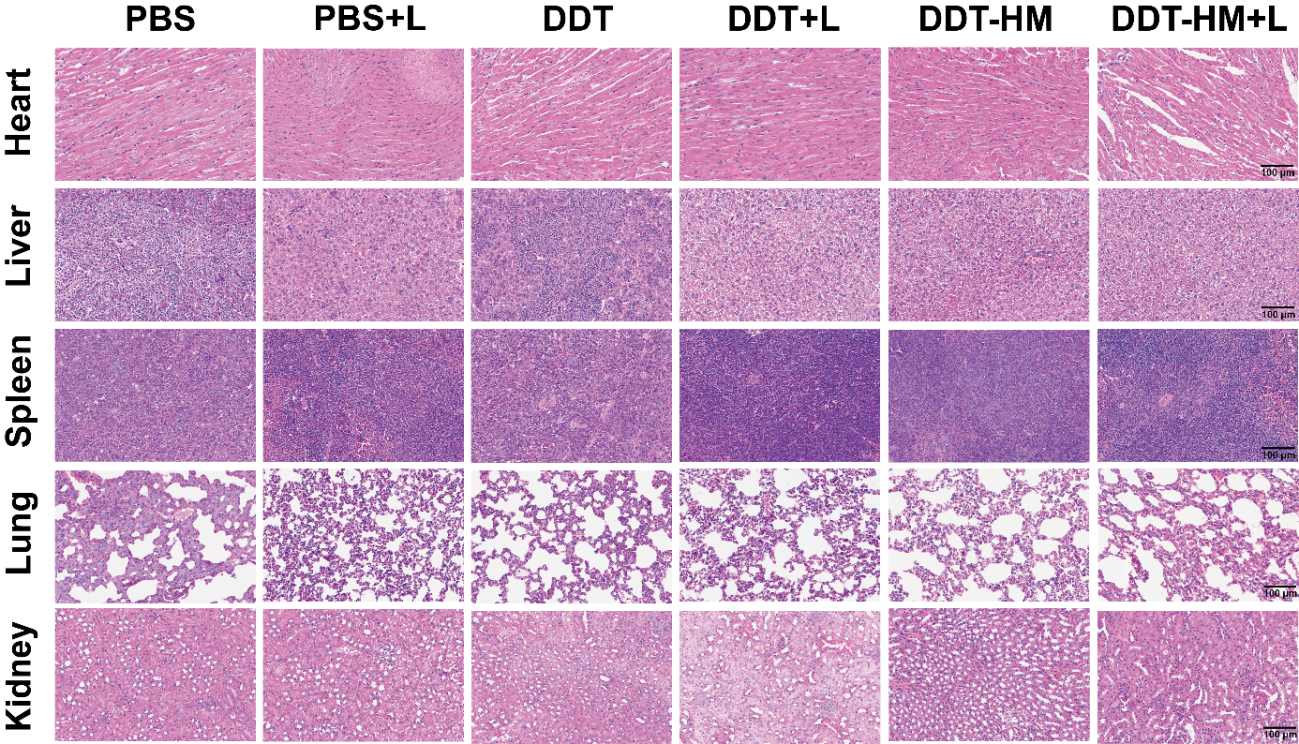


***Figure S20*** H&E staining of major organs (heart, liver, spleen, lung, kidney) of mice post-treatments (scale bar = 100 μm).

***17.******2 In vivo biosafety assessment***

Blood samples were collected for complete blood count and biochemical analysis, including: alkaline phosphatase (ALP), albumin (ALB), red blood cell count (RBC), hemoglobin count (HGB), mean platelet volume (MPV), hematocrit (HCT), mean corpuscular hemoglobin (MCH), mean corpuscular volume (MCV), red blood cell distribution width (RDW-SD), etc. No significant deviations from physiological ranges were detected in any group, indicating preserved hepatic/renal function and hematological homeostasis.


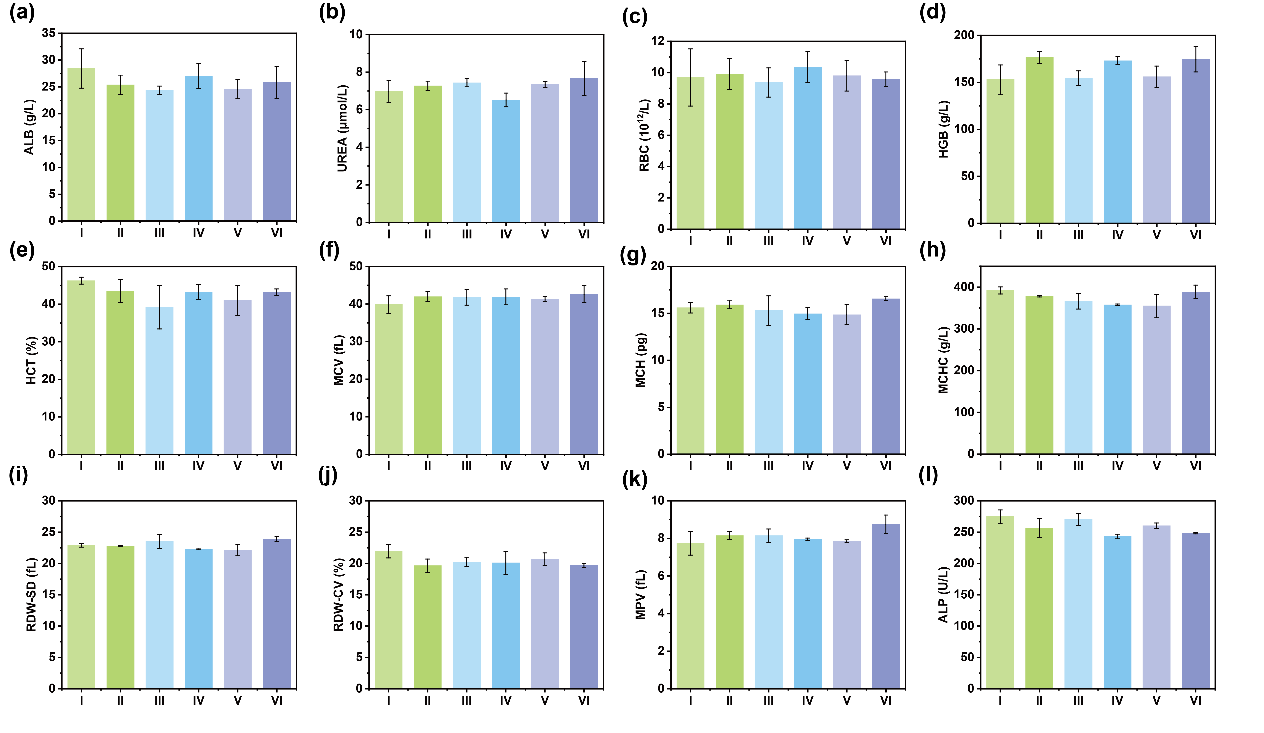


***Figure S21*** Hematological and biochemical profiles post-treatments (I: PBS; II: PBS + L; III: **DDT** NPs; IV: **DDT** NPs + L; V: **DDT-HM** NPs; VI: **DDT-HM** NPs + L).

***17.3 In vivo evaluation of DC maturation and macrophage phenotypes***

Following treatment completion, 4T1 tumor-bearing mice were euthanized. Primary tumors were dissociated through enzymatic digestion (1 mg/mL collagenase IV, 37°C, 4 h). Single-cell suspensions were prepared by sequential filtration through a 200-μm nylon mesh, followed by erythrocyte lysis. Cells were stained with fluorophore-conjugated antibodies: anti-CD11c-FITC, anti-CD86-PE, and anti-CD80-APC. After 30-min incubation, DC maturation (CD11c^+^, CD80^+^, CD86^+^) was analyzed by flow cytometry.


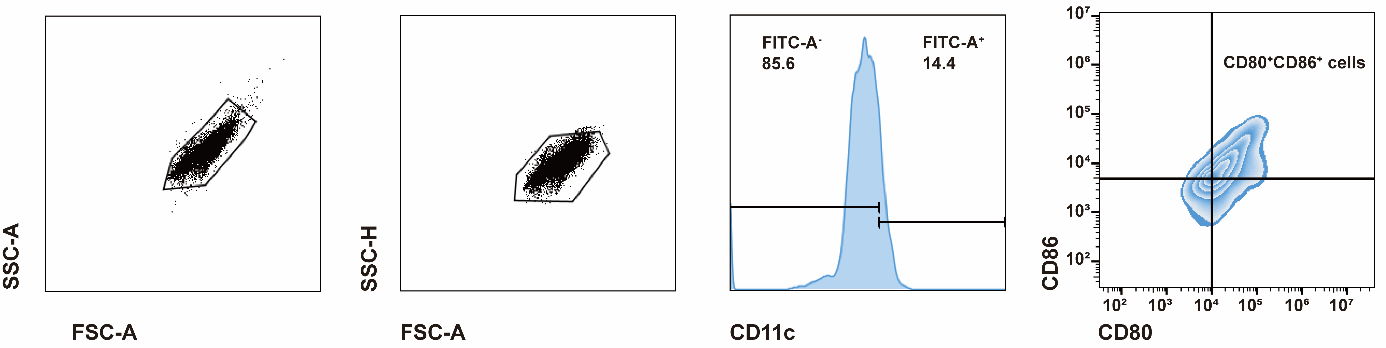


***Figure S22*** Gating strategy to analyze matured DCs (CD80^+^CD86^+^) in tumors.

***17.4 In vivo evaluation of T cell populations***

At the end of treatment, 4T1 tumor-bearing mice from each group were euthanized. Tumors, spleens, and peripheral blood were collected, and T cells were isolated for T cell isolation. Spleens were harvested and treated with ACK lysis buffer to remove red blood cells. Primary tumor tissues were dissected, finely minced, and digested in PBS containing 1 mg/mL collagenase type IV for 4 h at 37 °C. Resulting cell suspensions were filtered through a 200-μm mesh nylon strainer and treated with erythrocyte lysis buffer to obtain a single cell suspension. Finally, all cell suspensions were incubated with anti-CD3-FITC, anti-CD4-PerCP, and anti-CD8a-PE antibodies for 30 min and analyzed by flow cytometry.


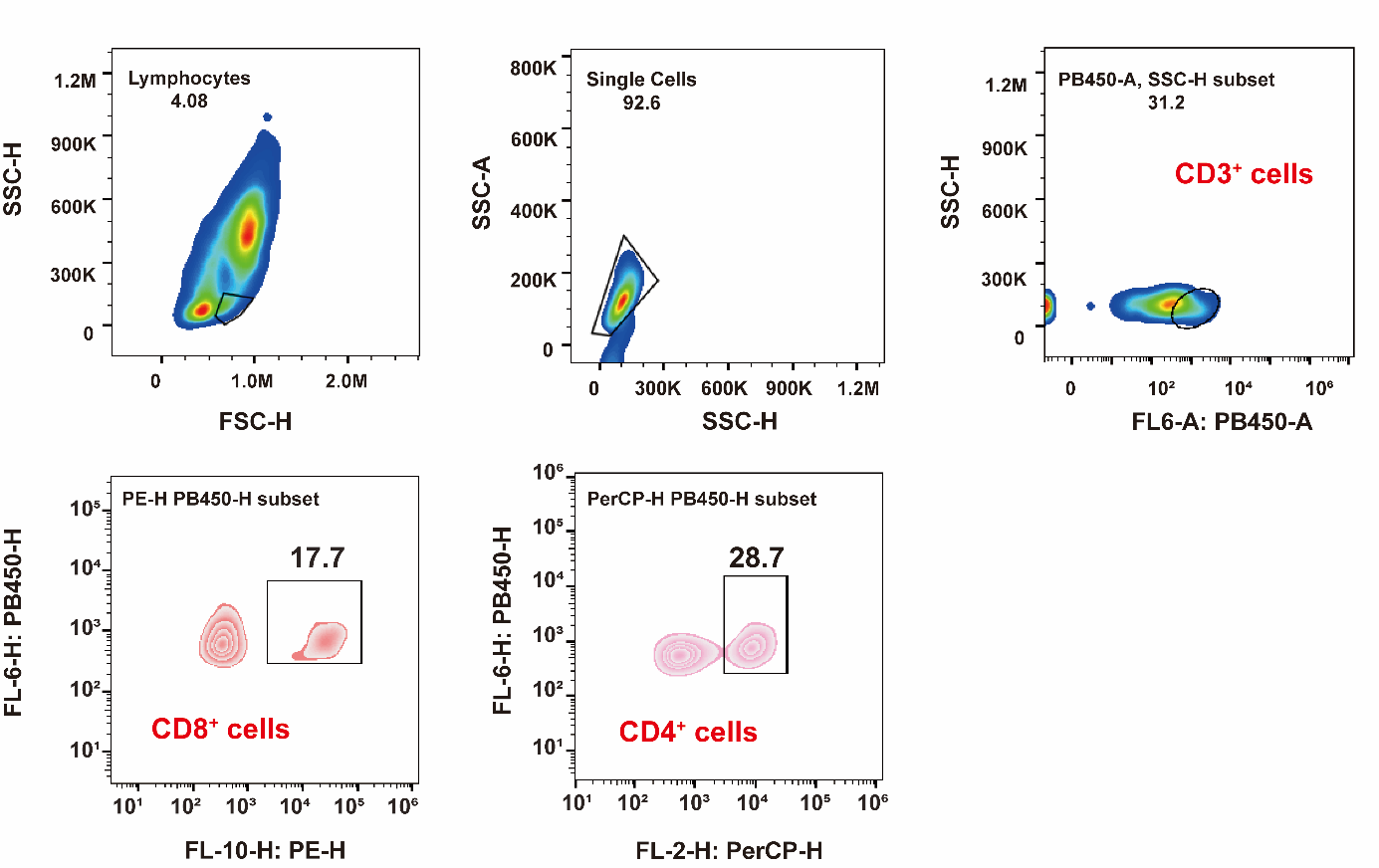


***Figure S23*** Gating strategies for flow cytometry analysis of CD3^+^CD8^+^ T cells and CD3^+^CD4^+^ T cells.


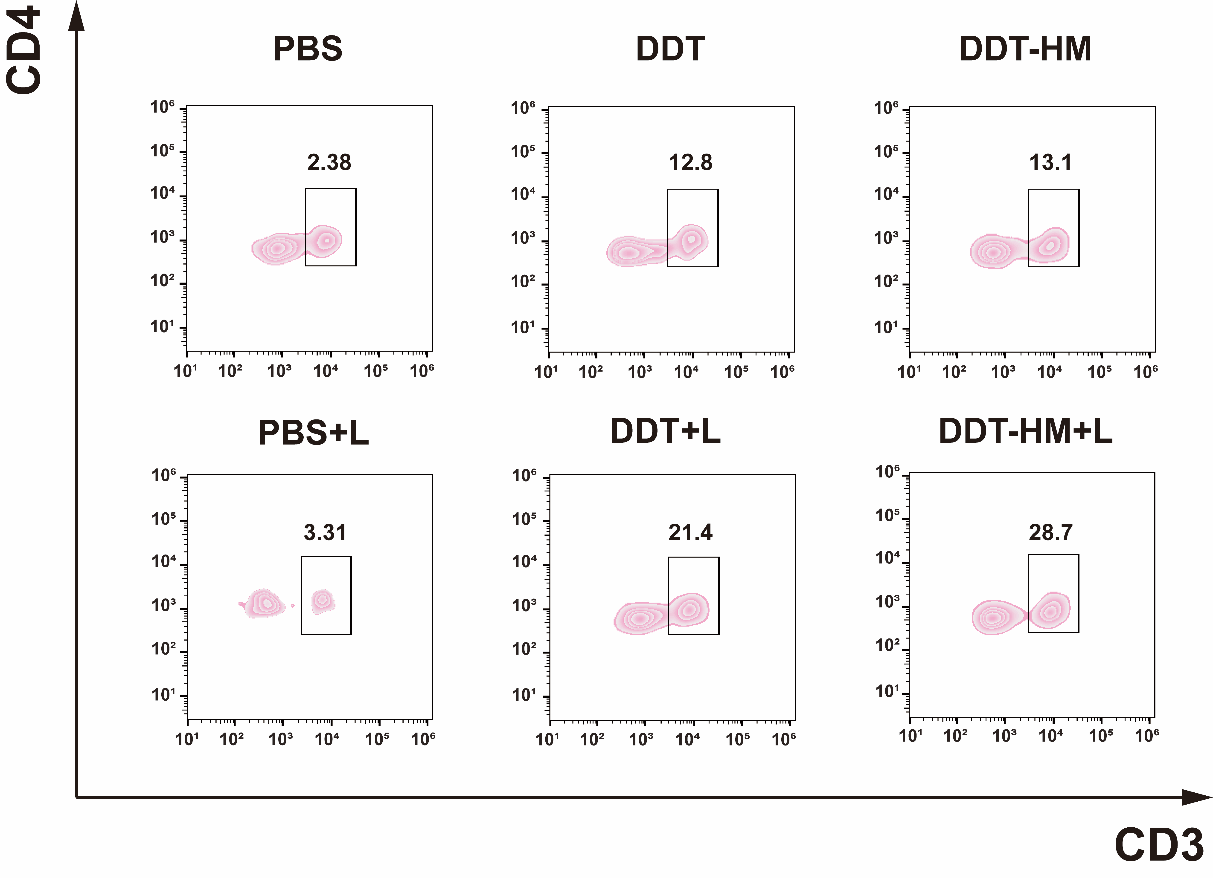


***Figure S24*** Peripheral blood immune profiling: (a) CD4^+^ T helper cells were screened from CD3^+^ T lymphocytes.


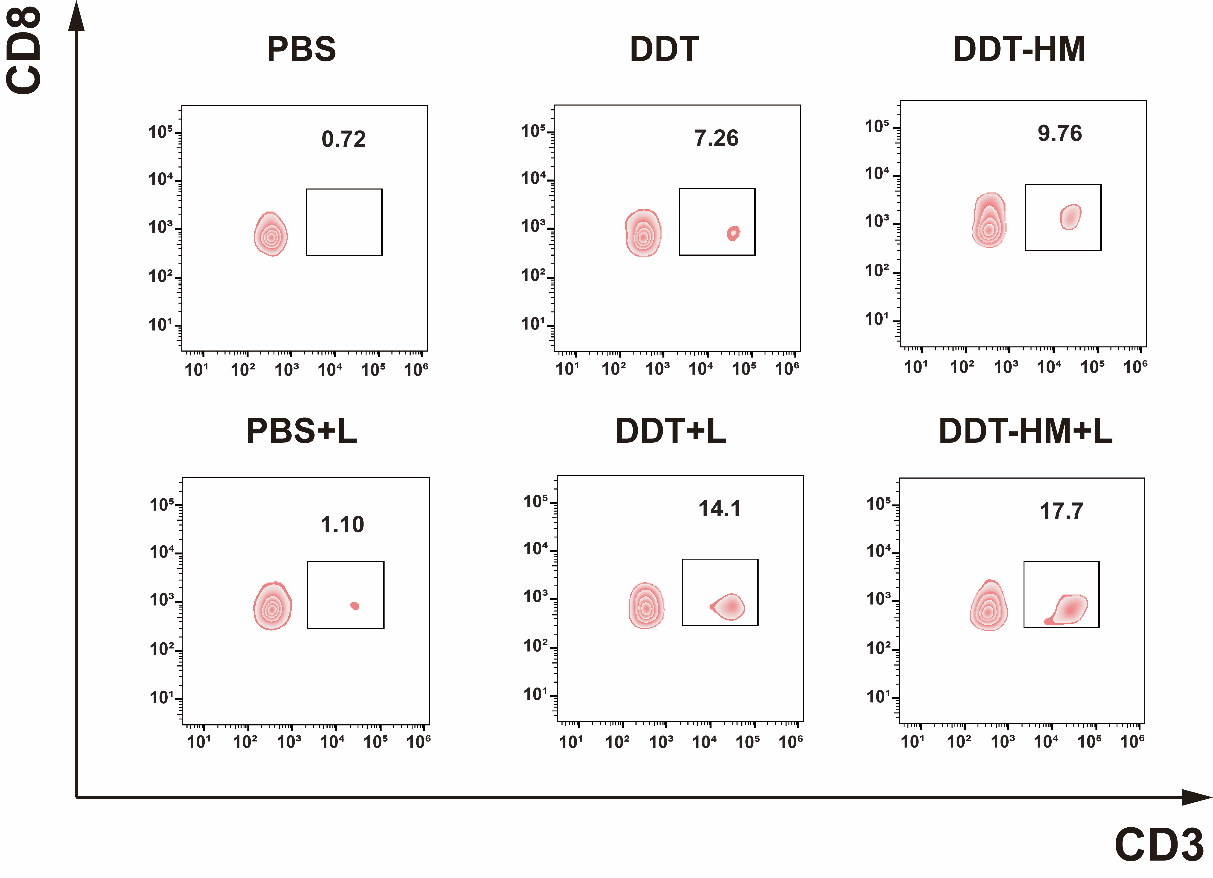


***Figure S25*** Peripheral blood immune profiling: (a) CD8^+^ T helper cells were screened from CD3^+^ T lymphocytes.


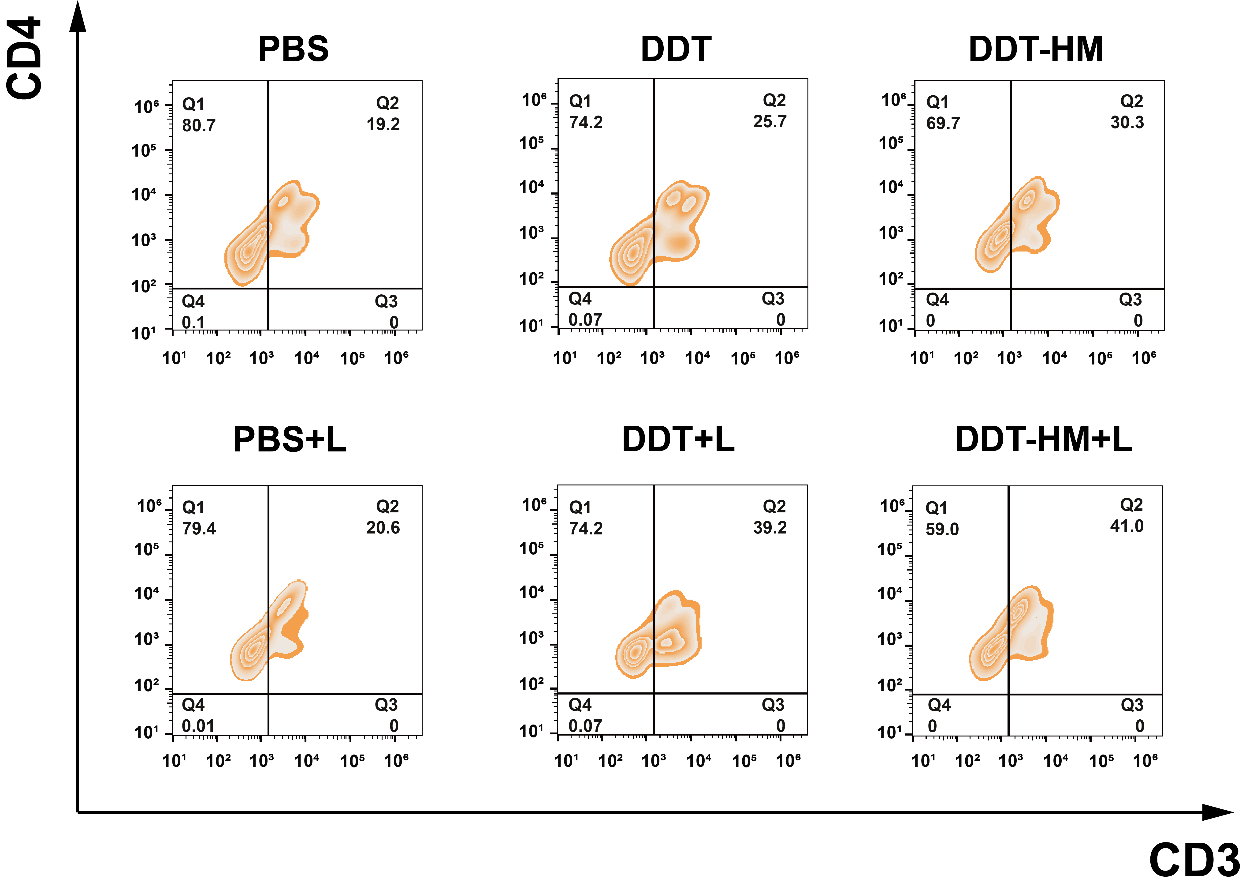


***Figure S26*** Peripheral spleen immune profiling: CD4^+^ T helper cells were screened from CD3^+^ T lymphocytes.


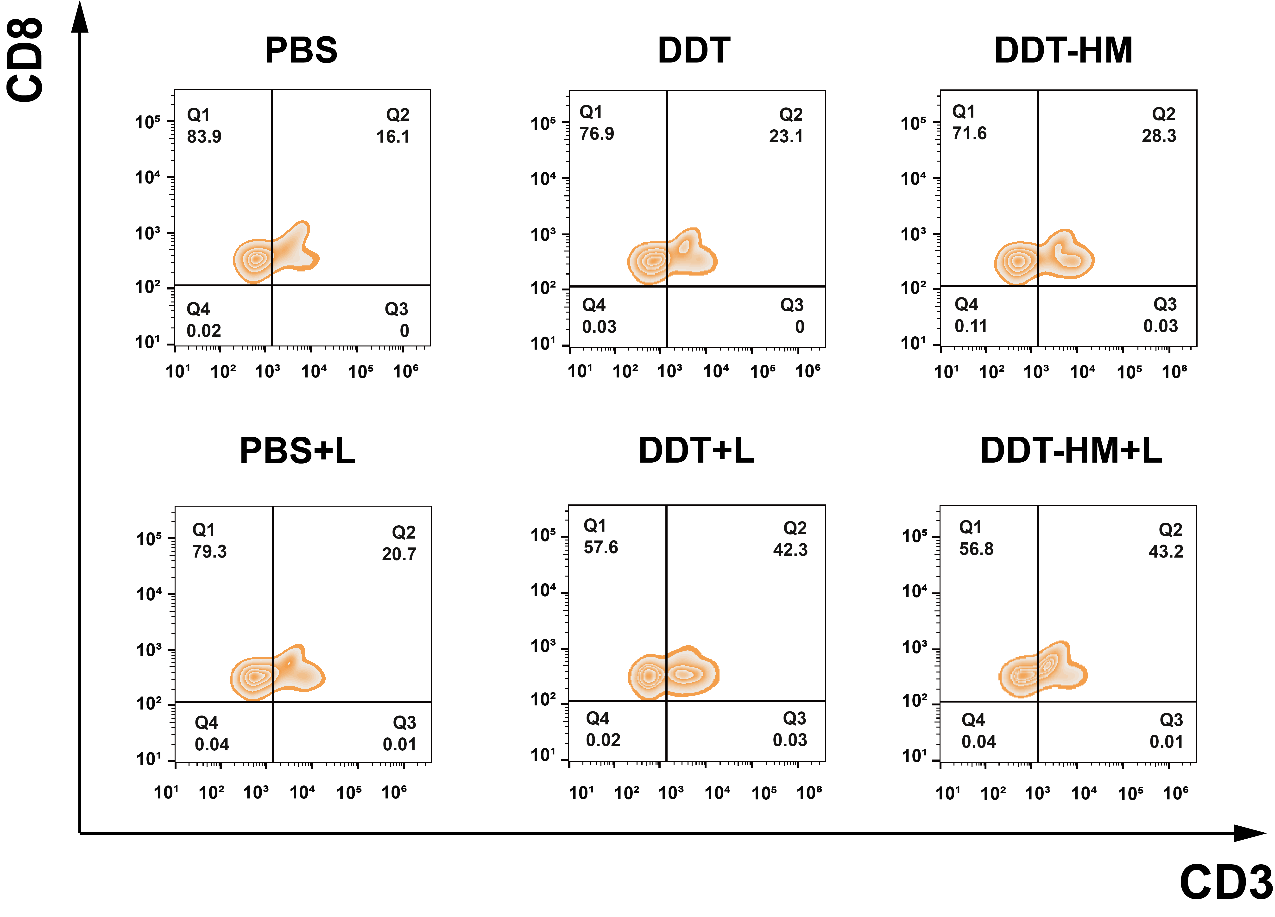


***Figure S27*** Peripheral spleen immune profiling: CD8^+^ cytotoxic T cells were screened from CD3^+^ T lymphocytes.


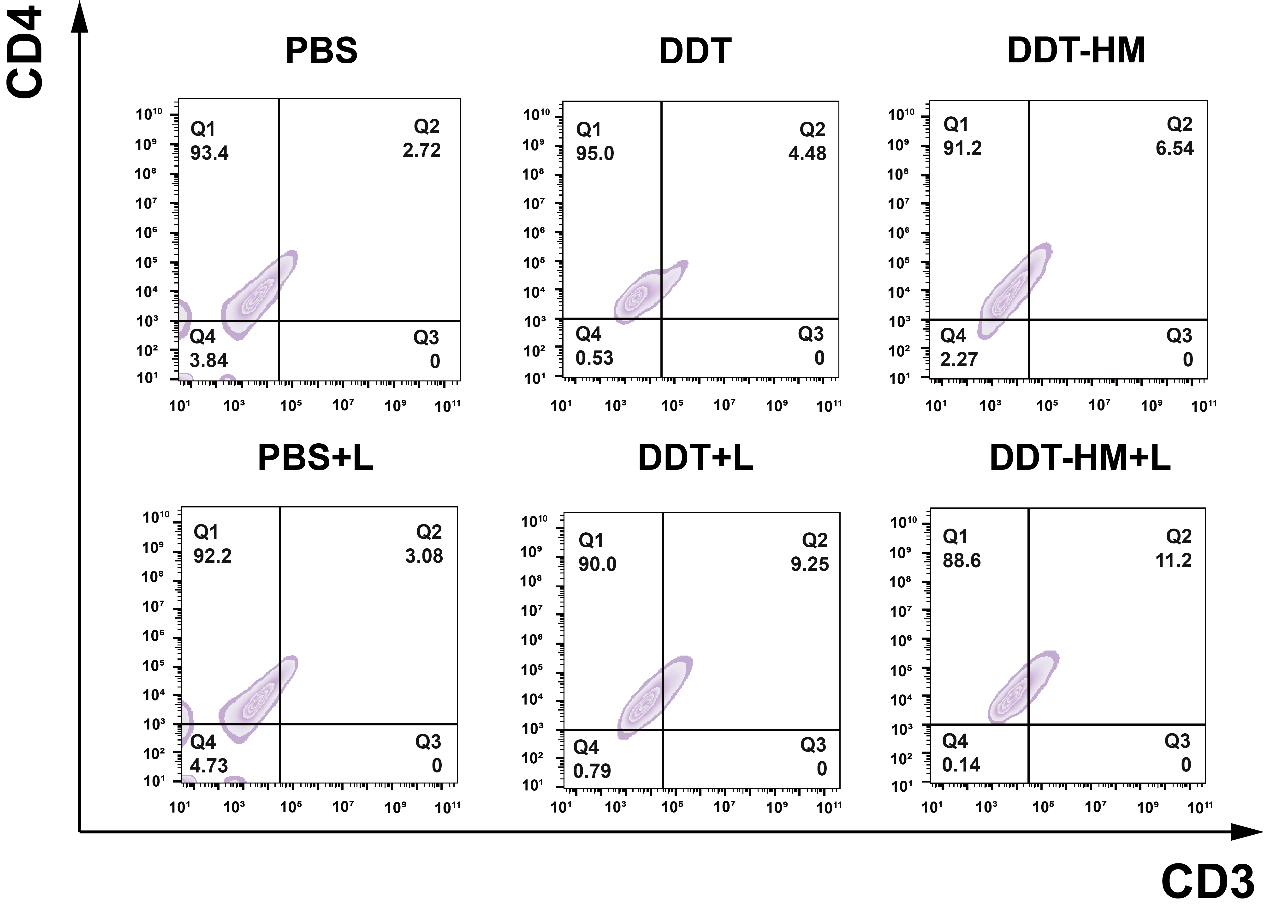


***Figure S28*** Peripheral tumor immune profiling: CD4^+^ T helper cells were screened from CD3^+^ T lymphocytes.


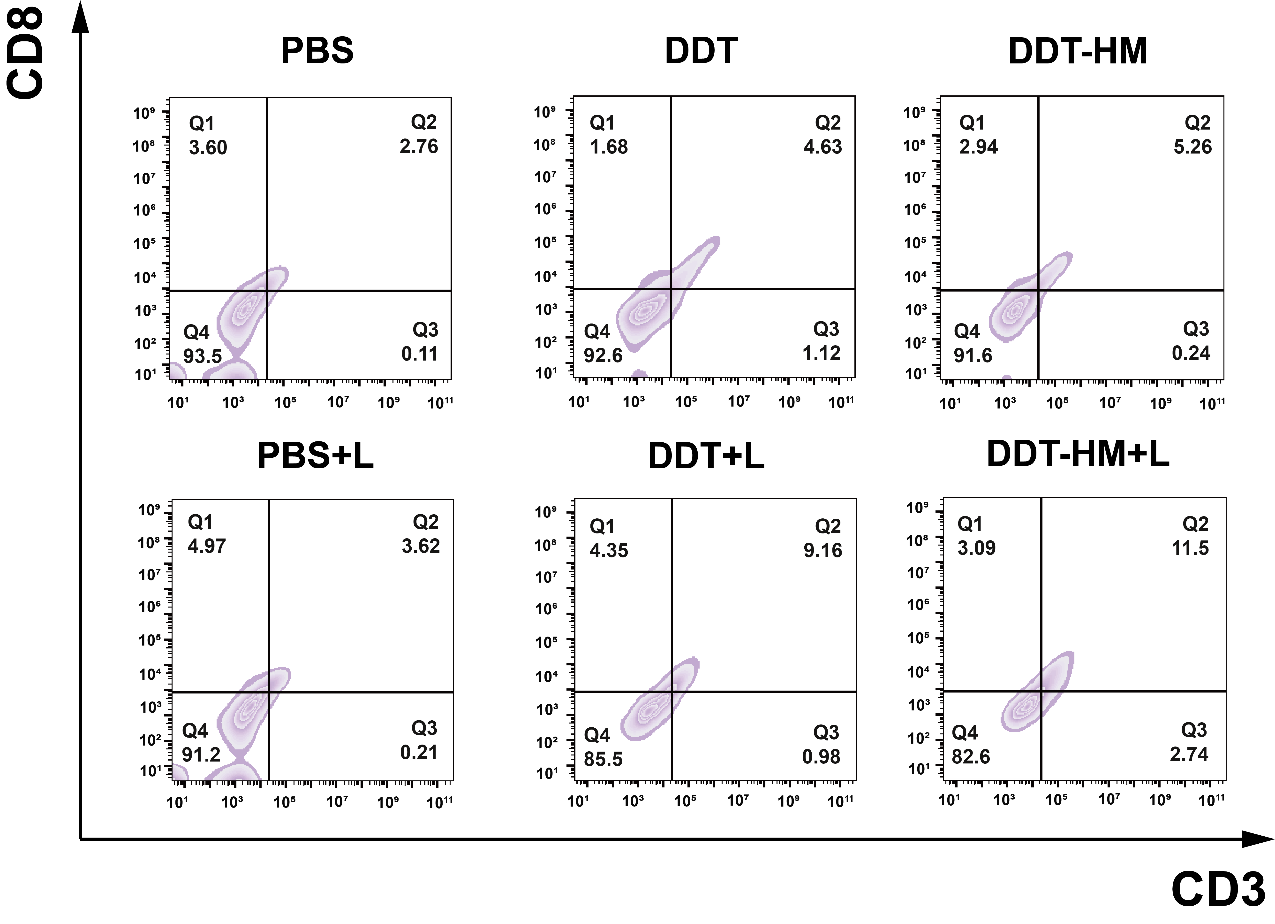


***Figure S29*** Peripheral tumor immune profiling: CD8^+^ cytotoxic T cells were screened from CD3^+^ T lymphocytes.


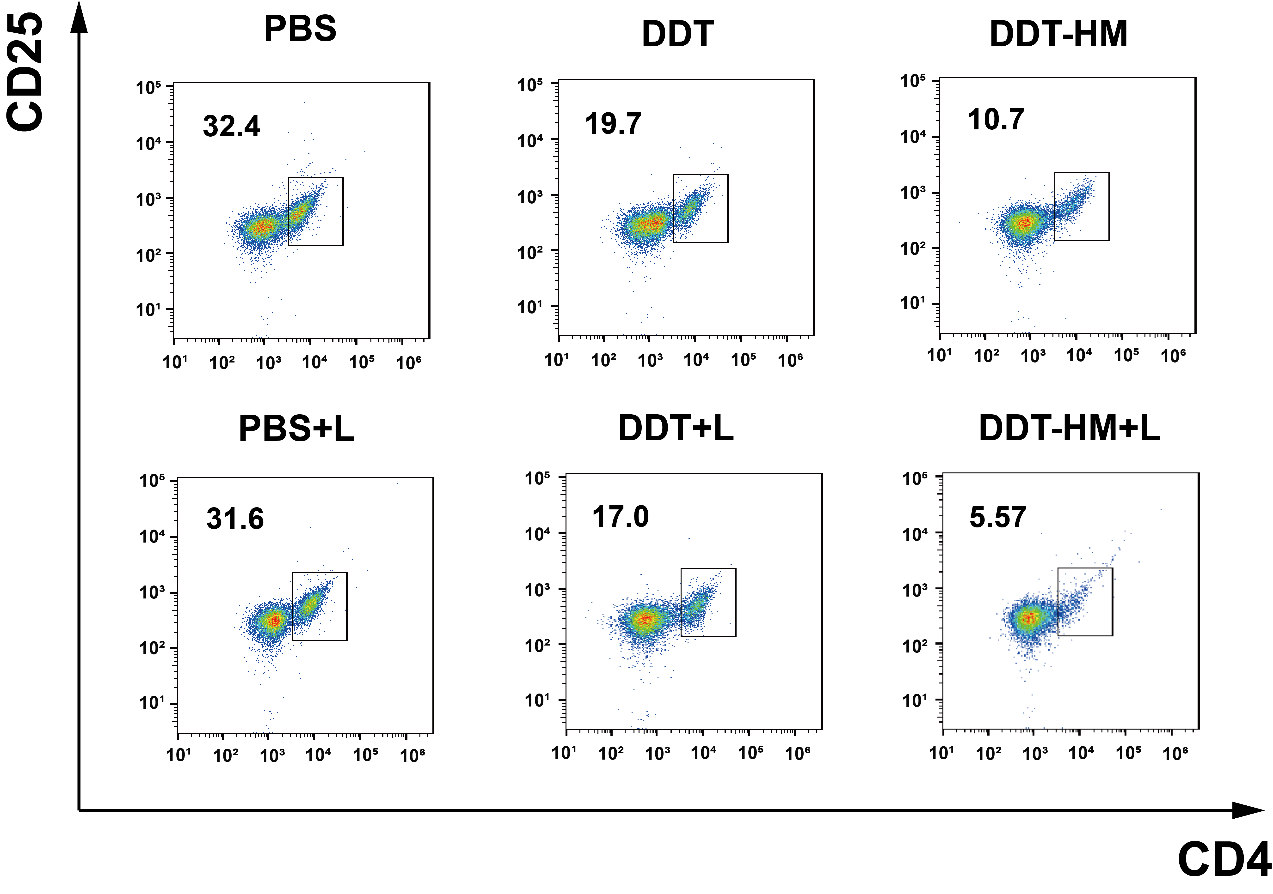


***Figure S30*** Peripheral spleen immune profiling: screening T_reg_ cells in the spleen.

***17.5 In vivo detection of cytokine secretion levels***

Cytokine levels (IL-6, TNF-α, IFN-γ and IL-10) in T-cell supernatants were quantified by ELISA. Standard cytokine solutions and T-cell supernatant samples were diluted to appropriate concentrations. Aliquots (100 μL) was added to each well, followed by 50 μL of antibody working solution prepared per manufacturer's protocol. After sealing with adhesive film, plates were incubated at 37°C for 90 min. Wells were then aspirated and washed with 300 μL wash buffer (1 min incubation, repeated 4×). Streptavidin-HRP working solution (100 μL/well) was added, and plates were resealed and incubated at 37°C for 30 min. Following another wash cycle (as above), 100 μL TMB substrate solution was added per well and incubated at 37 °C in the dark for 10 min. Reactions were terminated with 100 μL stop solution per well, and absorbance was measured using a microplate reader. Cytokine concentrations were calculated against standard curves.

***17.6 In vivo STING pathway activation analysis***

Tissue lysates were prepared using RIPA buffer supplemented with phosphatase inhibitors (100:1) and protease inhibitors (PMSF), kept at 4°C. Tissue samples from each group were weighed, minced, and rinsed repeatedly with ice-cold PBS. After blotting dry, tissues were transferred to pre-chilled tubes with grinding beads containing cold lysis buffer. Homogenization was performed in pre-chilled tubes with grinding beads and cold lysis buffer at 4°C until complete tissue disruption. Homogenates were transferred to fresh tubes, incubated at 4°C for 30 min, then centrifuged at 12,000 ×g (4°C, 25 min). Supernatants were collected for Western blot analysis of STING pathway activation.

**
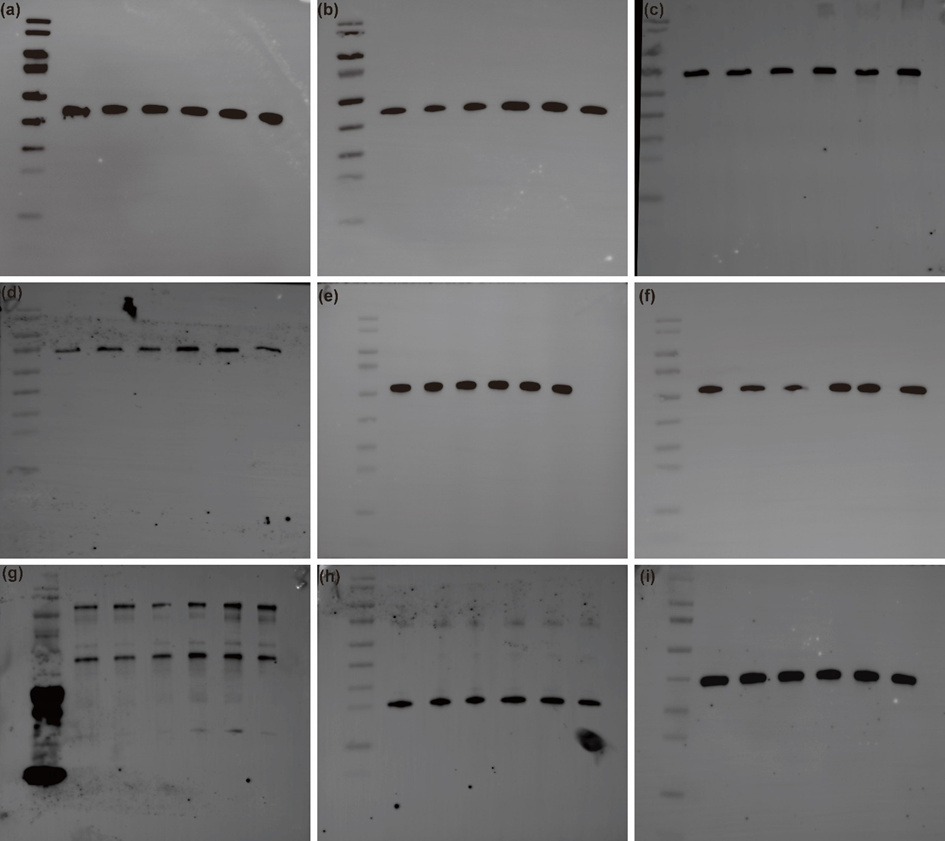
**

***Figure S31*** Western blot of STING pathway components (a: STING, b: p-STING, c: TBK1, d: p-TBK1, e: IRF3, f: p-IRF3) and ICD markers (g: CRT, h: HMGB1) in tumor lysates harvested 15 d post-treatment. i: GAPDH as control. The order from left to right is PBS, PBS + L, DDT, DDT + L, DDT-HM, DDT-HM + L.


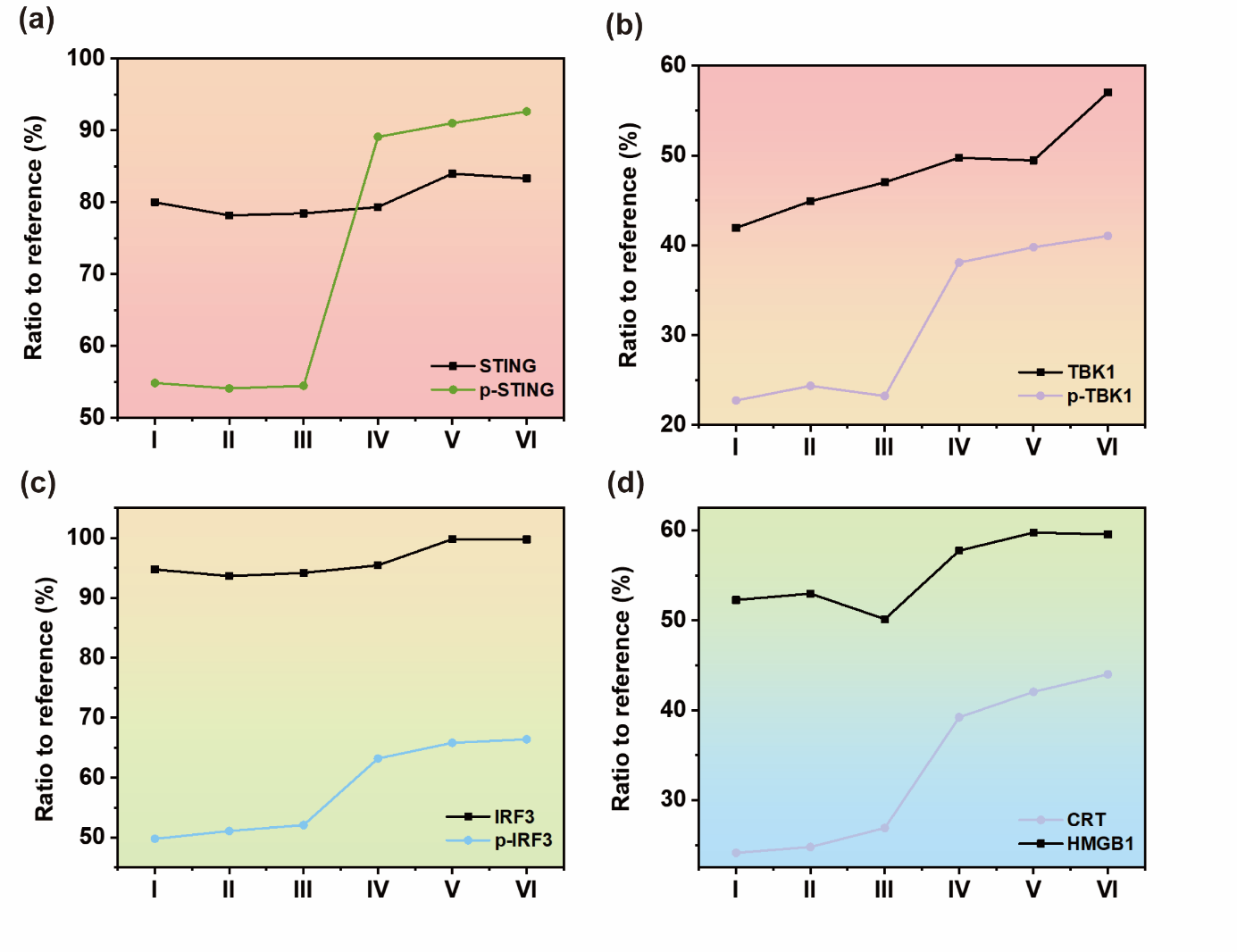


***Figure S32*** Relative protein expression analysis. Grayscale intensities normalized to GAPDH. I: PBS, II: PBS + L, III: DDT, IV: DDT + L, V: DDT-HM, VI: DDT-HM + L.

***18. Anticancer vaccination***

Frequencies of central memory (T_cm_) and effector memory T cells (T_em_) in blood and tumors were quantified. BALB/c mice (*n* = 6/group) were randomly assigned to PBS control or **DDT-HM** nano-vaccine groups. Mice received two intravenous immunizations (38 μg/kg **DDT-HM** in 100 μL PBS or PBS vehicle) at 7-d intervals. Twenty-four hours post-boost, 4T1 cells (5 × 10^5^) were inoculated subcutaneously in the dorsal flank. Tumor dimensions were measured every 48 h starting at day 7 post-implantation for 25 d.

***18.1 In vivo systemic toxicity evaluation***

Blood samples were analyzed for biochemical parameters (including ALP, ALB, AST, ALT, BUN, Cr) and complete blood counts. No statistically significant differences were observed between treatment groups and PBS controls, indicating preserved hepatic and renal function.


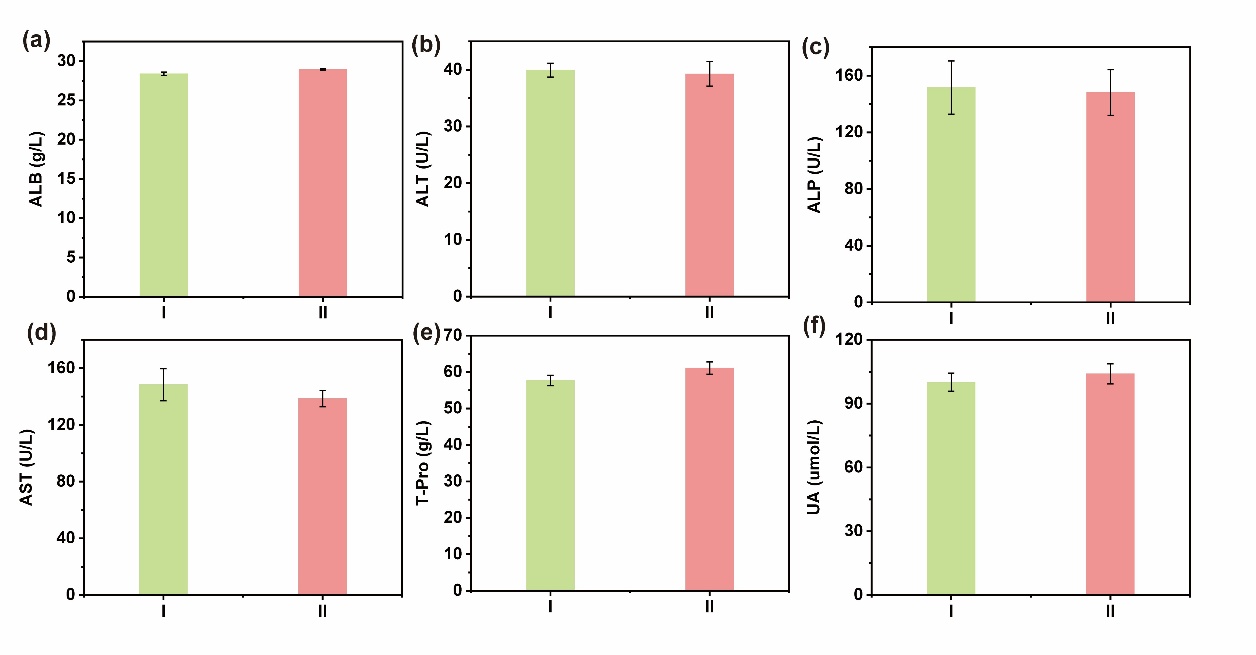


***Figure S33*** Blood routine and biochemical analysis of mice after different treatments (I: PBS; II: **DDT-HM** NPs).


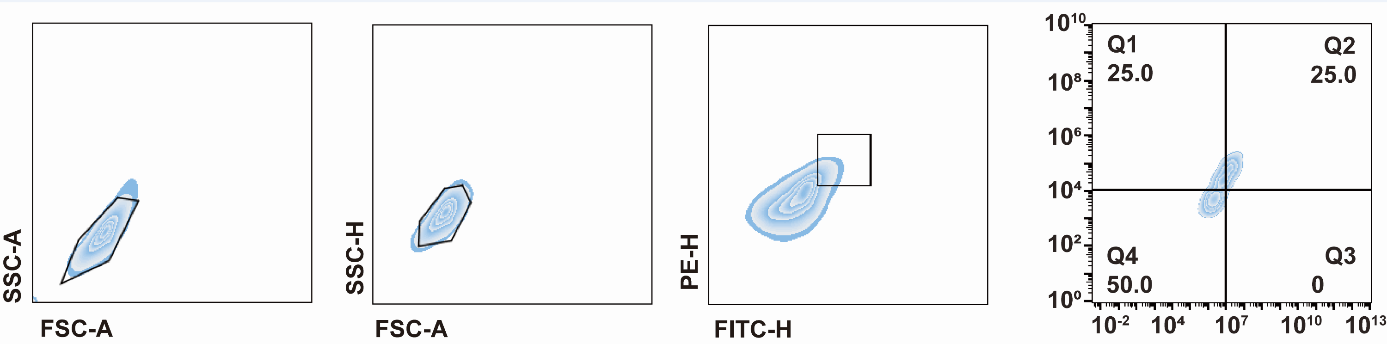


***Figure S34*** Gating strategy to analyze CD3^+^CD8^+^ T-cell memory subsets in blood and tumors.
